# Genetic diversity loss in the Anthropocene will continue long after habitat destruction ends

**DOI:** 10.1101/2024.10.21.619096

**Published:** 2024-10-22

**Authors:** Kristy S. Mualim, Jeffrey P. Spence, Clemens Weiß, Oliver Selmoni, Meixi Lin, Moises Exposito-Alonso

**Affiliations:** 1 Department of Plant Biology, Carnegie Institution for Science, Stanford, California, USA; 2 Department of Biology, Stanford University, Stanford, California, USA; 3 Department of Integrative Biology, University of California Berkeley, Berkeley, USA; 4 Department of Genetics, Stanford University, Stanford, California, USA; 5 Stanford Cancer Institute, Stanford University, Stanford, California, USA; 6 Department of Global Ecology, Carnegie Institution for Science, Stanford, California, USA; 7 Howard Hughes Medical Institute, University of California Berkeley, Berkeley, USA

## Abstract

Genetic diversity within species is the basis for evolutionary adaptive capacity and has recently been included as a target for protection in the United Nations’ Global Biodiversity Framework (GBF). However, there is a lack of reliable large-scale predictive frameworks to quantify how much genetic diversity has already been lost, let alone to quantitatively predict future losses under different conservation scenarios in the 21st century. Combining spatio-temporal population genetic theory with population genomic data of 18 plant and animal species, we studied the dynamics of genetic diversity after habitat area losses. We show genetic diversity reacts slowly to habitat area and population declines, but lagged losses will continue for many decades even after habitats are fully protected. To understand the magnitude of this problem, we combined our predictive method with species’ habitat area and population monitoring reported in the Living Planet Index, the Red List, and new GBF indicators. We then project genetic diversity loss in 13,808 species with a short-term genetic diversity loss of 13–22% and long-term loss of 42–48% with substantial deviations depending on the level of habitat fragmentation. These results highlight that protection of only current habitats is insufficient to ensure the genetic health of species and that continuous genetic monitoring alone likely underestimates long term impacts. We provide an area-based spatio-temporal predictive framework to develop quantitative scenarios of global genetic biodiversity.

Genetic diversity dictates a species’ ability to adapt to new environmental conditions^1^ and thus was coined one of the three key dimensions of biodiversity at the 1992 United Nations’ Convention of Biological Diversity (CBD)^2^; along with species and ecosystem level biodiversity. After being overlooked in global biodiversity framework texts for decades^3^, the 2022 Kunming-Montreal UN CBD convention finally ratified a Global Biodiversity Framework (GBF, CBD/COP/DEC/15/4, CBD/COP/DEC/15/5) that explicitly recognizes to “*maintain and restore the genetic diversity within and between populations of native, wild and domesticated species to maintain their adaptive potential*”^4^.

Conservation genetic approaches have focused on species at high extinction risk-e.g. those species where the last populations and individuals remain-by managing populations to prevent complete depauperation of genetic diversity and avoid inbreeding, which leads to further extinction risk. For instance, helpful prescriptions include ensuring species have >500 effective individuals $\left(N_{e}\right)$ to safeguard genetic diversity over generations from stochastic genetic drift or *ex situ* breeding programs that match individuals with different genetic variation^5^. One may then conclude that species (or their component populations) with $N_{e}>500$ may not be at risk of losing genetic diversity. To the contrary, many non-threatened species have suffered major geographic area range and population losses^6,7^, which we expect must have major consequences of genetic diversity losses^8–10^. The new post-2020 global biodiversity framework aims to protect all species (not only threatened ones) from genetic diversity loss and to maintain this key genetic dimension of biodiversity for present and future generations. However, while new genomic tools are applied to an ambitiously growing numbers of species^11–13^, global predictive frameworks and estimates of genetic diversity loss across species are still inexistent, let alone methods to project temporal trajectories of genetic diversity in the 21^st^ century.

To monitor and protect genetic diversity across large numbers of species and over time, global conservation efforts have focused on proxy or qualitative *indicators* based on present species’ population sizes and habitat areas. Such proxies are based on the biological insight that the number of individuals within a species and its geographic distribution correlates with the genetic diversity of a species^14,15^. For example, species that are categorized as threatened, with rapid declines in their habitat or population sizes, often have lower genetic diversity-at least in vertebrates and mammals which are mostly studied^14,16,17^. A proxy headline indicator adopted in the Global Biodiversity Framework proposed by the GEO-BON genetics working group is the proportion of populations with effective population size $\left(N_{e}\right)$ below 500, following the legacy of conservation genetics of threatened species. A second complementary indicator includes the total fraction of populations within a species that has been lost with respect to a recent past baseline. A third indicator of monitoring efforts of a country is the number of species with any genetic data. These three indicators were showcased recently in an effort to evaluate 100+ species for 9 countries^18^. It has also been proposed that already-implemented large population monitoring of species could serve as proxies: One such coordinated effort is the Red List (IUCN), which has classified ~80,000 animal, fungi, and plant species into non-threatened, vulnerable, endangered, and critically endangered, using several thresholds of rapid species decline (i.e. few individuals, contracting geographic area, etc.). A second coordinated effort is the Living Planet Index^19^, also a GBF indicator, which monitors 41,986 populations of 5,579 terrestrial animal [vertebrate] species.

These proxy indicators for genetic diversity are based on demographic rather than genetic DNA data, and thus deemed feasible, cheap, and scalable. However, by design they do not provide a quantitative metric of DNA diversity nor inform about future numerical losses. To calculate quantitative genetic metrics requires DNA sequencing of at least partial or entire genomes for multiple individuals or populations of a species. Such metrics include the number of DNA mutations within a species (i.e. allelic richness or segregating sites [S]), or average nucleotide genetic diversity (i.e. average number of mutation differences between two individuals of a species or nucleotide diversity [π]).

To bridge the gap that conservation policy is often proxy-based through area- or population metrics, we recently described a mutations-area relationship (MAR) power law: $M=cA^{\text{zmar}}$. This links allelic richness or segregating sites or number of mutations (M) and its geographic range area (A) through a $z_{MAR}$ parameter characterizing spatial structure^8,20^ (note we avoid S notation for segregating sites to avoid confusion with the species area equation, SAR). Re-arranged, the equation $({\left.A_{\text{present}}/A_{\text{past}}\right)}^{\text{zmar}}$ quantitatively predicts the percentage loss of genetic diversity with a percentage habitat area reduction within a species $\left(A_{\text{present}}/A_{\text{past}}\right)$, and could in principle translate proxy indicators into quantitative genetic diversity metrics. However, it is still unclear how different genetic diversity metrics behave in continuous geographic ranges, how reactive they are to area losses, and what will be the long term consequences that such impacts and genetic drift will have on the genetics of a species.

To develop predictions of genetic diversity metric losses on complex geographic landscapes for the 21st century, we built new spatio-temporal population genetic theory and continuous space forward-in-time simulations informed and validated by a large genome-wide DNA sequencing database of 7,437 geo-referenced individuals from 18 plant and animal species. Although classic population genetics has described genetic diversity at equilibrium through frameworks such as mutation-drift balance^21,22^, migration-drift equilibrium in spatial contexts^23,24^, and the effects of population bottlenecks^25^, these models fall short in capturing the temporal non-equilibrium dynamics of genetic diversity, especially in complex geographic landscapes undergoing landscape alteration. We then built a standard Wright-Fisher population genetic model with an arbitrary number of subpopulations in a two dimensional geographic grid connected by migration, i.e. a meta-population. To calculate genetic diversity, we use the fact that the average differences between two sampled individuals can be derived from allele frequencies of a subpopulation or group of populations. The key insight is that the dynamics of genetic diversity depend only on the first and second moments of allele frequencies across subpopulations over time, which obey a linear system of ordinary differential equations, even when we alter landscapes and remove subpopulations due to fragmentation (hereon termed *WFmoments*, see details in Mathematical Appendix). Solving these we can thus compute expected genetic diversity over time across a landscape. In parallel, we validated these insights through a simulation-based framework using SLiM v4.1^26^, under which we model non-Wright Fisher dynamics in a two dimensional geographic continuous space incorporating both age and spatial structure during mating (see **Materials and Methods**, Text S1, Fig. 1–3). Differently from other simulation approaches in conservation genetics^27,28^, we designed our framework to incorporate a spatial component and of abundant, broadly distributed species rather than study dynamics of very small populations (e.g. simulation landscape runs are ${\text{N}}_{\text{e}}=5000-20000$ individuals, and theory landscapes can be arbitrarily large, e.g. $\Theta =4{\text{N}}_{\text{e}}\mu =0.01;{\text{N}}_{\text{e}}\approx 2\times 10^{6}$). With this in hand, we studied and simulated species with different population sizes and geographic landscapes, and evaluated the consequences to genetic diversity with different habitat and population loss scenarios (Fig. 1A, Fig. S1–2, S7).

We first asked how increasing spatial habitat destruction immediately impacts genetic diversity on a short-term timescale. A simple approach to simulate habitat destruction is removing area from one extinction edge, hereon termed edge contraction (Fig. 2). We found short-term loss of genetic diversity lags behind area loss in a predictable manner (simulation results predicted by *WFmoments*: ${\text{R}}^{2}=0.98$, $P<1\times 10^{-16}$, n =81 Fig. 2A). Such lag is determined by the migration and gene flow rates of the species in the landscape, i.e. the spatial population genetic structure (Fig. S2). For instance, a panmictic species with infinite gene flow and thus no population structure (${\text{F}}_{\text{ST}}\approx 0$) suffers only a 4.7% (95% CI = 4.5–4.9%) instantaneous nucleotide genetic diversity loss with 50% of habitat loss (Fig. 2B), while a species with high population structure (e.g. ${\text{F}}_{\text{ST}}=0.9$) would suffer an instantaneous 9% (95% CI = 7.9–10.0%) nucleotide genetic diversity loss (Fig. 2B). These results are robust to the dimensions of the landscape (e.g. a 1 dimension landscape such as a river system, Text S2, Fig. S25) or to step-wise loss such as a 1% habitat loss per generation rather than instantaneous complete habitat loss (Fig. S1), suggesting short-term genetic diversity under straightforward edge contraction remains robust to the rate and shape of habitat loss. This recapitulates our power law findings of the mutations-area relationship (MAR) originally developed for allelic richness^8,20^ (Text S4). As suspected, in a species landscape of moderate population structure (${\text{F}}_{\text{ST}}=0.3$) a power law with a scaling $z_{\text{GDAR}}\sim 0.03$ fits also a nucleotide genetic-diversity-area relationship (GDAR), with high accuracy (${\text{R}}^{2}=0.96$) and a MAR with a scaling of $z_{MAR}=0.3$ $\left({\text{R}}^{2}=1.0\right)$ (Fig. S2, Table S1). Simulating extinction of sampled populations of empirical datasets of 16 plant and animal species also confirms MAR and GDAR have predictability in real world species (mean population structure of ${\text{F}}_{\text{ST}}=0.26$, mean accuracy ${\text{R}}^{2}=0.677$, range 0.10–0.98, Table S14). Our simulations and the power law intuitively explain why there is a lag of genetic diversity loss, as a power law that approaches z→0 (i.e. low population structure) indicates genetic diversity should be relatively insensitive to habitat area losses: ${\left(A_{\text{present}}/A_{\text{past}}\right)}^{z\to 0}\approx 1$ (Fig. 2A, Fig S2–3). Together, the MAR and GDAR power laws could thus be readily applicable to predict short-term losses of allelic richness and nucleotide diversity, respectively, across large numbers of species whose habitat is monitored.

While it is expected that habitat loss will also have long-term impacts on genetic diversity, its magnitude in complex landscapes remains poorly understood. A classic prediction from population genetics assuming a single panmictic population (i.e. no spatial structure) is that the expected nucleotide diversity for diploids in equilibrium follows^29,30^: $E[\pi ]=4N_{e}\mu$. If area or population abundance represents $N_{e}$, a reduction in population size would thus proportionally reduce π at a long-term equilibrium. By utilizing WFmoments to measure nucleotide diversity over time and tracking simulated populations for ~20,000 generations, we investigated the non-equilibrium dynamics of genetic diversity loss just after habitat loss stops, monitoring its progression until a final equilibrium is reached (Fig. 1–2, S4). We found that under edge contraction and in a species with no population structure resembling classic population genetics $\left({\text{F}}_{\text{ST}}\approx 0\right.)$ the long-term equilibrium roughly follows and surpasses such classic expectations: a ~50% area loss roughly results in a ~50% long-term genetic diversity π loss (Fig. 2B), with similar trends for allelic richness (Fig. S14). Counterintuitively, while increasing population structure led to larger short-term genetic diversity loss (Fig. 2B; yellow arrows), we found the opposite for long-term loss (Fig. 2B; **red arrows**, see theoretical interpretation Text S3); likely because structured landscapes can generally hold larger amounts of genetic diversity. Long-term genetic diversity loss equilibrium is unlikely to be reached in the near-term timeline of conservation policies for 2050. For instance, even for relatively fast growing organisms, from fruit flies (~10–20 generations per year), to annual plants (~1 generation per year), to fast growing trees (~15 years per generation), the remaining ~25 years would correspond to ~400, ~25, and ~2 generations respectively (see scales in Fig. 1B). While long-term equilibrium predictions may be less relevant to conservation than short-term ones, a worrisome conclusion of this work is that such long-term losses are very dramatic. Even if conservation practices succeed in stopping habitat destruction, most organisms will continue losing genetic diversity for decades and centuries to come.

To avoid dramatic long-term losses, we will need to restore habitat area and population sizes within species. The buildup of genetic diversity through new mutations is incredibly slow. Using our SLiM framework, we simulated two scenarios: a habitat recovery (letting populations naturally colonize it) and population restoration (individuals across the intact landscape are moved to the restored habitat) (Fig. S26, Text S5). With this, we were able to see a significant lag in genetic diversity recovery (Fig. S27–28). For short-lived species, similarly to long-term losses, recovery is faster. For slow growing species, we must reduce initial demographic impacts and restore populations as soon as possible as recovery will be unlikely in the next centuries.

Habitat destruction and geographic area losses within a species may not occur from an edge. Rather, habitat may become fragmented, which poses unique risks for species^31^. Fragmentation alters gene flow, and increases isolation and exposure of habitat edges to genetic drift. Using our SLiM and WFmoments frameworks, we randomly remove 1/100^th^ fraction dividing the species habitat in a 10 × 10 grid where isolated populations have no gene flow with neighbors (Fig. 1B, 3A, Fig. S7). Simulations showed species-wide nucleotide genetic diversity is highly insensitive to fragmentation in the short-term and in fact dramatically increases in the long-term: for instance, with ~90% habitat loss, we observed an increase of 263% of nucleotide genetic diversity (range: 100–263%; also observed in allelic richness, Fig. S13). While seemingly counter-intuitive, this is a longstanding observation in population genetics coined the “Wahlund effect”, where lack of gene flow between two previously connected populations leads to genetic drift that fixes independent alleles, causing populations to become more distinct. Thus, increased diversity is observed if populations are pooled^32,33^ (Fig. S12, Fig. S13). This genetic diversity inflation could consequently be predictable, and in fact, the WFmoments expectations in fragmented 2D landscapes recapitulate diversity trends in simulations (${\text{R}}^{2}=0.46$, $P=4.46\times 10^{-244}$, n=121 Fig. 2A, Fig. S6). This inflation effect is repeatable with smaller (1/400^th^) and larger (1/12^th^) fragmentation, and is explained by landscape fragmentation metrics of the different simulations, including total core area (${\text{R}}^{2}=0.76$, $P=1.62\times 10^{-25}$), spatial connectedness $\left({\text{R}}^{2}=0.87,P=1.11\times 10^{-35}\right)$, the general perimeter of patches $\left({\text{R}}^{2}=\right.\left.0.53,P=2.49\times 10^{-14}\right)$, and patch and edge density$\left({\text{R}}^{2}=0.63,P=2.12\times 10^{-18}\right)$ (Fig. S10).

Although fragmentation and isolation of populations in a species may cause them to diverge and inflate species-wide genetic diversity, this is hardly indicative of good genetic health. In fact, by quantifying genetic diversity within population fragments in our simulations, we detect a small but significant reduction in the short term with broad confidence intervals (linear regression b=-27.0, $P<1.0\times 10^{-16}$, ${\text{R}}^{2}=0.38$, Fig. 3B), and a large long-term reduction (b=-87.4, $P<1.0\times 10^{-16}$, ${\text{R}}^{2}=0.67$, Fig. 3B, Fig. S14), with more dramatic results for allelic richness (Fig. S14, Fig. S15). The implications of our study of species area reductions with severe isolations are twofold. First, genetic diversity protection goals and indicators within the Global Biodiversity Framework must include explicit guidelines on how genetic diversity metrics are calculated, and these metrics have to be spatially-aware (i.e. genetic diversity computed in individuals from different isolated populations may result in misleading values). Second, the combination of area loss and fragmentation may challenge the application of easy-to-use power law equations, MAR and GDAR, which use only area loss. In contrast, the *WFmoments* framework can achieve accurate predictions of within- and across-population diversity if census monitoring frameworks not only focus on population and area sizes but also provide landscape connectivity metrics.

Finally, we use our power law functions GDAR/MAR and *WFmoments* approximation tables to estimate global genetic diversity loss due to land use change and habitat destruction to date. Although accurate species-specific geographic area reduction data in the past centuries are scarce for many species, we leveraged existing demography indicators to serve as proxies for habitat transformations and showcase how we can bridge these with population genetic theory to achieve estimates of genetic diversity loss.

The first prediction uses species assessment documents from the Red List from the International Union for Conservation of Nature (IUCN), which contains habitat area and population size decline information within ±10 years / ±3 generations, especially under classification criteria A1-A4c and C1^8,34,35^ (while other criteria are also relevant for extinction risk due to small ranges and populations, such as B1–2 and D, they rely on fixed thresholds, such as fewer than 1,000 individuals, without considering trends over time.). We must emphasize that we do not expect genetic diversity losses to only apply to threatened species where population sizes are close to the typical ${\text{N}}_{\text{e}}$ 500 limit used in conservation genetics (a minority of species 1,800 out of 82,798 have less than N<1000 census individuals; Red List criterion D, Table S16, while the majority of genetic biodiversity losses is attributed to many thousands of non-threatened yet declining species, ~65% of Red List species are least concerned or near threatened, but 65% of those have a demographic decline). Hence, we attempt to translate assessments of all 82,798 plant, animal, and fungi species of estimated threat categories to genetic diversity loss (Fig. S21, Table S16). For instance, focusing on A2–4c criteria only: there were 2,240 *vulnerable* species which suffered ≥30% area loss $(\left.1-A_{\text{present}}/A_{\text{past}}\right)$, 1,621 endangered species that lost ≥50% of area, 916 critically endangered species that lost ≥80% of area, and 1,688 that did not pass the ≥25% area loss threshold that have thus been classified as least concern (other criteria that contain area information can also be used, Fig. S21, Table S16). Using MAR/GDAR, we transform these area loss ranges into expected genetic diversity loss. In cases where genetic data (${\text{F}}_{\text{ST}}$, $z_{MAR}$, $z_{\text{GDAR}}$) is not available, we utilize ranges from species with empirical data: $F_{ST}=0.01-0.6$, $z_{MAR}=0.01-0.8$, and $z_{\text{GDAR}}=0.01-0.8$ (Fig. 4A, Fig. 4B–E, Table S16). One such species of least concern, *Eucalyptus melliodora*, is a common tree of Australia for which we have genomic data (Fig. 4A, S19, Table S15). It was assessed as vulnerable under criterion A2c, meaning its area loss must exceed 30% but remain below 50%; otherwise, it would be classified as endangered. With this, its minimum expected allelic genetic diversity loss using MAR is equivalent to: *1-(0.3)^0.3^ = 10%;* and a maximum of *1-(0.5)^0.3^ = 19%;* (midpoint reported in Fig. 4A). The GDAR approach can be applied to predict nucleotide genetic diversity loss (π) by substituting $z_{\text{GDAR}}$ values in the same MAR equation (Table S15). Equivalently, we can use the species’s average $F_{ST}$ along with our WFmoments pre-computed loss tables (github.com/moiexpositoalonsolab/mar2). For *E. melliodora*, a $F_{ST}=0.01$ and area loss of 30–50%, would incur in a short-term nucleotide diversity loss of 0.15% (IQR=0.11–0.18%) and a long-term loss of 39.5% (IQR=34.5–44.5%). Applying these approaches across 7,263 species evaluated in the Red List with A1–4 criteria, we found a GDAR/*WFmoments*-based average short-term nucleotide genetic diversity loss of 12.8% (IQR=0–17.3%), and MAR-based allelic richness loss of 28.8% (IQR=1.4–44.1%) (Fig. 4B). Assuming no further habitat losses in the 21^st^ century, we predict using WFmoments that the lagged dynamics of genetic diversity will continue increasing losses to a long-term nucleotide diversity average of 41.3% (IQR=12.8–64.1%) (Fig. 4C).

For the second predictions, we used the comprehensive demographic survey data across 5,579 threatened and threatened and non-threatened vertebrate species from the Living Planet Index (LPI)^36^, to compute population decline losses per species, with a specific focus on within-species average census losses: 70% (IQR=51–93%) (Fig. S16, Fig. S17, see others in **Materials and Methods**). As before, using GDAR/MAR, we estimate that across 4 decades of data there is an average population size decline per species that corresponds to a nucleotide genetic diversity loss (π) on average 15.7% (IQR=0–16.3%) (Fig. 4B, Fig. S18) while average allelic richness loss is 38.7% (IQR=11.5–60%) (Fig. 4B). Long-term projections with *WFmoments* estimate 48% nucleotide diversity loss (IQR=29–69%) (Fig. 4B, Fig. S18).

Third, the GEOBON Genetic Composition group showcased the feasibility of 2022’s GBF genetic proxy indicators by carefully annotating 966 species for 9 countries^37^. The fraction of wild populations lost (Indicator 2) per species averaged 17.6% (IQR=0–33%); of which 73.3% (IQR=50–100%) of the populations had over 500 ${\text{N}}_{\text{e}}$ (Indicator 1) (note that ${\text{N}}_{\text{e}}$ is assumed^38^ to be 10% of individual census size ${\text{N}}_{\text{c}}$, although there are discrepancies on how variable ${\text{N}}_{\text{e}}/{\text{N}}_{\text{c}}$ ratios can be from one^39^ to several orders of magnitude across vertebrate species^17^). As before, using population loss indicator 2 across species in combination with the MAR/GDAR/WFmoments framework, we estimated an average nucleotide diversity loss of 1.5% (IQR=0%) and allelic richness loss of 7.2% (IQR=0–7.5%) in the short-term, and 6% (IQR=1–2%) nucleotide diversity loss in the long term. For now, it is unclear how indicator 1 may be used for population genetic predictions, as ${\text{N}}_{\text{e}}$ within a population not only depends on ${\text{N}}_{\text{c}}$ but also on gene flow, migration and connectivity between populations (e.g. at equal ${\text{N}}_{\text{c}}$, increased gene flow increases local ${\text{N}}_{\text{e}}$). In the absence of a complementary quantitative demographic value, this qualitative indicator is helpful in prioritizing efforts and detecting imminent population and genetic diversity losses^38^. For instance, combining indicator 1 and 2, we may anticipate that the 26.6% of populations which are below ${\text{N}}_{\text{e}}<500$ will likely become extinct soon. Adding this to the already lost 17.6% of populations per species would result in a 39% of total populations (soon-to-be) lost, a mean nucleotide diversity loss of 22% (IQR=0–16%) and an allelic richness loss of 40% (IQR=1–100%).

All above predictions are based on the robust edge contraction patterns (Fig. 2), but we expect fragmentation and other processes to limit genetic diversity loss predictability in certain scenarios. To explore these challenges, we also conducted predictions assuming that monitored species lost populations with high fragmentation. Because we lack quantitative connectivity metrics of populations evaluated in LPI, Red List, and GEOBON-driven GBF indicators, we utilized *WFmoments* and assumed the high random fragmentation and isolation levels of our simulations (Fig. 1, 3). Since we observed that in fragmented landscapes within-population or local genetic diversity may be most diagnostic (Fig. 3), we summarized predictions of within-population genetic diversity (Fig. 4D–E). These show a 2% nucleotide diversity loss (IQR=0–3.9%) in the short term and a 71% nucleotide diversity loss (IQR=49–95%) in the long term. All in all, even under complex fragmentation scenarios, across prediction approaches and diversity metrics, we expect to have already lost moderate levels of genetic diversity. These genetic diversity losses will become more dramatic over the next decades even if no more populations or habitats are lost.

These first estimates of present and future global genetic diversity lost across thousands of species solidify our previous warnings of major genetic biodiversity declines in the Anthropocene^8^. On average, species have likely already surpassed the proposed threshold of “90% of genetic diversity within all species,” a target initially suggested by the United Nations for inclusion in the GBF but later withdrawn^40^. To achieve the new broad GBF target of “maintain[ing] and restore[ing] the genetic diversity within and between populations” (CBD/COP/DEC/15/4), we need realistic and data- and theoretically-sound goals, which requires recognizing several biological principles of spatiotemporal dynamics of genetic diversity. Most species are distributed across multiple populations, with population sizes likely well above an effective population size $\left({\text{N}}_{\text{e}}\right)$ of 500. Consequently, the loss of genetic diversity across species and ecosystems will largely result from population- and habitat reductions, impacting all species, including a large majority of non-yet-endangered species. In addition, the lag dynamics of genetic diversity with demographic changes means that most genetic diversity loss will not occur immediately with habitat loss, but rather over the next decades or centuries, even if we protect all species and habitats. These new insights signal an urgent need for action and provide both a tool and a window of opportunity to ambitiously recover and reconnect populations, ensuring species’ long-term genetic protection.

## Methods summary

### Continuous-space population genomic simulations with SLiM

To study genetic diversity under different scenarios of species ranges and extinctions, we set up forward simulations in continuous 2D space using SLiM v. 4.0.1^26^. We simulated diploid genomes using a single, 10^8^ long chromosome, with recombination rate and mutation rate set at 10^−8^. Utilizing SLiM’s non-Wright-Fisher mode, we simulate a single population, the size of which is maintained through density dependent fitness effects given a carrying capacity K, which limits the census of individuals in the landscape, ${\text{N}}_{\text{c}}$. Spatial structure is established by associating each individual with a continuous 2D coordinate (i.e. latitude and longitude), and by using these coordinates to govern three demographic processes: mate choice, dispersal, and competition. For mate choice and reproduction, if the focal individual is of fertile age, a mate is chosen randomly and weighted by spatial proximity, to generate a number of offspring sampled from a poisson distribution parameterized to control average fertility. A newly generated offspring’s position is drawn from a Gaussian distribution centered at the location of the maternal individual with a standard deviation of dispersal rate. In addition to this juvenile dispersal, all individuals alive at the end of a simulation tick disperse randomly, using twice the juvenile dispersal rate. The effect of spatial competition is based on the local population density felt by each individual. This is established using a Gaussian interaction to govern the strength of spatial competition, which is then rescaled based on the carrying capacity to keep the population at a size parameterized by the carrying capacity. During habitat reduction, we scale the carrying capacity by a function of habitat size to ensure that a reduction of habitat by 50% also leads to a 50% reduction in carrying capacity of the entire habitat. In addition to the density-dependent fitness effect, we also scale an individual’s fitness non-linearly with its age, up to a maximum age of 10 ticks. Together with a fertile age of 3 ticks, and a possion fertility rate of 0.5, these parameters set up the age structure of our population. Finally, note that the effective population size in population genetics, ${\text{N}}_{\text{e}}$, is not a set parameter but an emergent parameter that may differ from census size ${\text{N}}_{\text{c}}$ in non-panmictic, spatially-structured populations with complex age structures, reproduction, and overlapping generations (all complexities expected in nature). Unless otherwise specified, our simulations utilize a K = 5000 (i.e. carrying capacity ${\text{N}}_{\text{c}}$) and a dispersal rate of 0.05.

To allow time for spatial population structure to develop, we allow this population to evolve forward in time for 1,000,000 SLiM ticks (~200,000 generations) (Fig. S20). As predicted by the isolation by distance pattern, individuals sampled closely together in 2D space are now more genetically related than individuals sampled far apart. Rather than simulating every mutation, which is a major computational burden, we use tree sequence recording ^41^ to track the full genealogy of all individuals in the simulation which are either alive at the end of the simulation or sampled through time using the treeSeqRememberIndividuals function of SLiM. For the purposes of our simulation, we simulate 1,000,000 SLiM ticks (~200,000 generations) to establish spatial structure and ensure that all sampled individuals fully coalesce (in which case the value of genetic diversity resulting from these simulations would be at equilibrium) (Fig. S20). To be able to start simulations with a large population in space that is at equilibrium without wasting computational resources, we simulated coalescence backwards in time with msprime ^41^. This process has been referred to as “recapitation” ^42^, where an incomplete genealogy of a large population with multiple roots (from SLiM) is “recapitated” using coalescent simulation backwards in time. This is made possible by using the tree sequence data structure to record and simulate genealogies in both SLiM and msprime. Since our simulation is only concerned with how processes such as dispersal affect neutral variation across space and through time, we can use the “recapitated” tree sequence to overlay mutations onto the full genealogy of all sampled individuals. The rationale being that under neutrality, mutations will not affect the structure of the genealogy, so we can simulate the genealogy without mutations first, before overlaying neutral mutations to reduce computational burden. We then extracted the resulting genotypes of all individuals from the tree sequence for downstream analysis. We partitioned the continuous-space simulation landscape in SLiM into a 10×10 grid for subpopulation sampling. For each grid, we calculated the pairwise genetic diversity, both π and number of segregating sites, between individuals in all 100 spatial grids. In addition, we tracked each individual’s location within spatial grids and the total number of segregating sites at each sampling time point. In further downstream analysis, we approximate that each generation corresponds to 4.5 SLiM time points as we simulate realistic age-structured populations with overlapping generations.

### Genetic diversity metrics

Genetic diversity is measured as π, the average pairwise difference between all possible pairs of individuals, and S, allelic richness (or the number of segregating sites). In addition, we proceed to calculate both within-population (one grid cell) and species-wide metrics of both π and S. We measured genetic diversity using pairwise genetic diversity with the equation below, where n is the number of remaining individuals, L is the total number of SNPs selected, and p_i_ is the allele frequency at genomic location i. The percentage of area loss was calculated by dividing the total number of map cells removed with the number of total map cells constructed from all geo-referenced individuals at each iteration. Genetic diversity was estimated as: $\pi =(n/n-1)(1/L)\Sigma_{i}^{L}2p_{i}\left(1-p_{i}\right)$.

#### Within-population genetic diversity metrics

Within-population genetic diversity metrics calculate the average pairwise difference between all possible pairs of individuals ($\pi_{\text{local}}$) within each grid cell across all 100 grid cells. In total, we obtain 100 values of π or S, and proceed to calculate the average of π or allelic richness across all 100 grid cells. This ensures that individuals that belong in the same population are compared with each other.

#### Species-wide genetic diversity metrics

Species-wide metrics ($\pi_{\text{species}}$) are calculated by getting the average pairwise difference between all possible pairs of individuals within all grids, so individuals-possibly in separate grids-are compared to each other to obtain an average value for all 100 grid cells.

### Simulating different scenarios of human impacts on species habitats

Our simulations included two ways of habitat destruction, habitat loss from one leading edge, and habitat loss with fragmentation.

#### Instantaneous range contraction simulations

To assess the dynamics of genetic diversity in populations undergoing habitat loss, we performed range contraction simulations starting from 10% habitat loss to 90% habitat loss at 10% increments, with 9 replicates at each progression of habitat loss. Each simulation was run for 1,000,000 SLiM time points before instantaneous habitat loss occurred at 1,000,001 SLiM time points. After which, we tracked the dynamics of both π and allelic richness in the short-term (at 1,000,001 SLiM time point after habitat loss) and in the long-term (at 1,062,001 SLiM time points or approximately 13,800 generations after habitat loss). Unless otherwise specified, we utilized a census population size of ${\text{N}}_{\text{c}}=5,000$ and a dispersal rate of 0.05.

Under habitat loss with fragmentation, we performed habitat loss simulations from 6% to 93%. We utilized a random integer generator to select which grids in our 10×10 simulation map to “extinct”, hence causing the range of habitat loss percentages to be variable. Each simulation run generates a unique habitat fragmentation map. In total, we simulated 121 different habitat loss maps with fragmentation. Unless otherwise noted, we utilize a 10×10 simulation map for our simulations.

In order to briefly explore other habitat fragmentation scenarios, we performed habitat loss simulations on a larger geographical grid of 20×20. Utilizing a random integer generator to select grids in our 20×20 simulation map to “extinct”, this simulation setup ensures that habitat loss is distributed more evenly across the geographical grid and at smaller areas than previously implemented. We term this as “mini” fragmentation to understand how genetic diversity changes with time if fragmentation occurs at much smaller scales across a wider geographical range.

#### Altering parameters in spatial population dynamics

Given the expectation that key population genetic parameters affect the dynamics of how genetic diversity changes with habitat loss, we wanted to explore how population size and migration rate might affect π diversity estimates under various habitat loss scenarios. We utilized population sizes ranging from 500 to 10,000 (exceptionally 20,000 individuals for computational efficiency) and varied dispersal rate respectively to test our variable Fst values to mimic low, medium and high population structure. Specifics of combinations of population sizes and dispersal rates can be found listed in the legend of Fig S1.

#### Gradual habitat loss simulations

To explore alternative habitat loss scenarios, we also looked at gradual habitat loss dynamics where habitat loss would decrease by 1% every 11 generations. This simulation was run for 2,000 SLiM time points before gradual habitat extinction of 50% habitat loss which occurred from 2,001 SLiM timepoints (approximately 450 generations) to 2,250 SLiM timepoints (500 generations).

### Fitting mathematical theory to simulations

Our modeling (see Mathematical Appendix) has three free parameters: effective population size, mutation rate, and migration rate. Under standard Wright-Fisher dynamics these may all be derived from real-world observable data, namely the census population size, rate of new mutations per generation, and number of migrants per generation. Yet, our simulations contain a number of features that strongly deviate from the standard Wright-Fisher model. There are overlapping generations, individuals live in continuous space instead of discrete demes, and there is density-dependent selection and spatially controlled mate choice. The Wright-Fisher model is surprisingly robust to deviations from its assumptions with the caveat that parameters must now be interpreted in terms of an “effective population size” that will depend on the actual model in complex ways ^43^. As a result, we expect our mathematical model to be able to recapitulate key features of the simulation, albeit with modified parameters.

To find the parameters that provided a good fit between our theory and our simulations we used least-squares fitting. We approximated the continuous habitat in the simulations by a 10 by 10 grid of square demes, with migration between adjacent demes. We then fixed the effective population size and optimized the least-squares fit between the theoretically predicted species-wide π from simulations and theory over three parameters: the mutation rate, the migration rate between adjacent demes, and a time-scaling for converting units of time in our theoretical model to SLiM timepoints in the simulations. Optimization was performed using the “Powell” method in scipy.optimize ^44–46^.

### Reanalyses of population genomic datasets of 18 species

#### Generating F_st_ values across diverse species

We utilized datasets across diverse species that were collected for^8^. All datasets were transformed into PLINK files using PLINK v1.9 ^47^. For additional information on data processing, refer to the Supplementary materials of^8^. For computational efficiency, we generated F_st_ values using admixture v1.3.0 ^48^, specifically utilizing the command àdmixture –cv sample.bed <K or number-of-clusters>` where we tested a range of 1 to 15 K and picked the K with the lowest CV error. We reported the average and maximum F_st_ values across K populations for each species (Table S15).

#### Simulating short-term extinction in empirical datasets

To confirm the genetic extinction patterns we observed in population genomic simulations and mathematical models, we also simulated short-term extinctions in empirical sequencing datasets of nineteen wild plant and animal species (detailed description of datasets available in Exposito-Alonso et al. 2022). Briefly, for each species, the empirical dataset contains geo-referenced individuals broadly sampled across its geographical distribution and naturally occurring mutations were discovered through various DNA sequencing methods. We conducted the analyses with up to 10,000 randomly selected biallelic SNPs for each species sampled genome-wide, or in the largest chromosome for those species with large genomes. We sought to use unfiltered SNP datasets to avoid ascertainment biases.

For each species, the *in silico* extinction simulations were conducted by iteratively removing map cells in the sample map, and geo-referenced individuals falling within the removed cells are considered extinct. Genetic diversity was estimated from the genotype matrix of remaining individuals using the R package MAR v0.0.3. We measured genetic diversity using pairwise genetic diversity with the equation below, where n is the number of remaining individuals, L is the total number of SNPs selected, and p_i_ is the allele frequency at genomic location i. The percentage of area loss was calculated by dividing the total number of map cells removed with the number of total map cells constructed from all geo-referenced individuals at each iteration. Genetic diversity was estimated as: $\pi =(n/n-1)(1/L)\Sigma_{i}^{L}2p_{i}\left(1-p_{i}\right)$.

We implemented two hypothesized patterns of extinction to match the population genomic simulations presented in the main text (Table S5, Table S6). The random scheme involved extinctions occurring randomly across the range as would be expected by the habitat fragmentation scenario, while the south-north scheme had extinctions starting in the north and moving southward simulating the impacts of climate change. Each species underwent 20 replicates for both the random and south-north extinction schemes.

### Estimating global genetic diversity loss based on global population and area loss census

#### Living Planet Index

In order to estimate global genetic diversity loss, we utilized population sizes of 5,230 species that have been tracked for over 3 decades via the Living Planet Index^36^. Utilizing this dataset, we approximated average population declines in a couple of ways. For each species, we summarized the population data in a couple of ways. To obtain the fraction of populations lost, we obtained the mean, arithmetic mean and geometric mean of populations that have reached 0 over 3 decades. We then utilize these values as a proxy for habitat loss. For more information on the distribution of LPI data used, see Fig. S16, 17.

We assumed that F_st_ across LPI species would follow a normal distribution with mean 0.270 and standard deviation 0.211 (as determined by the F_st_ values we obtained across diverse species, see Table S15). Utilizing these F_st_ and habitat loss approximations, we estimated the amount of short- and long-term genetic diversity utilizing our theoretical framework. To obtain an average global genetic diversity loss estimate, we summed the total genetic diversity loss to obtain the total amount of genetic diversity loss divided by all populations considered.

#### Red list

We obtained criteria of area loss from the Red List database (www.iucnredlist.org). Counts of each category and criteria used in Red List classification are summarized (see Supplementary data). Genetic diversity loss values were calculated for all Red List categories: Extinct, Likely Extinct, Critically endangered, Endangered, Vulnerable, Near Threatened and Least Concern. We approximated area loss by using population size loss criteria for each of the 6 categories by taking the arithmetic mean of the minimum and maximum of the range. For example, a species is categorized as critically endangered when population size loss is between 80 to 95%. Utilizing these approximated area loss values for each category, we then used our theoretical and simulation based framework to make predictions of short and long-term genetic diversity π (see Supplementary data).

#### New KM GBF proxy indicators for genetic diversity

We obtained Indicator 2 calculations from ^49^. We calculated Indicator 2 using data from the Living Planet Index (see Supplementary data for details, Fig. S22). To obtain the fraction of populations lost in the LPI, we utilized the mean of populations that have reached 0 over 3 decades.

## Supplementary Material

1

## Figures and Tables

**Fig. 1 |:**
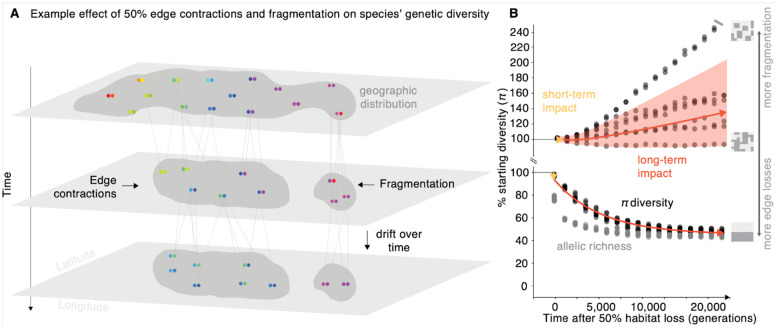
Genetic diversity metrics after habitat destruction (**A**) Cartoon depicting a geographic distribution of a diploid species with spatial genetic structure (colors), the genealogy of genotypes, and how genetic diversity changes after area or population losses through edge contraction and fragmentation. (**B**) Genetic diversity as average pairwise nucleotide distances (π) and richness of alleles or mutations (M), trajectories after geographic range loss driven by habitat fragmentation over time. Each trajectory represents a replicate for 50% habitat loss over time, where each replicate is a different habitat fragmentation map. Habitat fragmentation maps vary according to level of connectivity between patches of landscape. Each black dot represents a sampled time point of π for that trajectory. Trajectories were tracked across 20,000 generations. Red arrow represents average long-term genetic diversity projection based on our theoretical framework, with shaded areas showing 95% confidence intervals. Yellow arrow represents average short-term genetic diversity projections based on our theoretical framework. (Species parameters: ${\text{F}}_{\text{ST}}=0.3$ and $\theta =10^{-4}$)

**Fig. 2|:**
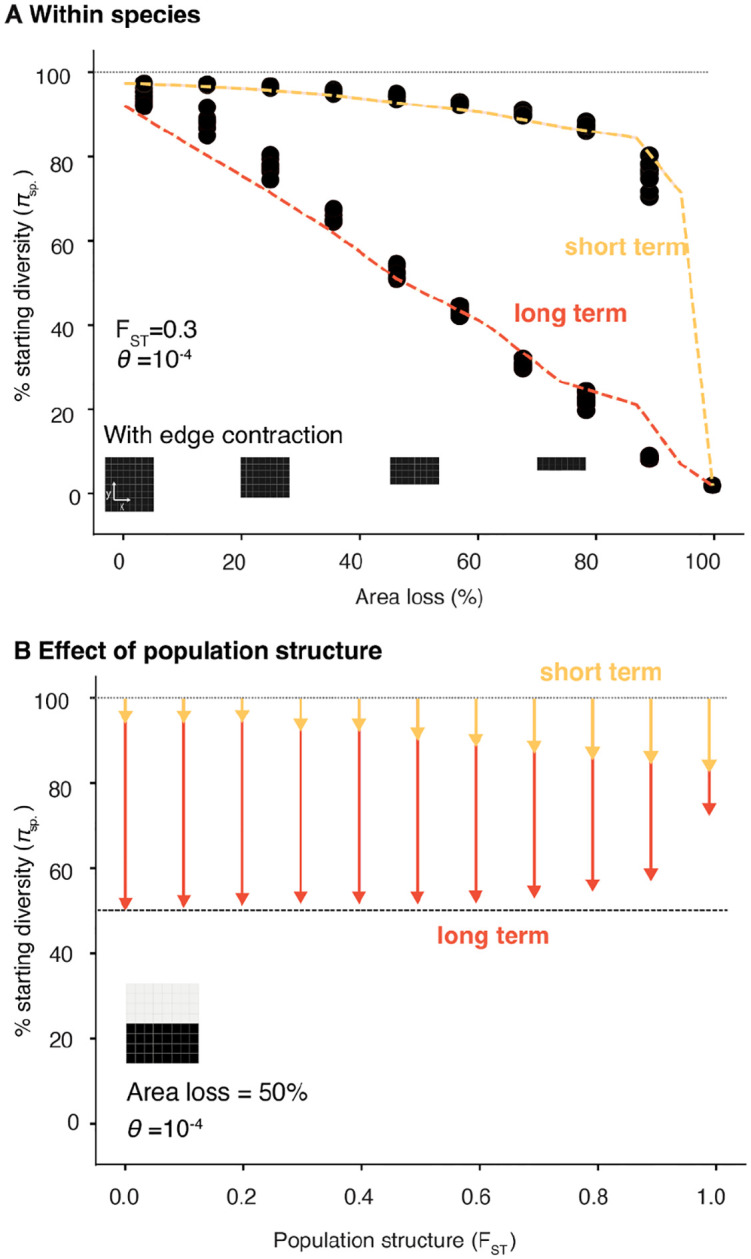
Predictable short- and long-term genetic diversity losses from edge contraction (**A**) Landscape simulation (n=9 for each 10 area losses) of genetic diversity and theoretical expectations (lines) immediately after habitat loss (short-term, yellow) and at final equilibrium (long-term, red). (**B**) The dependence of short-(yellow) and long-term (red) losses with geographic population structure defined by average pairwise FST across 100 subpopulations (10 × 10 grid).

**Fig. 3 |:**
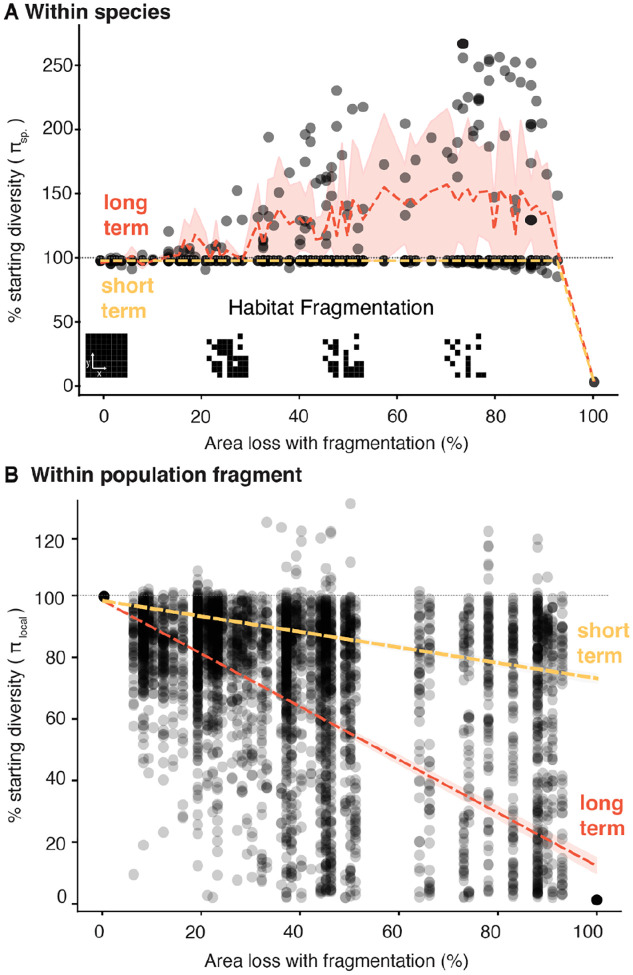
Impacts from habitat destruction by fragmentation are only detectable in within-population genetic diversity (**A**) Overlay of theoretical and simulation-based projections of short- and long-term genetic diversity loss (π) due to habitat loss with fragmentation (121 random fragmentation maps). Each dot represents an average measure of π across a landscape per simulation and overlaid the walking average and 95% interval of short (yellow) and long-term (red) genetic diversity. (**B**) Within-population genetic diversity, $\pi_{\text{local}}$, for the samesimulations as (A). Dotted lines represent a linear model.

**Fig. 4 ∣:**
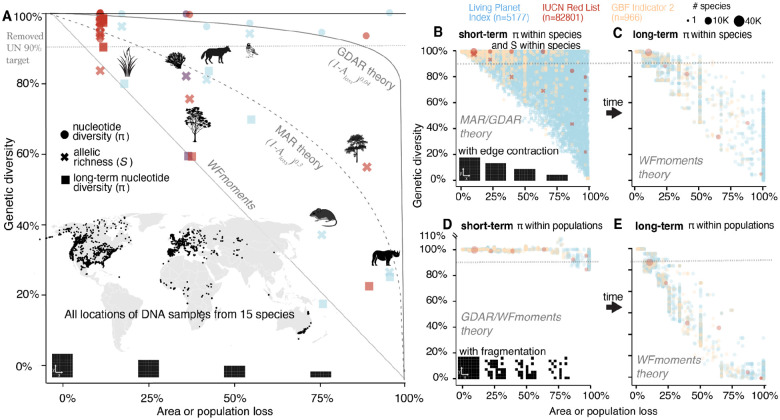
Estimates of genetic diversity loss for empirical, LPI and Red List species over both the short and long-term (**A**) Estimates of genetic diversity loss for 18 plant and animal species for which genomic information is also available to calibrate MAR/GDAR/*WFmoments* predictions. Using per species $z_{\text{MAR}}$ parameter and the MAR equation, we predict short-term allelic richness (crosses) losses. Using per species $F_{ST}$ or $z_{\text{GDAR}}$ and GDAR and *WFmoments*, we predict short-term nucleotide genetic diversity losses (π, dots). Finally, using *WFmoments* we also predict long-term nucleotide genetic diversity losses (π, squares). Area or population losses per species are extracted from Red List category (red), Living Planet Index (blue), or additional expert range (purple). Predictions follow the edge contraction expectations. Dotted line at 90% represents the now-removed preliminary UN genetic diversity target of 90%. (**B**) Genetic diversity loss projections for 13,808 species for which recent area or population loss information is available from Red List (red), LPI (blue), or new GBF indicators (orange). Predictions of allelic richness (crosses) with MAR use randomly sampled $z_{MAR}$ parameters for 13,808 species following distribution of known species in (A), and predictions of nucleotide diversity π (dots) with *WFmoments* follow the same approach sampling $F_{ST}$. Sizes of dots represent numbers of species, as in Red List many species have the same threatened category and there are no species-specific area losses. (**C**) Long term projections of genetic diversity loss following the short-term reduction in (B) assuming area and population losses stop. (**D-E**) Genetic diversity projections for the same 13,808 species as in (B) assuming area loss with high fragmentation and isolation using *WFmoments* in the short-term (D) and long-term (E).

## Data Availability

Scripts will be deposited publicly at https://github.com/moiexpositoalonsolab/mar2. WFmoments is available at https://github.com/jeffspence/wfmoments.
