# Supplementary material for: Genetic diversity loss in the Anthropocene will continue long after habitat destruction ends
Source: bioRxiv. 2024 Oct 22:2024.10.21.619096. Preprint. [Version 1] doi: 10.1101/2024.10.21.619096 (PMC11526952; doi:10.1101/2024.10.21.619096)
Supplement: Supplementary file 1 [file NIHPP2024.10.21.619096V1-supplement-1.pdf]

bioRxiv preprint doi: https://doi.org/10.1101/2024.10.21.619096; this version posted October 22, 2024. The copyright holder for this preprint (which was not certified by peer review) is the author/funder, who has granted bioRxiv a license to display the preprint in perpetuity. It is made available under aCC-BY 4.0 International license.

Supplemental Materials for:

Genetic diversity loss continues long after habitat destruction ends

Kristy S. Mualim[1,2,3‡], Jeffrey P. Spence[4‡], Clemens Weiß[4,5‡], Oliver Selmoni[1,3], Meixi Lin[1,3] , Moises Exposito-Alonso[1,2,3,6,7*]

[1]Department of Plant Biology, Carnegie Institution for Science, Stanford, California, USA
[2]Department of Biology, Stanford University, Stanford, California, USA
[3]Department of Integrative Biology, University of California Berkeley, Berkeley, USA
[4]Department of Genetics, Stanford University, Stanford, California, USA
[5]Stanford Cancer Institute, Stanford University, Stanford, California, USA
[6]Department of Global Ecology, Carnegie Institution for Science, Stanford, California, USA
[7]Howard Hughes Medical Institute, University of California Berkeley, Berkeley, USA
[‡]Authors contributed equally
[*]Correspondence: moisesexpositoalonso@gmail.com

Supplementary Text

Text S1-S5

Supplementary Figures

Figs. S1-S128

Supplementary Tables

Figs. S1-S17

Mathematical Appendix

bioRxiv preprint doi: https://doi.org/10.1101/2024.10.21.619096; this version posted October 22, 2024. The copyright holder for this preprint (which was not certified by peer review) is the author/funder, who has granted bioRxiv a license to display the preprint in perpetuity. It is made available under aCC-BY 4.0 International license.

Supplementary Text

Text S1 | Simulation model assumptions and limitations

Our simulation-based predictions make a series of assumptions such as: (1) species are at equilibrium prior to any habitat loss; and (2) no further habitat loss occurs after the initial reduction. These projections will likely change when considering fluctuating population sizes, further changes in habitat, or changing migration patterns **(Fig. S3, Fig. S24, Fig. S25)**. Prior to altering the habitat of a species, we ensured that the species was at equilibrium by allowing the simulations to run for a defined burn-in rate of 1,000,000 SLiM timepoints. This was done to ensure that π was at a stable equilibrium before range contraction occurred **(Fig. S23)**.

Species at equilibrium

Initially, we performed simulations of edge contraction without sufficient burn-in (species are not at equilibrium), which caused genetic diversity trajectories across different percentages of habitat loss to become highly variable in the long-term **(Fig. S24)**. This is likely going to be more reflective of how existing populations are in the wild and hence, further experiments should be performed to calculate how the dynamics of genetic diversity changes for a species not at equilibrium under habitat loss.

Constant population size

To address the second assumption of our model, we only induce just one bottleneck event (habitat range loss) before allowing the population to reach equilibrium over time. We assume that long-term constant population size from the time of habitat loss to the next equilibrium. This is designed for us to understand how habitat loss impacts genetic diversity immediately after habitat loss as well as across generations. However, it is likely that species in the wild will encounter frequent bottleneck events which would cause these genetic diversity trajectories to vary from those predicted in this work. We recommend further simulations in understanding how repeated bottleneck events may impact genetic diversity trajectories in the wild. It's likely that these projections will become increasingly complicated, such as those seen in under habitat fragmentation **(Fig 2)**.

Neutral genetic diversity is heavily dependent on both mutation rate and population size and under the assumption of an infinite sites model in a Wright-Fisher (WF) model, it can be expressed as:

$$E[\pi] = 4N_e\mu \qquad (1)$$

In theory, N_e can be understood as a key population genetic parameter that determines the number of breeding individuals in an idealized population that shows the same level of genetic drift or inbreeding as the observed population. N_e affects the rate of loss of genetic diversity and inbreeding and is an emergent property in our simulations. Accordingly, across generations, we expect that contemporary N_e will fluctuate according to the number and variance of offspring per generation, as well as the number of potential parents that can pass on genetic material to the next generation.

Neutral genetic diversity

bioRxiv preprint doi: https://doi.org/10.1101/2024.10.21.619096; this version posted October 22, 2024. The copyright holder for this preprint (which was not certified by peer review) is the author/funder, who has granted bioRxiv a license to display the preprint in perpetuity. It is made available under aCC-BY 4.0 International license.

The field of conservation genetics often aims at preserving genetic diversity to prevent inbreeding and promote evolutionary adaptation—however this is practically achieved through presumably-neutral genetic diversity as this represents the bulk of genetic diversity and serves as a proxy for adaptive/deleterious variation [27]. Our current theoretical and simulation projections also do not incorporate selection on adaptive and deleterious mutations.

In addition, our framework also assumes that population sizes are large enough to be modeled across time. It is likely that once we consider extremely small populations, which has typically been the focus on conservation genetics geared towards endangered species, processes like mutational meltdown or inbreeding effects may take hold sooner than equilibrium is achieved. This is because smaller populations may suffer from increasing mutation load which in turn, causes the step-wise successive loss of individuals with high fitness due to both mutation accumulation and genetic drift (termed Muller's ratchet).

More work needs to be done to understand how genetic diversity projections will change with selection and adaptation under habitat loss scenarios and how these processes may impact modeling for species with small population sizes. There are many possibilities in modeling these and are thus beyond the scope of this manuscript, where our focus is understanding neutral genetic diversity loss in spatiotemporal non-equilibrium dynamics.

Text S2 | Genetic diversity dynamics for geographic habitats of 1D and 2D

In most of the main text, we only considered two-dimensional square landscapes. Yet, alternative habitat geometries are common across species. Utilizing our theoretical framework, we explore the implications of genetic diversity across 1-D habitats and compare it to the 2-D habitat that we study in the main text.

We find that short-term genetic diversity loss for 1-D habitats is more severe than 2-D habitats with increasing habitat area loss **(Fig. S25)**. This relationship is exacerbated when the migration rate is low **(Fig. S25)** and expectedly, becomes more dramatic with increasing habitat loss. This means that species that have low migration and a 1-D habitat range, or a habitat range that can be characterized as long and narrow, lose much more genetic diversity during habitat loss. This loss can be up to 3 orders of magnitude worse than that of species that also have low migration but a 2-D habitat range, or a habitat range that can be characterized as both long and wide. In the long-term, genetic diversity loss for 1-D habitats is also more severe than 2-D habitats with increasing habitat area but this behavior is more exaggerated under low migration regimes **(Fig. S25)**. Under high migration regimes, habitat geometry matters less and genetic diversity loss for both 1-D and 2-D habitats are similar **(Fig. S25)**.

To explain the differences between the 1-D and 2-D results, we consider a decomposition of species-wide π as a combination of the average π within each deme (π_{within}), and the variance of allele frequencies across demes ($d_{between}$):

$$\pi_{total} = \pi_{within} + d_{between} \qquad \text{(equation 1)}$$

The first term measures diversity within each deme and refers to the average genetic diversity observed within individual subpopulations while the second term measures divergence across demes and measures how different allele frequencies are from one deme to another. This second term will be larger the less migration there is between pairs of demes.

Habitat loss affects each of these two terms differently. Intuitively, π_{within} can be thought of as being related to the effective number of ancestors a randomly chosen individual has. If an individual is in a deme that has many neighbors that are connected by high migration rates, then that individual could have ancestors from any of these nearby demes, resulting in a higher diversity within that deme. Conversely, if an individual is from an isolated deme, their ancestors must all come from just that deme, resulting in lower within-deme diversity. As a result, higher migration rates result in larger within-deme diversity. On the other hand, $d_{between}$ can be thought of as how difficult it is to migrate from one deme to another on average. The more connected demes are by migration, the more similar their allele frequencies will be and the smaller $d_{between}$ will be. As a result increasing migration results in smaller $d_{between}$.

bioRxiv preprint doi: https://doi.org/10.1101/2024.10.21.619096; this version posted October 22, 2024. The copyright holder for this preprint (which was not certified by peer review) is the author/funder, who has granted bioRxiv a license to display the preprint in perpetuity. It is made available under aCC-BY 4.0 International license.

The geometry of the habitat affects how habitat loss changes π_{within} and $d_{between}$. For example, in a 1-D habitat, losing 90% of the habitable area results in the remaining demes being much closer together, resulting in a much smaller $d_{between}$. In contrast, in a 2-D habitat, even after losing 90% of the habitable area, some pairs of demes remain extremely distant and poorly connected by migration, having only a modest impact on $d_{between}$. As a result, when migration rates are low enough to make $d_{between}$ the dominant contributor to π, habitat loss affects π much more strongly in a 1-D habitat than a 2-D habitat.

Text S3 | A coalescent interpretation of habitat loss

These incredibly large long-term genetic diversity losses are worrisome, but have an intuitive population genetic explanation **(Fig. S13)**. Diversity in population genetics is related to the number of potential ancestors an individual might have [50–52]. If a population of present day individuals has a large pool of potential ancestors, then the population is more diverse than a population with a smaller pool of potential ancestors. Immediately following habitat loss, individuals can still have ancestors from across the entire species range, and hence habitat loss has little immediate effect on π. As time goes on, however, all of an individual's ancestors that lived after the loss of habitat must come from a smaller pool of ancestors living in the habitable area. After enough time has passed, all of the individuals in a population will have a most-recent common ancestor that survived during the habitat loss, and therefore all of their relevant ancestors will have come from the smaller pool of individuals that could live in the reduced range. At this point, π equilibrates to its new value and no further changes should occur.

Text S4 | Genetic diversity-area relationships and power laws

Background on biodiversity SAR and population genetics diversity MAR power laws

Classic population genetic theory has described that as individuals of a species move over generations and accumulate mutations, an isolation by distance pattern emerges [53], whereby the genetic distance between two individuals increases with the geographic distance between them.

From this principle, it is expected that larger areas of populations should harbor larger numbers of mutations. It was not until recently that we formally proposed and described a mutations-area relationship [8] inspired by the well-known ecological relationship species-area relationship [20,54,55]. Apart from the isolation by distance pattern, a suspicion that the mutations-area relationship could exist specifically as a power law equation came from the observations that species relative abundances in an ecosystem are very uneven, with the majority of species being at low frequencies called the “commonness of rarity”. This is identical to the concept of the site or mutation frequency spectrum, whereby the majority of mutations remain at low frequency. It has also been pointed out that a number of ecological and evolutionary principles are analogous: speciation is equivalent to mutation, ecological drift is equivalent to genetic drift, environmental species filtering is equivalent to natural selection [56]. From the assumption of “commonness of rarity” of species abundances following a log-Normal distribution, Preston analytically derived a power law of species-area relationship with the rationale behind that as area of ecosystems or islands increase one would encounter more new, rare, species.

The species-area relationship (SAR) power law then follows: $S=cA^z$; where species is S and area is A, and the scaling coefficient z describes the spatial structure of species in geographic space with a constant c. Preston derived theoretically the scaling should be z=0.27, under a number of assumptions (Fig. S3). This has been empirically shown to be close to reality [57], although there is some variation across ecosystems and spatial scales with causes and implications widely discussed although still debated [54].

bioRxiv preprint doi: https://doi.org/10.1101/2024.10.21.619096; this version posted October 22, 2024. The copyright holder for this preprint (which was not certified by peer review) is the author/funder, who has granted bioRxiv a license to display the preprint in perpetuity. It is made available under aCC-BY 4.0 International license.

The equivalent of species numbers of richness in genetics is the allelic richness, or segregating sites, or plainly number of mutations. We hence proposed a mutations-area relationship (MAR) of the same form: $M=cA^z$; with z_{MAR} (to distinguish from the z_{SAR}). The first empirical tests of such relationships over 10 thousand genomes of 20 plant and animal species showed an intriguing average scaling per species of $z_{MAR} = 0.3$ [8].

We are yet to analytically derive an exact expectation of a z value given species traits, but the boundaries of the $z_{MAR} \subset (0 - 1]$ parameters are straightforward . From classic population genetics, we can derive the expectation under no structure, a panmictic population grows its number of segregation sites proportional to the number of individuals as: $M \sim log(N) \sim N^{z \to 0}$. In the opposite scenario, a species whose populations accumulate totally independent new mutations, under an infinite genomic sites assumption, would grow its number of segregating sites exactly proportional to area: $M \sim A^1$.

Power law to predict diversity extinction fractions with area

An appeal of defining within-species genetic variation with a MAR power law is for the widespread use of SAR power law for conservation of species richness [58] including Intergovernmental Panel for Biodiversity and Ecosystem Services (IPBES) [59]. Habitat area loss is the number one threat of species to extinction and much conservation management is area based. For instance, IPBES reports that across countries we have already destroyed or altered ~50% of Earth's terrestrial habitats. The rationale of the power law approach for predictions is that under the original intact conditions (past) the diversity (alleles for MAR, species for SAR) is given by the past area: cA^z_{past} ; and with a contemporary altered landscape with a reduction (a) in area: $A_{present} = A_{past} - a$; the new genetic diversity would be: $cA^z_{present}$. Taking the ratio removes the constant c and gives us the proportion of diversity remaining: $(A_{present} / A_{past})^z$. If we just have the area lost: $A_{loss} = 1-(A_{present}/A_{past})$; which is how often conservation organisms report threat on ecosystems or species, we can just rearrange: $(1-A_{loss})^z$; and if we want to express the diversity loss instead of diversity remaining fraction, we can use: $1-(1-A_{loss})^z$. All these slightly rearranged versions of the MAR/SAR equations are equivalent and are very easily deployed by conservation practitioners.

The MAR equation captures spatial genetic structure of species and thus predicts much more substantial loses of genetic diversity compared to classic population genetic expectations of population bottlenecks because in population genetics most often we assume population panmixia (i.e. free gene flow and no population structure). We can come up with an equation on the immediate effect of a bottleneck. Before we show that with no structure, $M \sim log(N)$. Similarly as MAR, the loss of genetic diversity after a population reduction of N_x individuals would be:

$$1-(log(N_{present}) \;/\; log(N_{past}) \;)^z =$$

$$1- (log(N_{past}(1-Nx))/ \; log(N_{past}) =$$

$$1- (log(N_{past}) + log(1-Nx)) \;/\; log(N_{past}) =$$

$$- log(1-N_x) \;/\; log(N_{past})$$

This derivation shows that the loss of allelic richness or mutations is in the scale of $log(1-x)$; which is very slow, as we expected from having derived the trend under panmixia $z_{MAR} \approx 0$.

In the most extreme spatial structure scenario, a MAR with parameter $z_{MAR} \approx 1$, predicts the fraction loss of geographic area directly translates to the same fraction loss of genetic diversity. Luckily most species studied have a moderate z_{MAR} with an average of 0.3 (**Table S16**).

MAR in the long-term

bioRxiv preprint doi: https://doi.org/10.1101/2024.10.21.619096; this version posted October 22, 2024. The copyright holder for this preprint (which was not certified by peer review) is the author/funder, who has granted bioRxiv a license to display the preprint in perpetuity. It is made available under aCC-BY 4.0 International license.

Our MAR framework was developed for short-term genetic diversity loss. That is, if an immediate area reduction of a geographic range of a species happens, how many unique genetic variants were found in that area and are thus gone. However, with time, the original area reduction causes an increased stochasticity within a species population dynamics that incurs in further loss of genetic variants from increased genetic drift.

We could not derive analytically the z_{MAR} of long-term genetic diversity but the current manuscript aimed to address this through SLiM simulations. Comparing increasing area loss % we impose on a simulated species in the landscape with the long-term genetic diversity after running the simulations for hundreds to thousands of generations, we could phenomenologically fit a power law **(Fig S15)** which discovered a worrisome long-term $z_{MAR} \sim 1$.

From mutations-area relationship (MAR) to genetic diversity-area relationship (GDAR)

We proposed MAR to model allelic richness with area, analogous to species richness in SAR. There are of course multiple metrics to measure genetic diversity. The most prevalent one together with allelic richness in nucleotide genetic diversity or average pairwise distance (π), defined as: $(1/L)\ \Sigma_i^L\ 2p_i(1-p_i)$; where L is the total number of genetic variants assessed and p_i is their frequency in the population.

From a theoretical standpoint, it is unclear why nucleotide diversity π would follow a power law with area. In fact, the appeal of π for population genetics is that because its value is more affected by frequency than the number of loci, it is a more robust metric to the number of sampled individuals in a population (and thus also robust to area sampled).

This is also the case for species diversity metrics such as Shannon's diversity or Sipsons' diversity indices. In fact, Simpson's index or evenness: $D=\Sigma^S p_i^2$; for S species in the ecosystem and p_i for their relative frequency. Despite the biogeography and community ecology field has empirically fitted power laws to other diversity metrics such as Simpson's species diversity [54,60], which is related to nucleotide diversity, π

Text S5 | Initial results on habitat restoration and assisted population recovery

Motivated by the plethora of restoration projects occurring globally, we wanted to understand and explore recovery dynamics of genetic diversity after habitat loss. We caveat these results by restating that our theoretical and simulation-based predictions only consider neutral evolution.

Utilizing our existing habitat loss from one leading edge and habitat loss with fragmentation scenarios, we "restored" habitats by restoring carrying capacity of habitats with carrying capacity zero to one **(Fig. S26)**. For example, for a 50% habitat loss, this means that 50 grids within the 10×10 simulation map have a carrying capacity of 0. During restoration, these 50 grids now have a carrying capacity 1. Thereby, allowing individuals in nearby habitable areas to disperse and eventually repopulate these newly rehabitable areas. Here, we wanted to examine if time of restoration mattered and performed habitat restoration at varying numbers of generations after habitat loss (termed 2000 (early) , 10000 (medium) and 20000 (late) generations). We then tracked species-wide genetic diversity metrics over time and across 10, 50, 90% habitat loss to understand if there was a potential tipping point in which genetic diversity cannot be restored **(Fig. S27)**.

Overall, we found that genetic diversity eventually always bounces back to 100% given sufficient time **(Fig. S27)**. This behavior is more pronounced in the habitat loss from one leading edge scenario, where a substantial short-term reduction in genetic diversity is reported at 50% habitat loss before a gradual increase in genetic diversity metrics with time at the point of restoration **(Fig. S27)**.

bioRxiv preprint doi: https://doi.org/10.1101/2024.10.21.619096; this version posted October 22, 2024. The copyright holder for this preprint (which was not certified by peer review) is the author/funder, who has granted bioRxiv a license to display the preprint in perpetuity. It is made available under aCC-BY 4.0 International license.

Given the complicated genetic diversity trajectories of habitat fragmentation scenarios, we observed less reduction in genetic diversity at 50% habitat loss and a slight increase in genetic diversity metrics at the point of restoration **(Fig. S27).** These observations are similar even when we look at within-species metrics **(Fig. S28)**.

Overall, these simulations seem to show an overall optimistic conclusion that as long as habitat restoration occurs, genetic diversity will eventually be restored. However, given that these long-term trajectories span tens of thousands of generations, these optimistic conclusions might prove to be unrealistic, and are likely species specific. It is likely that species with faster generation times will reach long-term genetic diversity loss quicker than species with longer generation times but genetic diversity loss for species with higher generation times may be more easily recoverable, given that genetic diversity restoration is slow. For species with long generation times, it may be impossible to recover their genetic diversity within timescales relevant for conservation policy. In addition, these results will likely change as one considers the addition of adaptive and deleterious mutations. Hence, more work needs to be done to develop the theory and predictions that would follow habitat destruction and corresponding habitat restoration projects.

Supplemental References

41. Kelleher, J., Etheridge, A. M. & McVean, G. Efficient Coalescent Simulation and Genealogical Analysis for Large Sample Sizes. *PLoS Comput. Biol.* **12**, e1004842 (2016).
42. Haller, B. C. & Messer, P. W. SLiM 3: Forward Genetic Simulations Beyond the Wright-Fisher Model. *Mol. Biol. Evol.* **36**, 632–637 (2019).
43. Mohle, M. & Sagitov, S. A Classification of Coalescent Processes for Haploid Exchangeable Population Models. *Ann. Probab.* **29**, 1547–1562 (2001).
44. Virtanen, P. *et al.* SciPy 1.0: fundamental algorithms for scientific computing in Python. *Nat. Methods* **17**, 261–272 (2020).
45. Teukolsky, S. A., Flannery, B. P., Press, W. H. & Vetterling, W. Numerical recipes in C. *SMR Rapp.* **693**, 59–70 (1992).
46. Powell, M. J. D. An efficient method for finding the minimum of a function of several variables without calculating derivatives. *Comput. J.* **7**, 155–162 (1964).
47. Chang, C. C. *et al.* Second-generation PLINK: rising to the challenge of larger and richer datasets. *Gigascience* **4**, 7 (2015).
48. Alexander, D. H., Novembre, J. & Lange, K. Fast model-based estimation of ancestry in unrelated individuals. *Genome Res.* **19**, 1655–1664 (2009).
49. Hoban, S. *et al.* Monitoring status and trends in genetic diversity for the Convention on Biological Diversity: An ongoing assessment of genetic indicators in nine countries. *Conserv. Lett.* **16**, (2023).
50. Wakeley, J. & Aliacar, N. Gene genealogies in a metapopulation. *Genetics* **159**, 893–905 (2001).
51. Barton, N. H. & Wilson, I. Genealogies and geography. *Philos. Trans. R. Soc. Lond. B Biol. Sci.* **349**, 49–59 (1995).
52. Rauch, E. M. & Bar-Yam, Y. Theory predicts the uneven distribution of genetic diversity within species. *Nature* **431**, 449–452 (2004).
53. Wright, S., Dobzhansky, T. & Hovanitz, W. Genetics of Natural Populations. VII. the Allelism of Lethals in the Third Chromosome of Drosophila Pseudoobscura. *Genetics* **27**, 363–394 (1942).
54. Matthews, T. J., Triantis, K. A. & Whittaker, R. J. *The Species-Area Relationship: Theory and Application*. (Cambridge University Press, 2021).
55. Macarthur, R. H. & Wilson, E. O. *The Theory of Island Biogeography*. (Princeton University Press, 1967).

bioRxiv preprint doi: https://doi.org/10.1101/2024.10.21.619096; this version posted October 22, 2024. The copyright holder for this preprint (which was not certified by peer review) is the author/funder, who has granted bioRxiv a license to display the preprint in perpetuity. It is made available under aCC-BY 4.0 International license.

56. Hu, X.-S., He, F. & Hubbell, S. P. Neutral theory in macroecology and population genetics. *Oikos* **113**, 548–556 (2006).
57. Storch, D., Keil, P. & Jetz, W. Universal species-area and endemics-area relationships at continental scales. *Nature* **488**, 78–81 (2012).
58. Thomas, C. D. *et al.* Extinction risk from climate change. *Nature* **427**, 145–148 (2004).
59. IPBES. Global assessment report on biodiversity and ecosystem services of the Intergovernmental Science-Policy Platform on Biodiversity and Ecosystem Services. Preprint at https://doi.org/10.5281/ZENODO.3831673 (2019).
60. Ma, Z. S. DAR (diversity-area relationship): Extending classic SAR (species-area relationship) for biodiversity and biogeography analyses. *Ecol. Evol.* **8**, 10023–10038 (2018).
61. Matthews, T., Guilhaumon, F. & Cazelles, K. *txm676/sars: Sars R Package*. (Zenodo, 2019). doi:10.5281/ZENODO.2573067.

bioRxiv preprint doi: https://doi.org/10.1101/2024.10.21.619096; this version posted October 22, 2024. The copyright holder for this preprint (which was not certified by peer review) is the author/funder, who has granted bioRxiv a license to display the preprint in perpetuity. It is made available under aCC-BY 4.0 International license.

Supplemental Figures

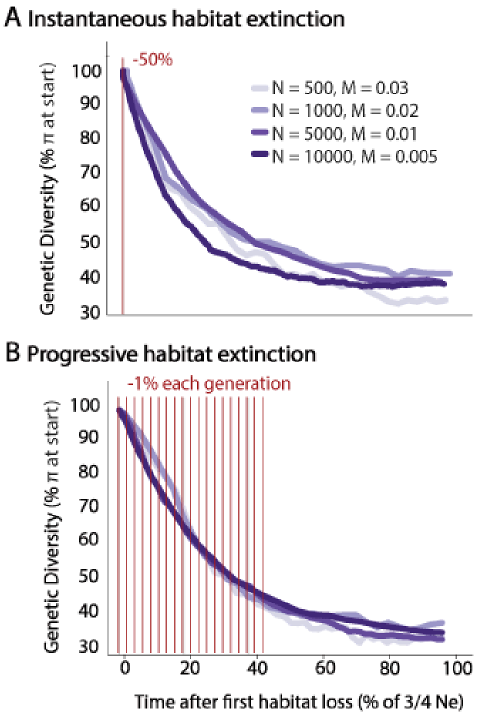

Fig. S1 | Temporal dynamics of genetic diversity

(A) Trajectories of genetic diversity loss after 50% instantaneous extinction of habitat has halted. Parameters that alter these dynamics include population size (N) and migration rate (M). (B) Trajectories of genetic diversity loss with gradual extinction of 50% habitat. Gradual habitat loss was kept at 1% of habitat loss per ~11 generations. Colors represent different values of population size and migration rate used, and are consistent between A and B.

bioRxiv preprint doi: https://doi.org/10.1101/2024.10.21.619096; this version posted October 22, 2024. The copyright holder for this preprint (which was not certified by peer review) is the author/funder, who has granted bioRxiv a license to display the preprint in perpetuity. It is made available under aCC-BY 4.0 International license.

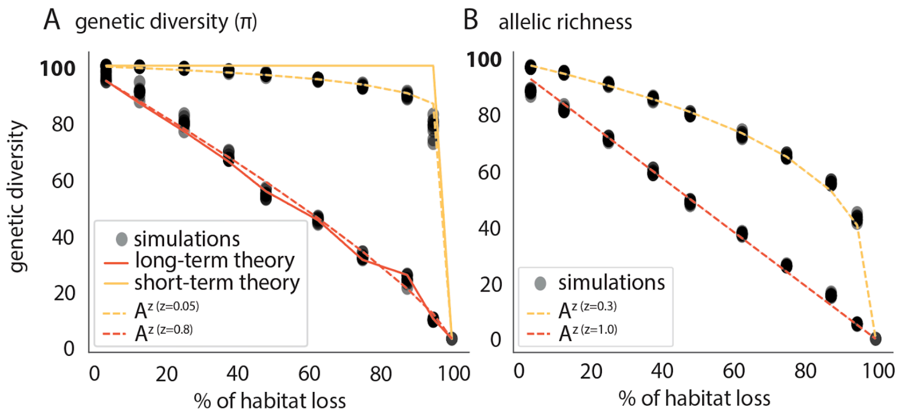

Fig. S2 | Relationship between genetic diversity and habitable area

In log space, genetic diversity and habitable area follow a power law relationship, genetic diversity = A^z for both short and long-term trajectories of **(A)** π and **(B)** allelic richness. Solid lines illustrate the genetic diversity trajectories seen using our theoretical and simulation-based framework. Dotted lines indicate the power law relationship using the parameters corresponding to both short and long-term respectively. Black dots represent genetic diversity trajectories using our simulations. In red are short-term estimates while in orange are long-term estimates. **(A)** In the short-term, z=0.05 while in the long-term, z=0.8 . **(B)** In the short-term, z=0.3 while in the long-term, z=1.0.

bioRxiv preprint doi: https://doi.org/10.1101/2024.10.21.619096; this version posted October 22, 2024. The copyright holder for this preprint (which was not certified by peer review) is the author/funder, who has granted bioRxiv a license to display the preprint in perpetuity. It is made available under aCC-BY 4.0 International license.

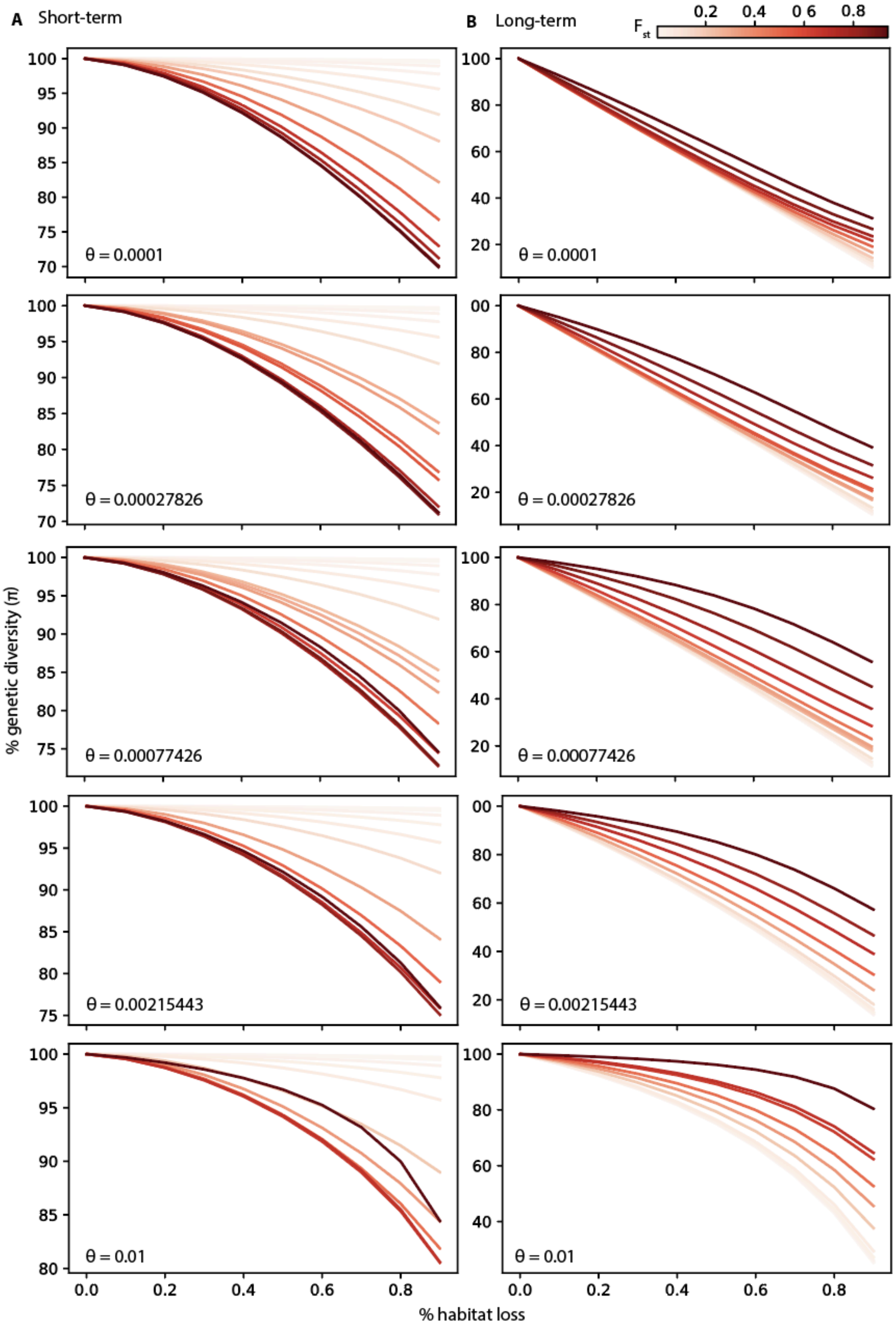

Fig. S3 | Relationship between genetic diversity and habitable area across F_{ST} and θ values for edge contraction scenarios

Theoretical projections of genetic diversity across different F_{ST} and θ values using wfmoments. Different hues of red represent different F_{ST} values, as indicated via the colour bar. Each row represents a different θ value tested. Each column represents genetic diversity trajectories seen in the short and long-term.

bioRxiv preprint doi: https://doi.org/10.1101/2024.10.21.619096; this version posted October 22, 2024. The copyright holder for this preprint (which was not certified by peer review) is the author/funder, who has granted bioRxiv a license to display the preprint in perpetuity. It is made available under aCC-BY 4.0 International license.

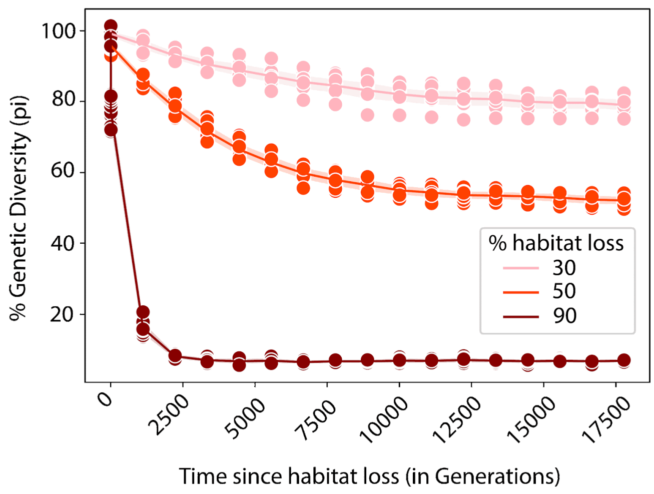

Fig. S4 | Genetic diversity extinction with species range contraction over time

Loss of genetic diversity (π) from edge range contraction (30%, 50%, 90%) over time (in Generations). Each dot represents an estimate of genetic diversity for that specific % of habitat loss at that specific time point. A total of 9 replicates were run for 30, 50, 90% habitat loss at every specific time point.

bioRxiv preprint doi: https://doi.org/10.1101/2024.10.21.619096; this version posted October 22, 2024. The copyright holder for this preprint (which was not certified by peer review) is the author/funder, who has granted bioRxiv a license to display the preprint in perpetuity. It is made available under aCC-BY 4.0 International license.

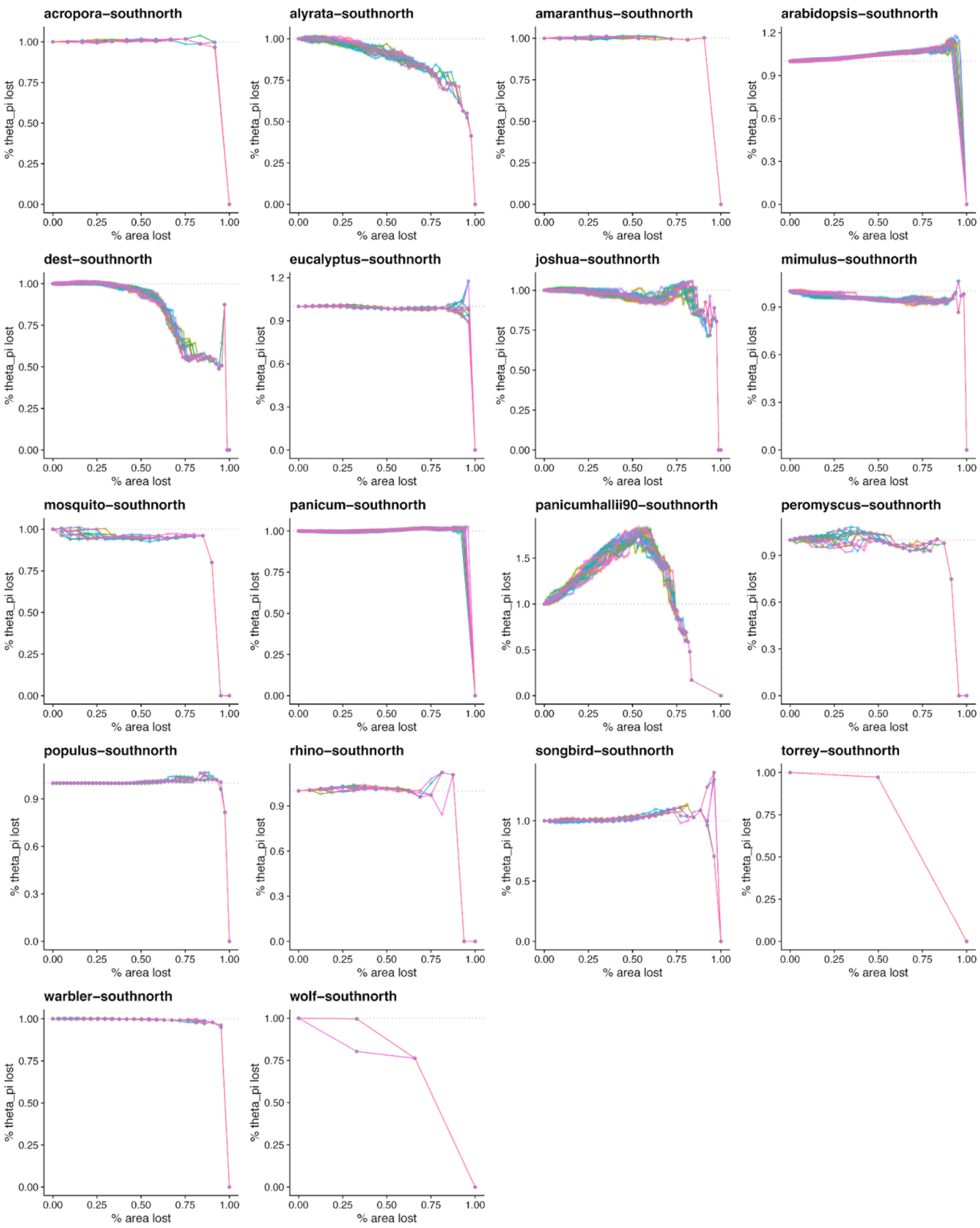

Fig. S5 | Genetic diversity trajectories across species

For each species, we plot short-term trajectories for north-south extinction. Different colors represent different replicates. The x-axis represents the percentage of area lost and the y-axis shows the percentage of pairwise differences (π) left in a species.

bioRxiv preprint doi: https://doi.org/10.1101/2024.10.21.619096; this version posted October 22, 2024. The copyright holder for this preprint (which was not certified by peer review) is the author/funder, who has granted bioRxiv a license to display the preprint in perpetuity. It is made available under aCC-BY 4.0 International license.

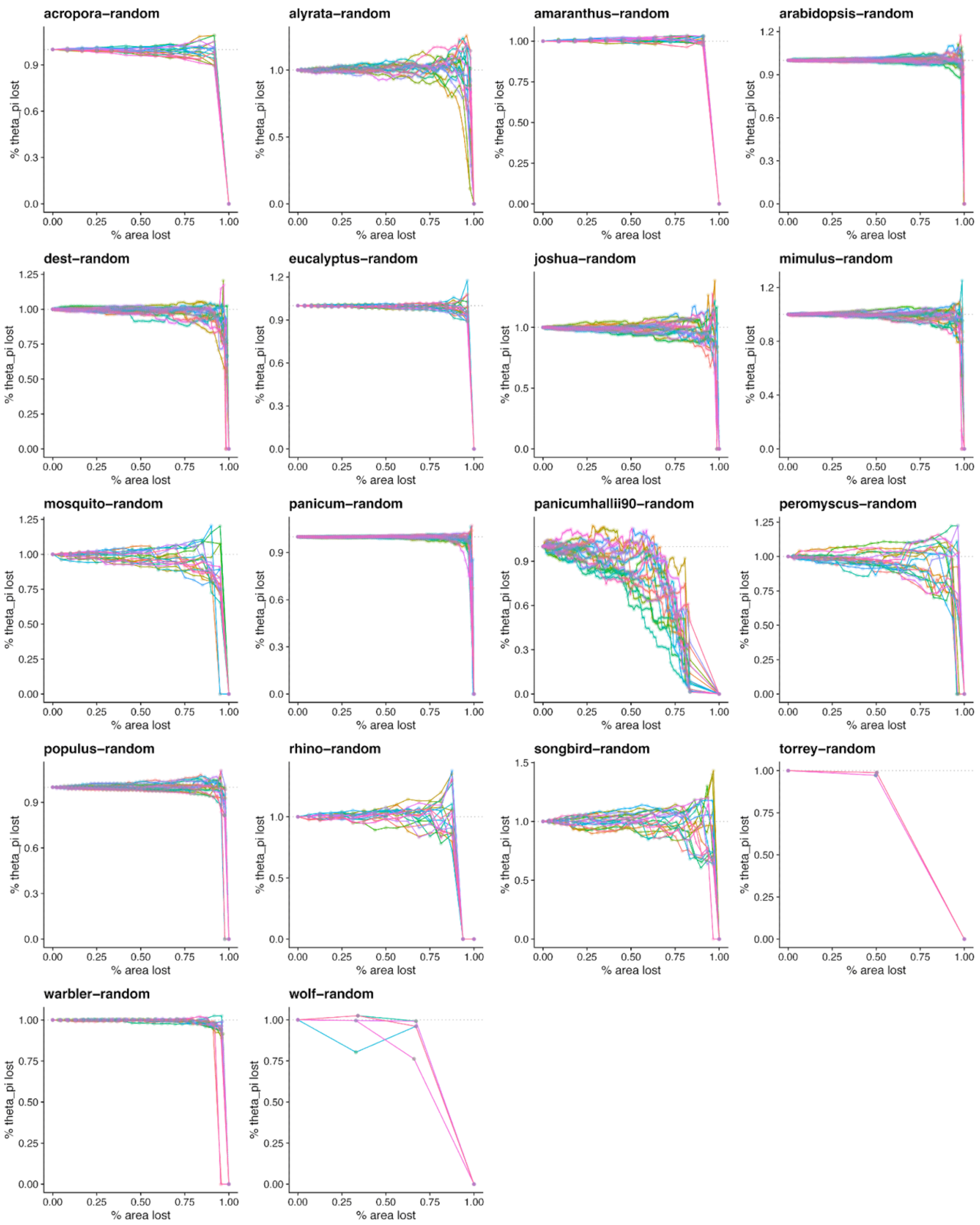

Fig. S6 | Genetic diversity trajectories across species

For each species, we plot short-term trajectories for random extinction. Different colors represent different replicates. The x-axis represents the percentage of area lost and the y-axis shows the percentage of pairwise differences (π) left in a species.

bioRxiv preprint doi: https://doi.org/10.1101/2024.10.21.619096; this version posted October 22, 2024. The copyright holder for this preprint (which was not certified by peer review) is the author/funder, who has granted bioRxiv a license to display the preprint in perpetuity. It is made available under aCC-BY 4.0 International license.

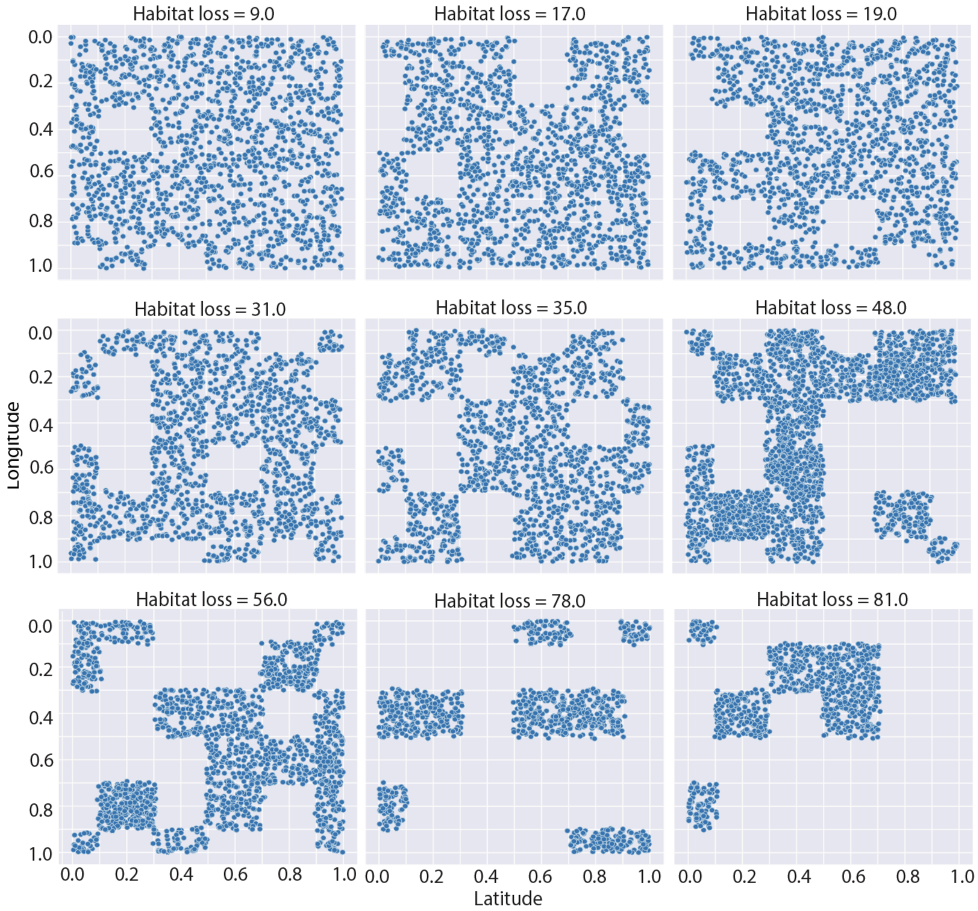

Fig. S7 | Habitat fragmentation maps

Habitat fragmentation maps shown across a range of habitat loss scenarios within the simulation. Each map is a 10×10 grid with each dot representing an individual along a 2-D coordinate system (latitude, longitude). Here we show how habitat fragmentation occurs within simulation space, with empty boxes representing the "extincted" habitat.

bioRxiv preprint doi: https://doi.org/10.1101/2024.10.21.619096; this version posted October 22, 2024. The copyright holder for this preprint (which was not certified by peer review) is the author/funder, who has granted bioRxiv a license to display the preprint in perpetuity. It is made available under aCC-BY 4.0 International license.

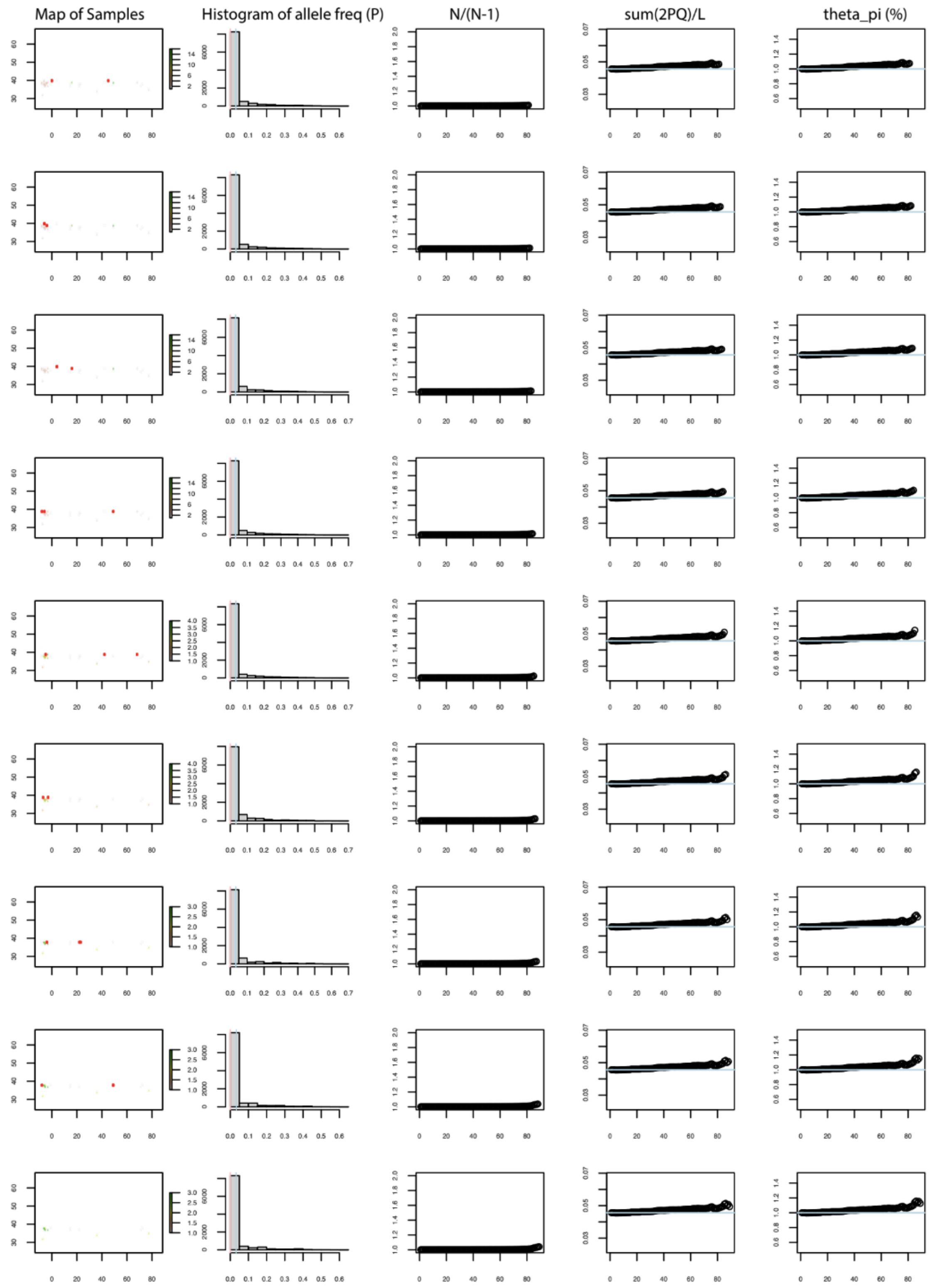

Fig. S8 | Empirical extinction simulations for *Arabidopsis thaliana*

Tracking the extinction process for *Arabidopsis thaliana* south-north empirical extinction simulations. Each row represents a specific time point in the simulation extinction process.

bioRxiv preprint doi: https://doi.org/10.1101/2024.10.21.619096; this version posted October 22, 2024. The copyright holder for this preprint (which was not certified by peer review) is the author/funder, who has granted bioRxiv a license to display the preprint in perpetuity. It is made available under aCC-BY 4.0 International license.

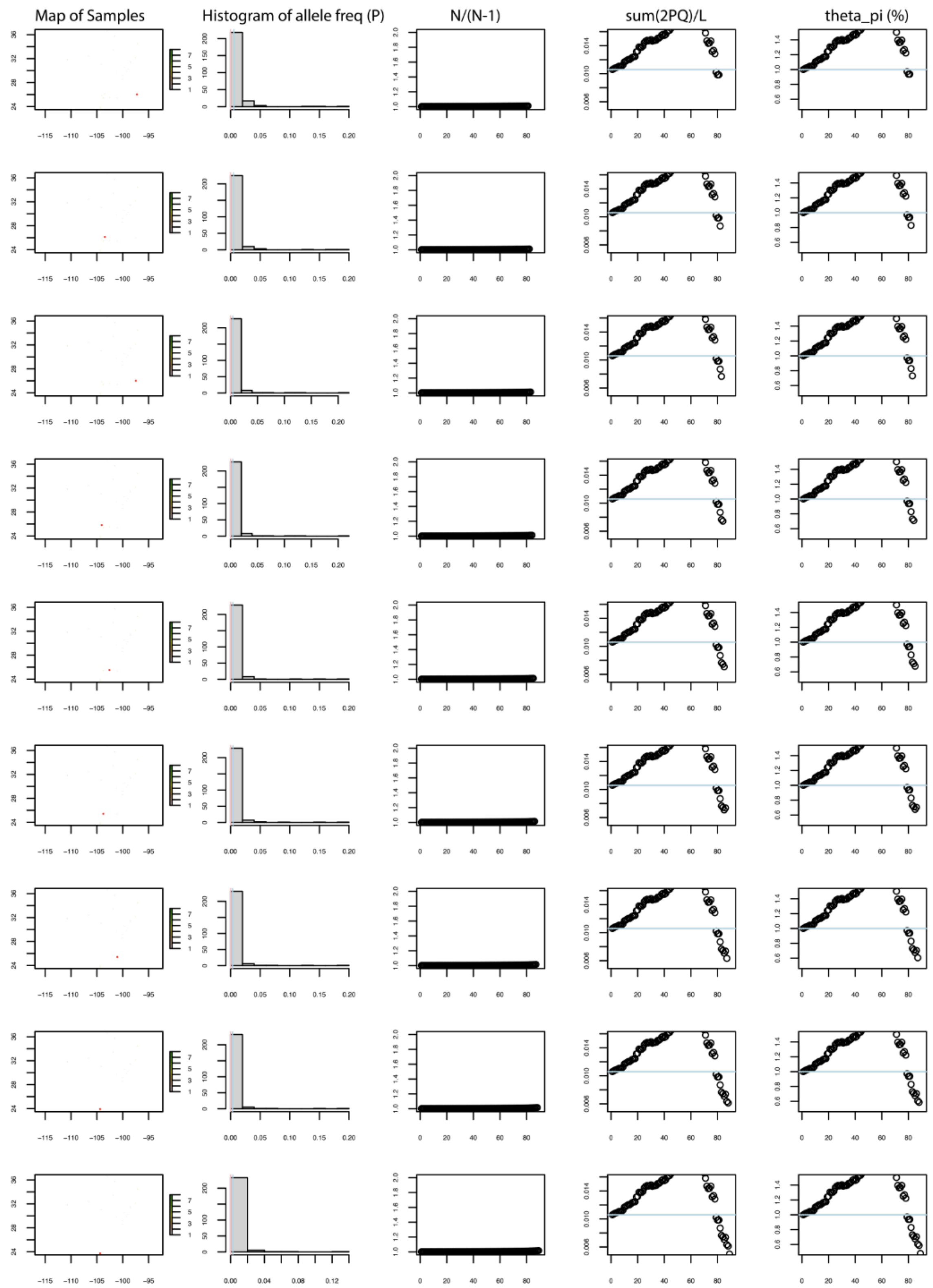

Fig. S9 | Empirical extinction simulations for *Panicum hallii*

Tracking the extinction process for *Panicum hallii* south-north empirical simulations. Each row represents a specific time point in the simulation extinction process.

bioRxiv preprint doi: https://doi.org/10.1101/2024.10.21.619096; this version posted October 22, 2024. The copyright holder for this preprint (which was not certified by peer review) is the author/funder, who has granted bioRxiv a license to display the preprint in perpetuity. It is made available under aCC-BY 4.0 International license.

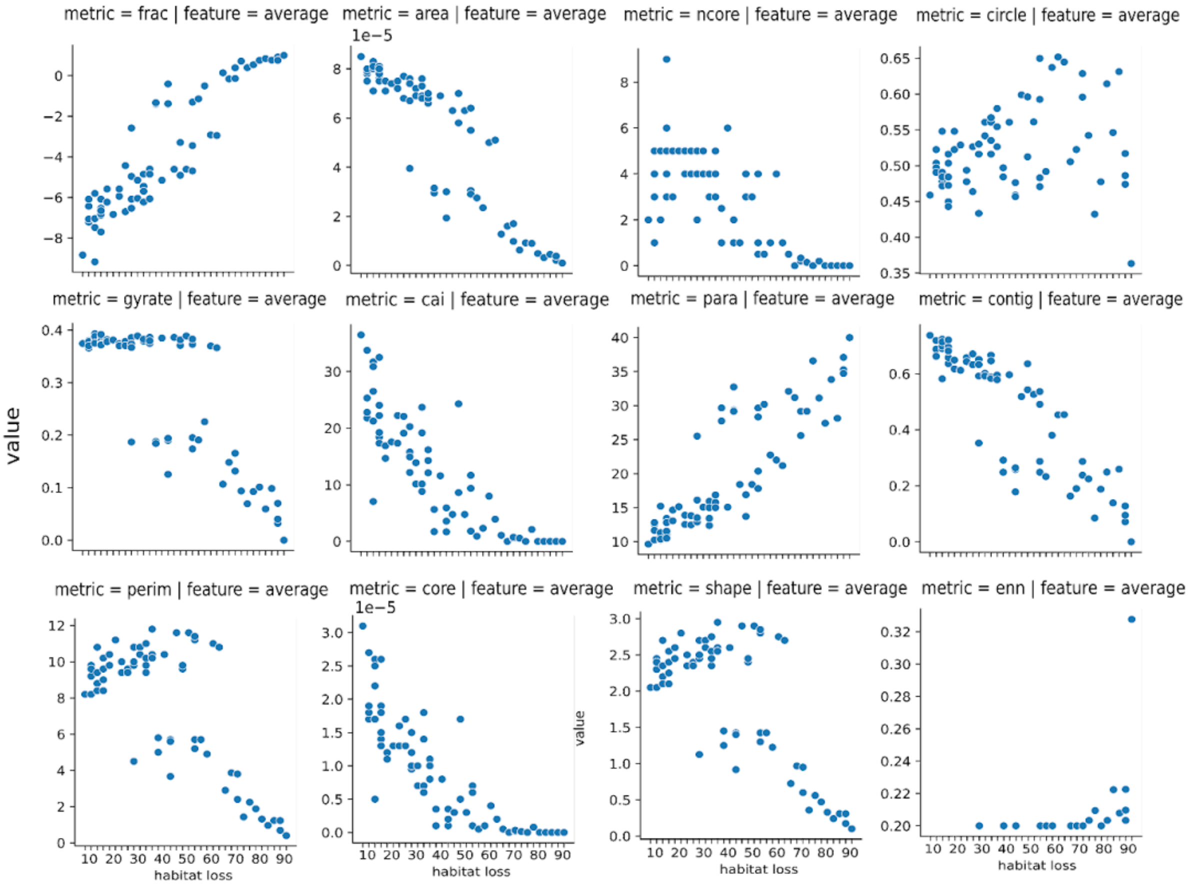

Fig. S10 | Connectivity metrics across habitat fragmentation maps (6×6) in SLiM simulations

Each plot represents a different plotted connectivity metric and the corresponding aggregation function used to report each metric. Blue dots represent different replicates for that % habitat loss. In total, 90 different simulation runs with varying habitat fragmentation maps are displayed in this figure. We considered % habitat loss ranging from 10% to 90%, at 10% increments. Metrics are selected by utilizing the landscape metrics R package.

bioRxiv preprint doi: https://doi.org/10.1101/2024.10.21.619096; this version posted October 22, 2024. The copyright holder for this preprint (which was not certified by peer review) is the author/funder, who has granted bioRxiv a license to display the preprint in perpetuity. It is made available under aCC-BY 4.0 International license.

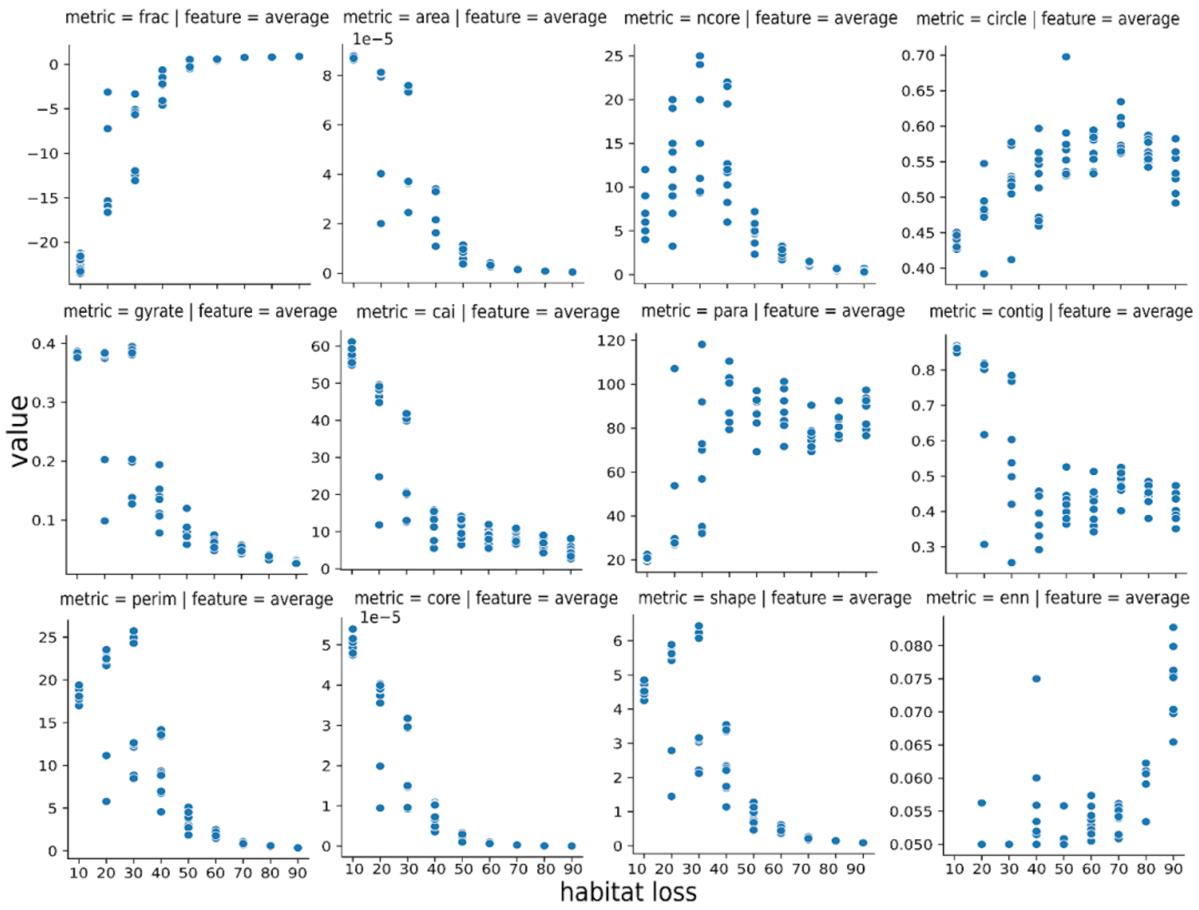

Fig. S11 | Connectivity metrics across habitat fragmentation maps (20x20) in SLiM simulations

Each plot represents a different plotted connectivity metric and the corresponding aggregation function used to report each metric. Blue dots represent different replicates for that % habitat loss. In total, 90 different simulation runs with varying habitat fragmentation maps are displayed in this figure. We considered % habitat loss ranging from 10% to 90%, at 10% increments. Metrics are selected by utilizing the landscapemetrics R package.

bioRxiv preprint doi: https://doi.org/10.1101/2024.10.21.619096; this version posted October 22, 2024. The copyright holder for this preprint (which was not certified by peer review) is the author/funder, who has granted bioRxiv a license to display the preprint in perpetuity. It is made available under aCC-BY 4.0 International license.

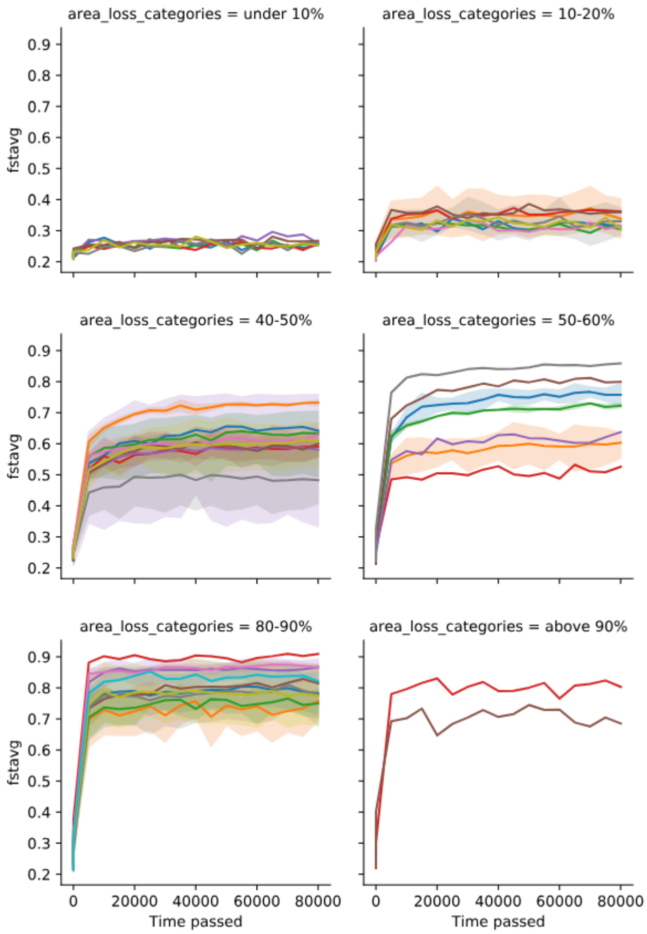

Fig. S12 | Increased F_{ST} in simulations with habitat fragmentation

We tracked individuals over 80,000 SLiM ticks after habitat fragmentation (at time=0). We tracked F_{ST} values of our populations across different area loss categories ranging from 0 to ~90%, totalling 121 different simulation runs. Colors represent different simulation runs. Shaded area showing 95% confidence intervals for certain runs that had corresponding replicates, while others are single runs indicating just a single simulation run.

bioRxiv preprint doi: https://doi.org/10.1101/2024.10.21.619096; this version posted October 22, 2024. The copyright holder for this preprint (which was not certified by peer review) is the author/funder, who has granted bioRxiv a license to display the preprint in perpetuity. It is made available under aCC-BY 4.0 International license.

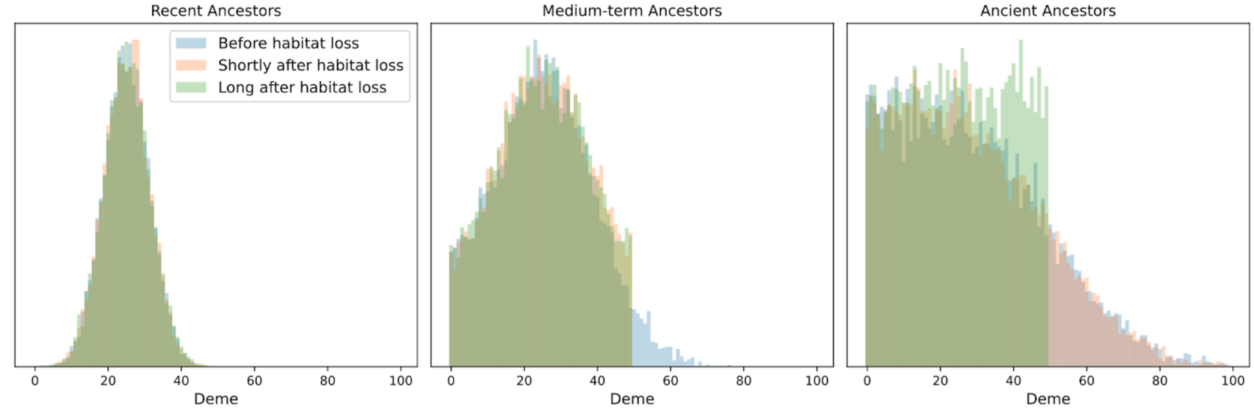

Fig. S13 | Distribution of ancestors across landscape map after habitat loss

We consider a 1D habitat of 100 demes (labeled 0, 1, … 99). All individuals are sampled in *deme 25*. At each generation, an individual has a 0.2 probability of having their ancestor come from the deme to left (if it exists) and 0.2 probability of having their ancestor come from the deme to the right (if it exists) and their ancestor comes from the same deme otherwise. "Recent ancestors" are sampled 100 generations prior to sampling time, "Medium-term ancestors" are sampled 500 generations prior to sampling time, and "Ancient ancestors" are sampled 1500 generations prior to sampling time. For the "Shortly after habitat loss" scenario, we assume that demes 50 and above became uninhabitable 500 generations ago, and for the "Long after habitat loss" scenario, we assume that demes 50 and above went extinct more than 1500 generations ago.

bioRxiv preprint doi: https://doi.org/10.1101/2024.10.21.619096; this version posted October 22, 2024. The copyright holder for this preprint (which was not certified by peer review) is the author/funder, who has granted bioRxiv a license to display the preprint in perpetuity. It is made available under aCC-BY 4.0 International license.

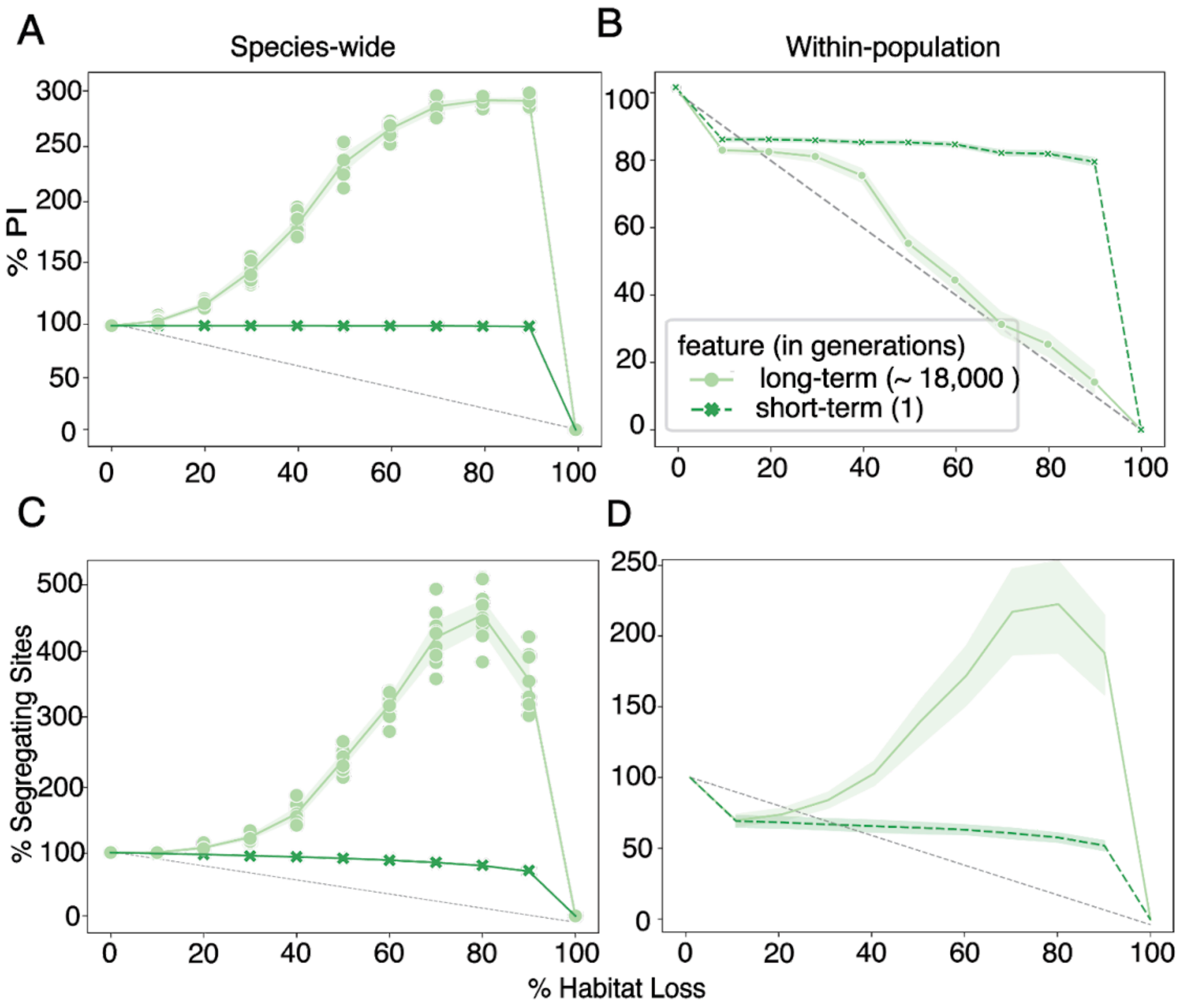

Fig. S14 | Genetic diversity metrics after habitat fragmentation

Species-wide genetic diversity, (A) π and (C) allelic richness (% segregating sites), across different percentages of habitat loss. Simulations were run for 9 replicates. Light green solid line shows average long-term genetic diversity projections using our simulation framework, with shaded area showing 95% confidence intervals. Dark green dotted line shows average short-term genetic diversity projections using our simulation framework. Each dot represents an average measure of π across a 100m-by-100m habitat map at specific percentages of habitat loss for a specific replicate, where each replicate is a different habitat fragmentation map. Light gray line indicates the y=x relationship. Within-population genetic diversity , (B) π and (D) allelic richness (% segregating sites), across different percentages of habitat loss. Light green solid line represents the average within-population long-term genetic diversity across all 5m-by-5m grids within a 100m-by-100m habitat map across all replicates, where each replicate is a different habitat fragmentation map, with shaded area showing 95% confidence intervals. Dark green dotted line represents the average within-population short-term genetic diversity across all 5m-by-5m grids within a 100m-by-100m habitat map across all replicates, where each replicate is a different habitat fragmentation map, with shaded area showing 95% confidence intervals.

bioRxiv preprint doi: https://doi.org/10.1101/2024.10.21.619096; this version posted October 22, 2024. The copyright holder for this preprint (which was not certified by peer review) is the author/funder, who has granted bioRxiv a license to display the preprint in perpetuity. It is made available under aCC-BY 4.0 International license.

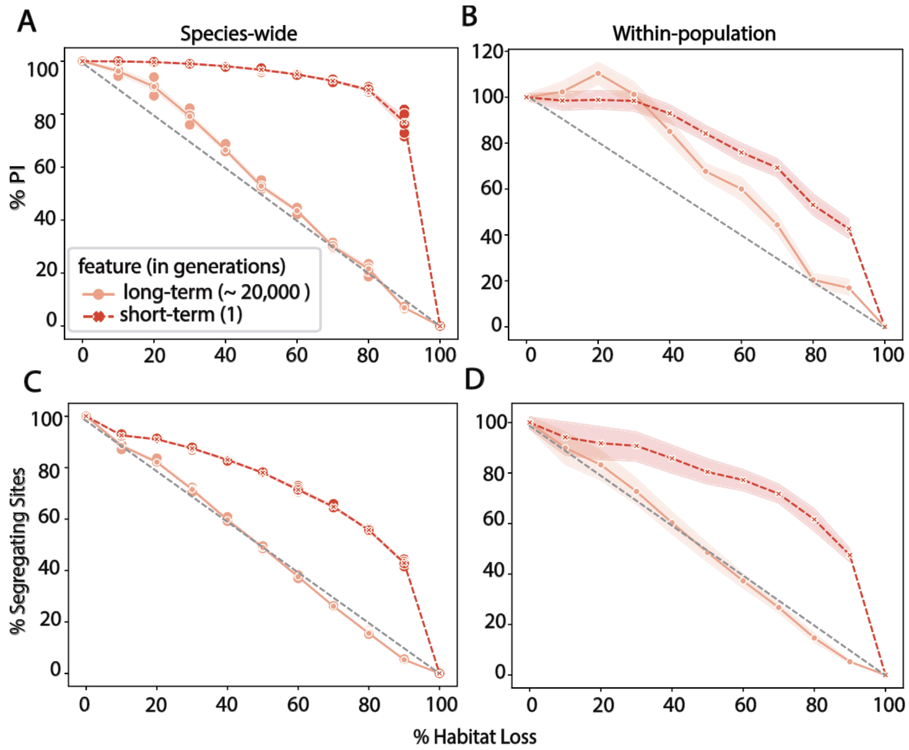

Fig. S15 | Genetic diversity metrics after habitat loss from one edge

Species-wide genetic diversity, (A) π and (C) allelic richness (% segregating sites *S*), across different percentages of habitat loss. Simulations were run for 9 replicates in 10 different area loss percentages. Light red solid line shows average long-term genetic diversity projections using our simulation framework. Dark red dotted line shows average short-term genetic diversity projections using our simulation framework. Each dot represents an average measure of π across a 100x100 units habitat map at specific percentages of habitat loss for a specific replicate. Light gray line indicates the y=x relationship. Within-population genetic diversity , (B) π and (D) allelic richness (% segregating sites), across different percentages of habitat loss. Light red solid line represents the average within-population long-term genetic diversity across all 5x5 units grids within a 100x100 units habitat map across all replicates, with shaded area showing 95% confidence intervals. Dark red dotted line represents the average within-population short-term genetic diversity across all 5x5 units grids within a 100x100 units habitat map across all replicates, with shaded area showing 95% confidence intervals.

bioRxiv preprint doi: https://doi.org/10.1101/2024.10.21.619096; this version posted October 22, 2024. The copyright holder for this preprint (which was not certified by peer review) is the author/funder, who has granted bioRxiv a license to display the preprint in perpetuity. It is made available under aCC-BY 4.0 International license.

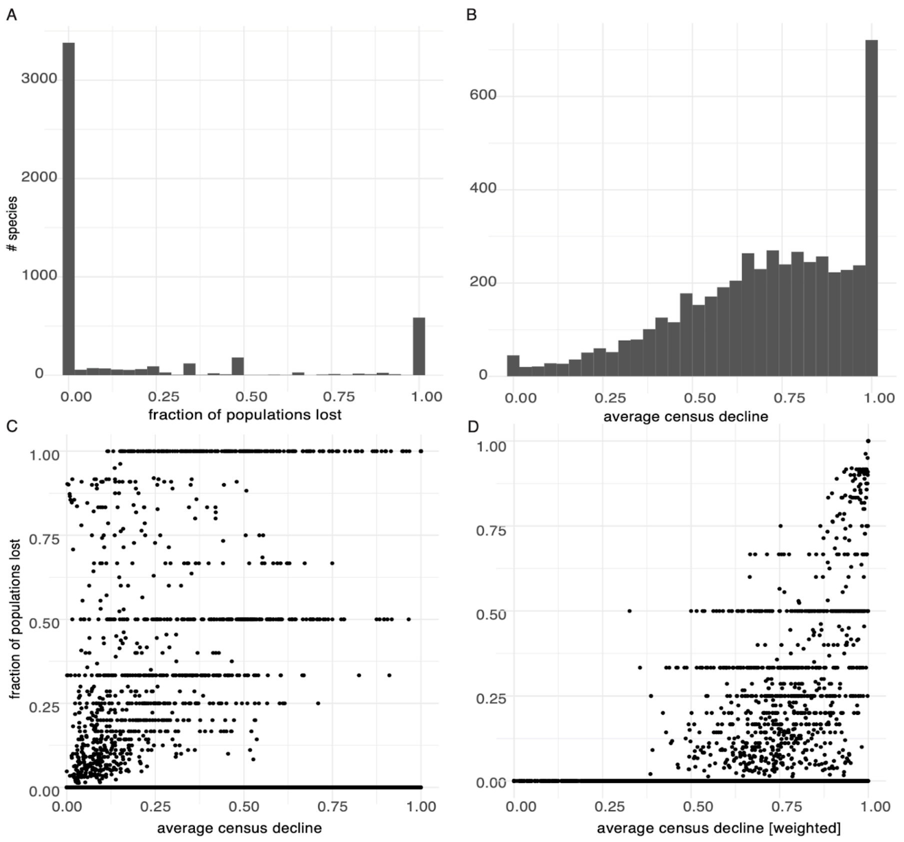

Fig. S16 | LPI raw summary data

Using data from the Living Planet Index 2024, we tracked the (A) fraction of populations lost and (B) the average census decline from 1967 to 2020 for 41986 populations across 5579 species total. Histograms show the number of species that have that fraction of population lost and average census decline respectively. (C) Scatterplots of the fraction of population lost with average census decline. (D) Scatterplot of the fraction of population lost with average census decline, weighted by the original population size of the species. Each dot represents a species.

bioRxiv preprint doi: https://doi.org/10.1101/2024.10.21.619096; this version posted October 22, 2024. The copyright holder for this preprint (which was not certified by peer review) is the author/funder, who has granted bioRxiv a license to display the preprint in perpetuity. It is made available under aCC-BY 4.0 International license.

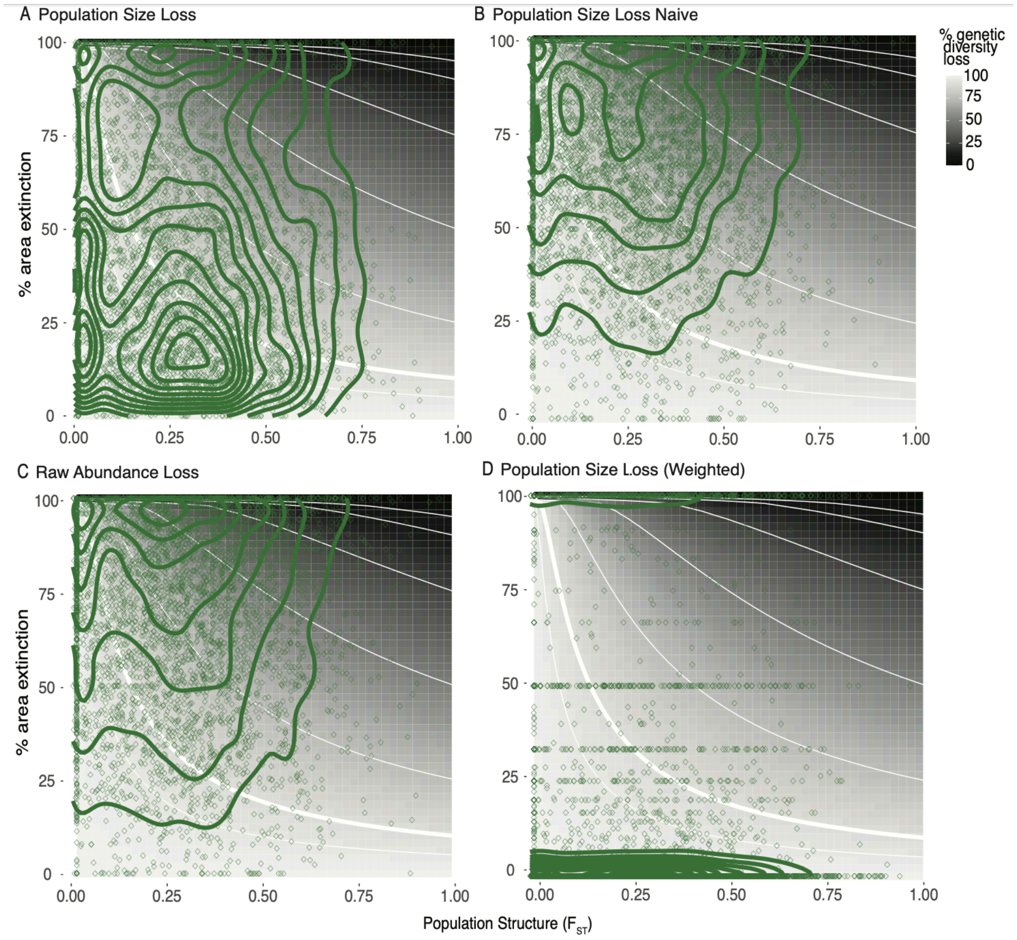

Fig. S17 | LPI summary data and genetic diversity projections

Using data from the Living Planet Index 2024, we tracked the (A) fraction of populations lost and (B) the average census decline from 1967 to 2020 for 41986 populations across 5579 species total. Histograms show the number of species that have that fraction of population lost and average census decline respectively. (C) Scatterplots of the fraction of population lost with average census decline. (D) Scatterplot of the fraction of population lost with average census decline, weighted by the original population size of the species. Each dot represents a species. Utilizing population size declines of 5579 species tracked over 3 decades, we transformed the data into 4 separate measurements: (A) Population size loss, (B) Population size loss naive, (C) Raw abundance loss and (D) Population Size Loss (Weighted). Estimated percentage of genetic diversity (allelic richness) loss in the short-term using the MAR approach (mean z_{MAR} =0.3, sd = 0.24*)* is represented as a gradient of white to gray, with corresponding isolines in white.

bioRxiv preprint doi: https://doi.org/10.1101/2024.10.21.619096; this version posted October 22, 2024. The copyright holder for this preprint (which was not certified by peer review) is the author/funder, who has granted bioRxiv a license to display the preprint in perpetuity. It is made available under aCC-BY 4.0 International license.

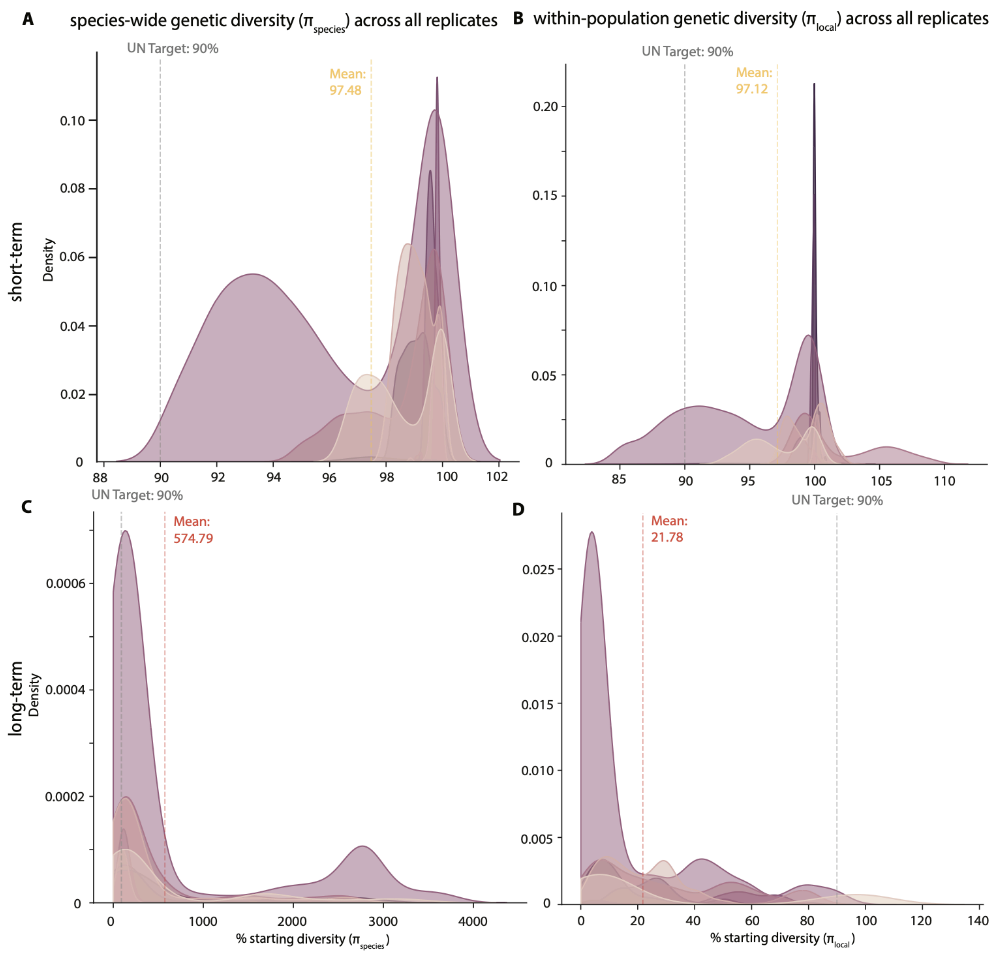

Fig. S18 | Density distributions of genetic diversity metrics across LPI populations under a habitat fragmentation using WFmoments framework

Species-wide genetic diversity ($\pi_{species}$) and within-population genetic diversity (π_{local}) across 10 replicates within a habitat fragmentation framework, where each replicate represents a different habitat fragmentation map. Area reduction was estimated using the population abundance trends from the Living Planet Index across ~4 decades. Gray lines indicate the removed preliminary United Nations target on preserving 90% genetic diversity. (**A**) Short-term species-wide genetic diversity ($\pi_{species}$) (**B**) Short-term within-population genetic diversity (π_{local}). Yellow line indicates the mean value across all 10 replicates. (**C**) Long-term species-wide genetic diversity ($\pi_{species}$) (**D**) Long-term within-population genetic diversity (π_{local}). Red line indicates the mean value across all 10 replicates.

bioRxiv preprint doi: https://doi.org/10.1101/2024.10.21.619096; this version posted October 22, 2024. The copyright holder for this preprint (which was not certified by peer review) is the author/funder, who has granted bioRxiv a license to display the preprint in perpetuity. It is made available under aCC-BY 4.0 International license.

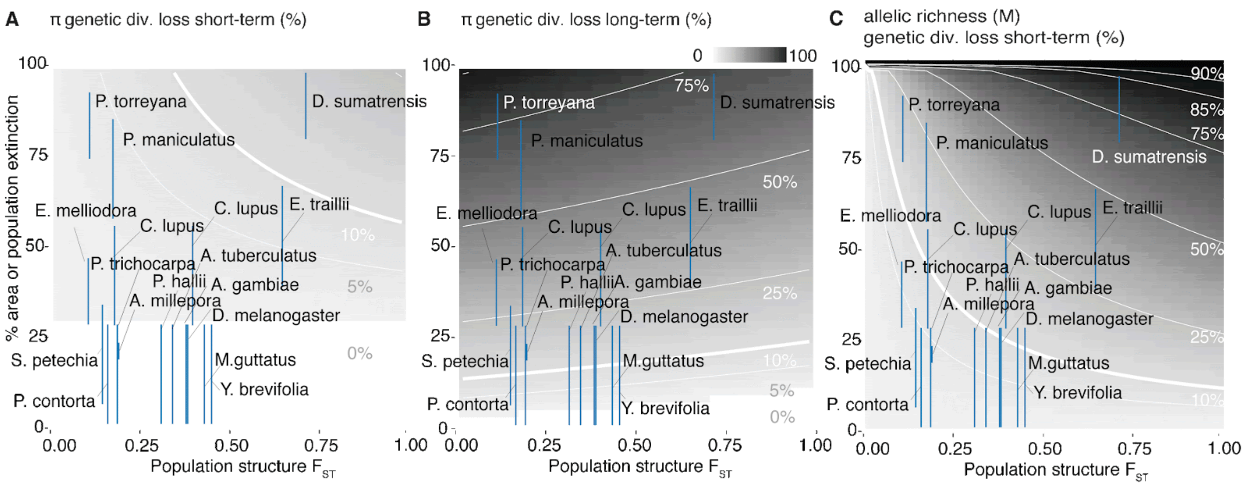

Fig. S19 | Genetic diversity loss (nucleotide diversity π and allelic richness S) based on MAR and GDAR

(**A**) Relationship between population structure, F_{ST} and the percentage of area or population extinction in the short-term. Estimated percentage of genetic diversity loss (π) in the short-term is represented as a gradient of white to gray, with corresponding isolines in white. Blue lines represent individual populations of species across 17 species from publicly available datasets. As a proxy for area extinction, we utilized population size changes over time for populations tracked by the Living Planet Index, when available. If that was not available, we approximated area extinctions using their Red List categories. (**B**) Relationship between population structure, F_{ST} and the percentage of area or population extinction in the long-term. Estimated percentage of genetic diversity loss (π) in the long-term is represented as a gradient of white to black, with corresponding isolines in white. Explanations of blue lines are similar to A. (**C**) Relationship between population structure, F_{ST} and the percentage of area or population extinction in the short-term. Estimated percentage of genetic diversity loss, allelic richness (M) or segregating sites (S) in the short-term is represented as a gradient of white to black, with corresponding isolines in white. Explanations of blue lines are similar to A.

bioRxiv preprint doi: https://doi.org/10.1101/2024.10.21.619096; this version posted October 22, 2024. The copyright holder for this preprint (which was not certified by peer review) is the author/funder, who has granted bioRxiv a license to display the preprint in perpetuity. It is made available under aCC-BY 4.0 International license.

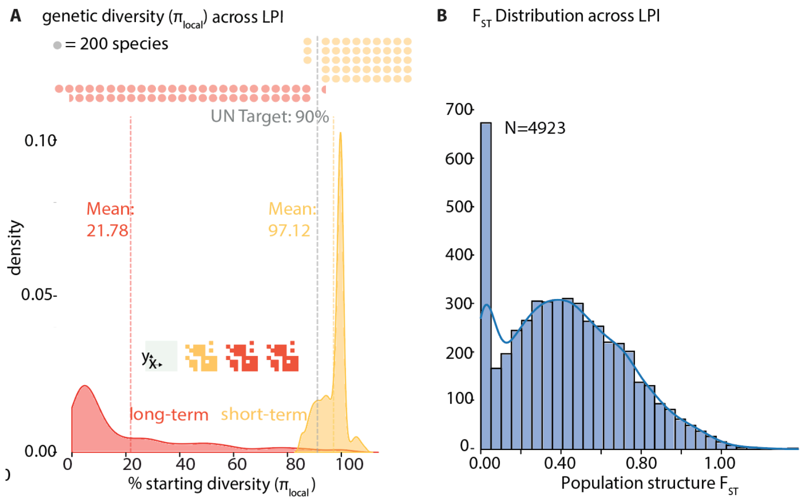

Fig. S20 | Genetic diversity loss (nucleotide diversity π) and F_{ST} across species

(**A**) Grey dotted line represents the 90% removed preliminary United Nations Target. Data used is from the 31,821 populations of 4,923 species of mammal, bird, reptile, amphibian and fish from the Living Planet Index (LPI). Yellow line represents projected π_{local} short-term estimates for populations in the LPI. Dotted yellow line represents the mean π_{local} short-term estimates for populations in LPI. Red line represents projected π_{local} long-term estimates for populations in the LPI. Dotted red line represents the mean π_{local} long-term estimates for populations in LPI. Projections use habitat fragmentation loss as illustrated in the cartoon and use power law estimates of short-term and long-term genetic diversity for π_{local} obtained from our theoretical and simulation-based framework. Circles show the number of species projected to be above or below the 90% UN proposed target. Yellow circles represent the number of species using short-term projections while red circles represent the number of species using long-term projections. Each circle represents 200 species. (**B**) Histogram showing F_{ST} distribution across LPI populations sampled from a truncated Normal distribution with mean and sd calibrated on empirical data from plant and animal species.

bioRxiv preprint doi: https://doi.org/10.1101/2024.10.21.619096; this version posted October 22, 2024. The copyright holder for this preprint (which was not certified by peer review) is the author/funder, who has granted bioRxiv a license to display the preprint in perpetuity. It is made available under aCC-BY 4.0 International license.

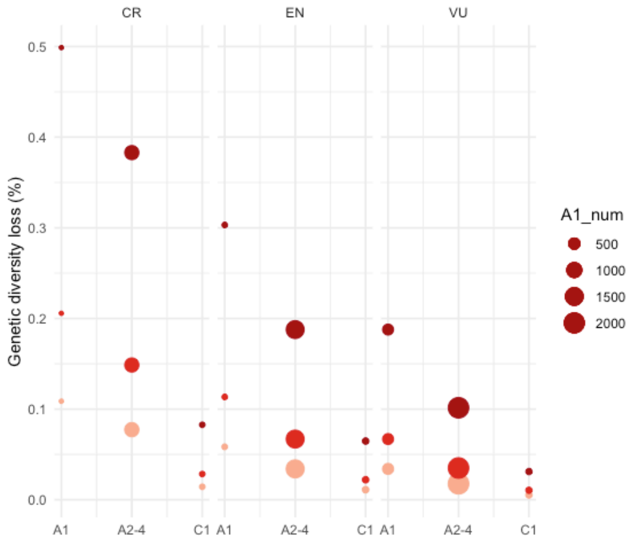

Fig. S21 | Translation of Red list genetic diversity loss (allelic richness *S*) predictions using MAR

Utilizing the IUCN Red List criterias as proxy for habitat loss, we obtained values for A1, A2-4 and C1 across three threaten statuses: Critically Endangered (CR), Endangered (EN) and Vulnerable (VU). We utilized our MAR framework to predict genetic diversity losses using three different scaling factors, Dark red z=0.3, Mid red z=0.1, Light red z=0.05.

bioRxiv preprint doi: https://doi.org/10.1101/2024.10.21.619096; this version posted October 22, 2024. The copyright holder for this preprint (which was not certified by peer review) is the author/funder, who has granted bioRxiv a license to display the preprint in perpetuity. It is made available under aCC-BY 4.0 International license.

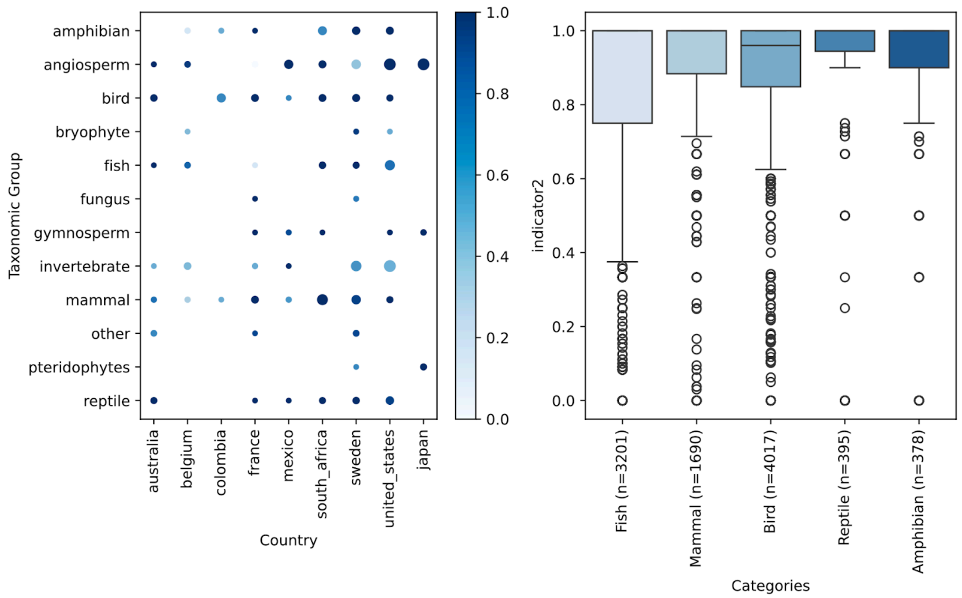

Fig. S22 | Summary of number of populations remaining from GBF indicator 2

We aggregate species collated from the Hoban et al. and LPI into 5-6 Taxonomic groups and plot the number of populations remaining (Indicator 2). (A) Data shown is from the paper Hoban et al. Size of dots indicate the number of species in that taxonomic group for that given country. Color represents the magnitude of Indicator 2. (B) Data shown is obtained from the Living Planet Index 2022 Database. Each point represents the indicator 2 value of a given species tracked in the LPI. Boxplots represent the distribution of Indicator 2 values across all species and all countries in that taxonomic group.

bioRxiv preprint doi: https://doi.org/10.1101/2024.10.21.619096; this version posted October 22, 2024. The copyright holder for this preprint (which was not certified by peer review) is the author/funder, who has granted bioRxiv a license to display the preprint in perpetuity. It is made available under aCC-BY 4.0 International license.

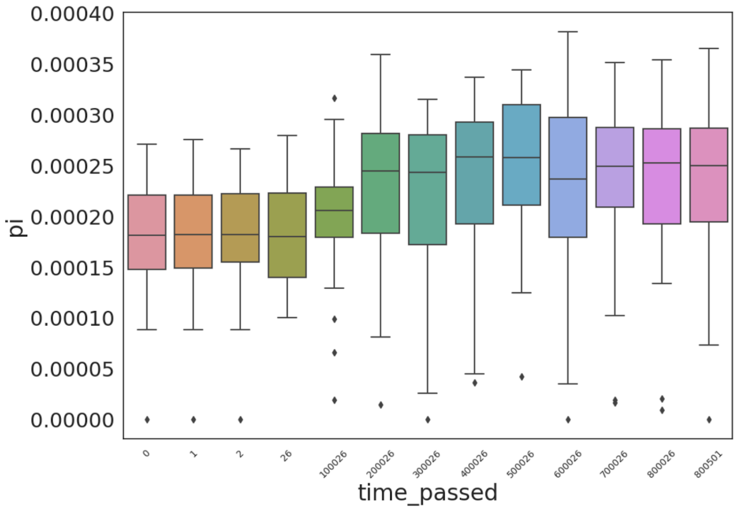

Fig. S23 | Genetic diversity (nucleotide diversity π) over burn-in time ensures stable initial conditions of area loss simulations.

Population-specific π over 800,000 slim ticks. Box and whisker plots show the distribution of π over all populations in the habitat. Black dots represent outlier populations. Color gradient represents time passed. Burn-in simulations were performed for our population size of 5,000 with migration rate = 0.005 and an overall F_{st} of ~0.3.

bioRxiv preprint doi: https://doi.org/10.1101/2024.10.21.619096; this version posted October 22, 2024. The copyright holder for this preprint (which was not certified by peer review) is the author/funder, who has granted bioRxiv a license to display the preprint in perpetuity. It is made available under aCC-BY 4.0 International license.

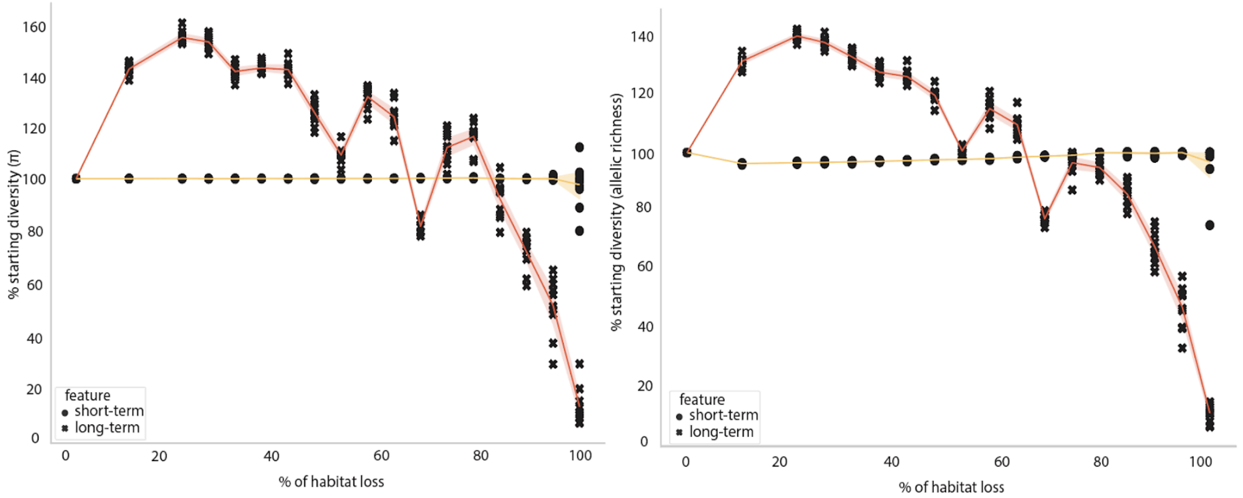

Fig. S24 | Genetic diversity (nucleotide diversity π and allelic richness *S*) across varying habitat loss with insufficient burn-in

Overlay of theoretical and simulation-based projections of short- and long-term genetic diversity loss, π, across different percentages of habitat loss from one edge. Simulations were run for 9 replicates. Red line shows average long-term genetic diversity projections. Yellow line shows average short-term genetic diversity projections. Shaded area showcases 95% confidence intervals across replicates.

bioRxiv preprint doi: https://doi.org/10.1101/2024.10.21.619096; this version posted October 22, 2024. The copyright holder for this preprint (which was not certified by peer review) is the author/funder, who has granted bioRxiv a license to display the preprint in perpetuity. It is made available under aCC-BY 4.0 International license.

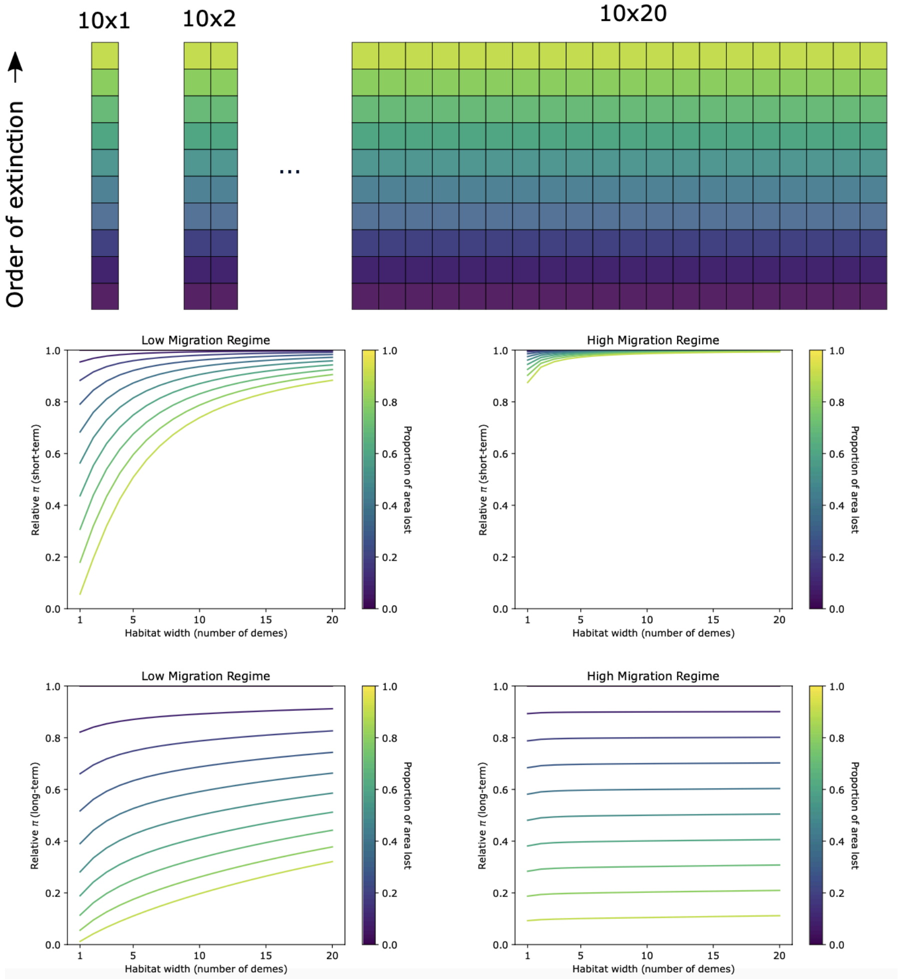

Fig. S25 | Genetic diversity trajectories across 1-D & 2-D habitats

Plots showing how genetic diversity in both short- and long-term change with geometry of habitat map and migration rate. Top row shows cartoons of habitat maps of varying sizes ranging from 10×1 to 10×20. Colors correspond to the amount of habitat loss incurred, with darker colors indicating lower losses while lighter colors indicating higher losses. Middle row shows short-term relative π across low and high migration regimes across different habitat widths. Bottom row shows long-term relative π across low and high migration regimes across different habitat widths. Each line represents a proportion of area loss across different habitat widths.

bioRxiv preprint doi: https://doi.org/10.1101/2024.10.21.619096; this version posted October 22, 2024. The copyright holder for this preprint (which was not certified by peer review) is the author/funder, who has granted bioRxiv a license to display the preprint in perpetuity. It is made available under aCC-BY 4.0 International license.

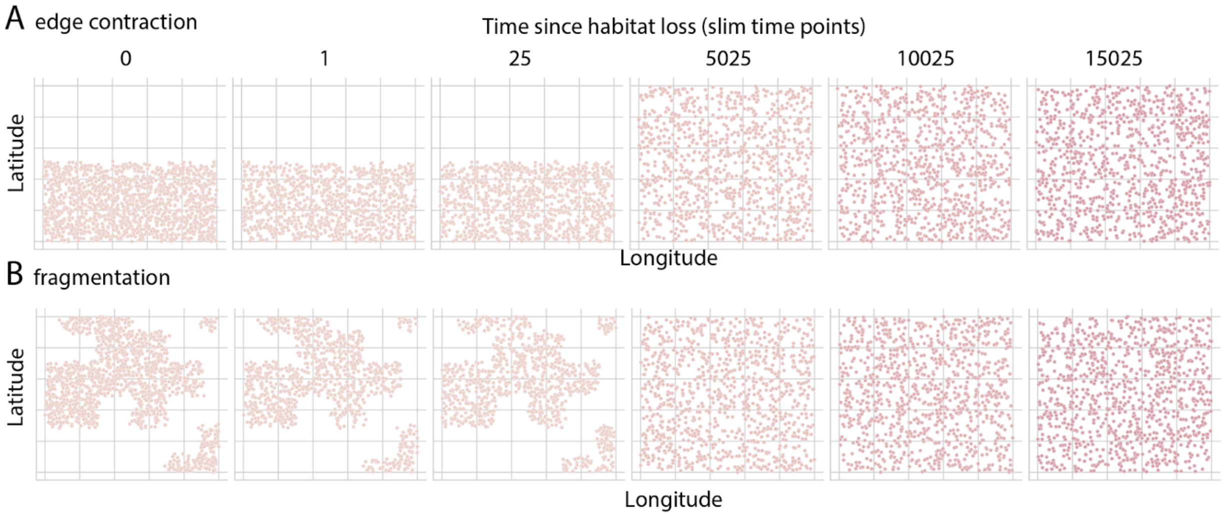

Fig. S26 | Habitat restoration maps

Cartoon showing habitat restoration within simulation space. (A) edge contraction (B) fragmentation maps. At time of habitat loss (Time=0), 50% of habitat loss is induced. Each dot represents an individual along a 2-D coordinate system (latitude, longitude). Empty boxes represent uninhabitable areas, individuals cannot disperse into these empty boxes.

bioRxiv preprint doi: https://doi.org/10.1101/2024.10.21.619096; this version posted October 22, 2024. The copyright holder for this preprint (which was not certified by peer review) is the author/funder, who has granted bioRxiv a license to display the preprint in perpetuity. It is made available under aCC-BY 4.0 International license.

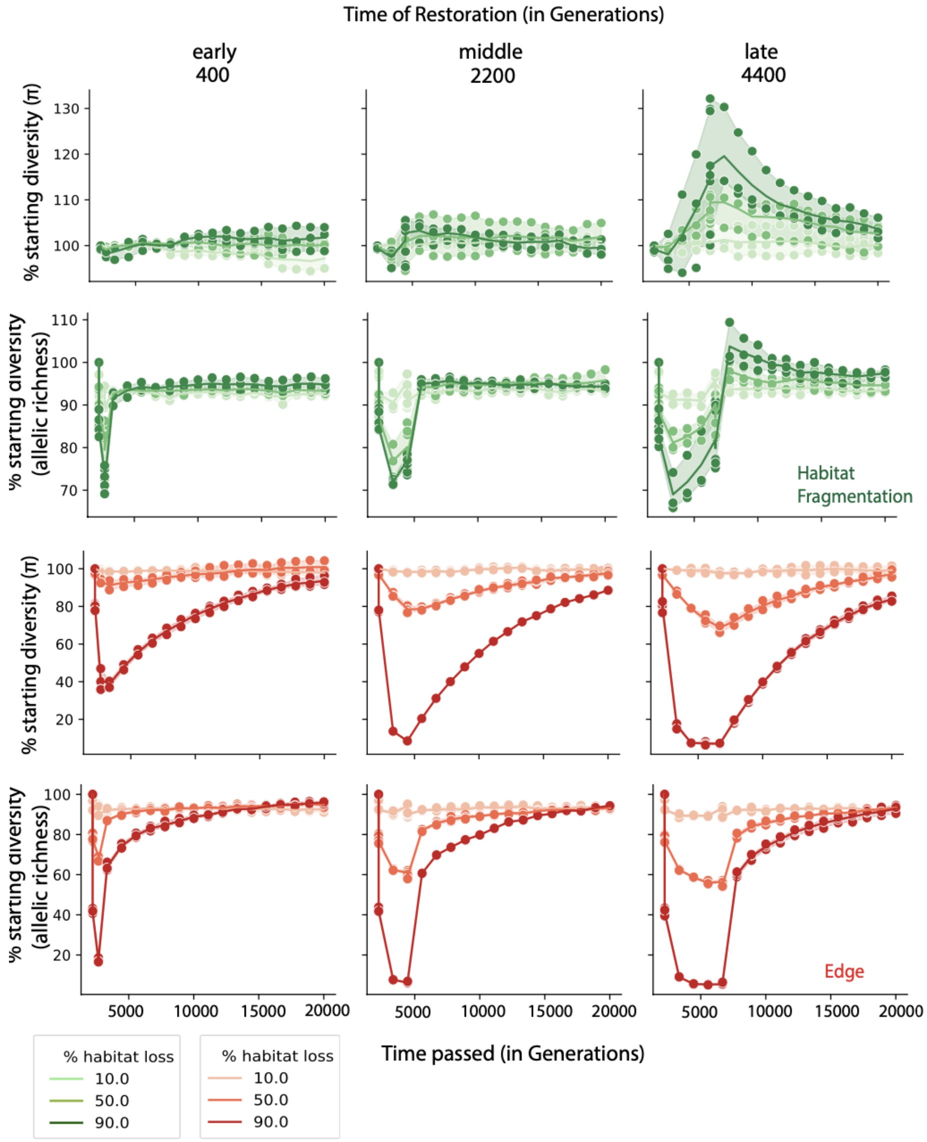

Fig. S27 | Genetic diversity metrics across habitat restoration maps

Genetic diversity metrics across habitat fragmentation (green) and edge contraction (red). Shades of green show % of habitat loss under habitat fragmentation and shades of red show % of habitat loss under edge contraction. Shown here are genetic diversity ($\pi_{species}$) metrics, % π and % allelic richness across time in generations. We track genetic diversity until the next equilibrium is reached over 40000 generations. Time of restoration is illustrated and categorized into 3 categories of early (400) , middle (2200) and late (4400). Each dot represents a genetic diversity measurement for % habitat loss at that specific time point. Shaded regions show the min-max across 3 replicates.

bioRxiv preprint doi: https://doi.org/10.1101/2024.10.21.619096; this version posted October 22, 2024. The copyright holder for this preprint (which was not certified by peer review) is the author/funder, who has granted bioRxiv a license to display the preprint in perpetuity. It is made available under aCC-BY 4.0 International license.

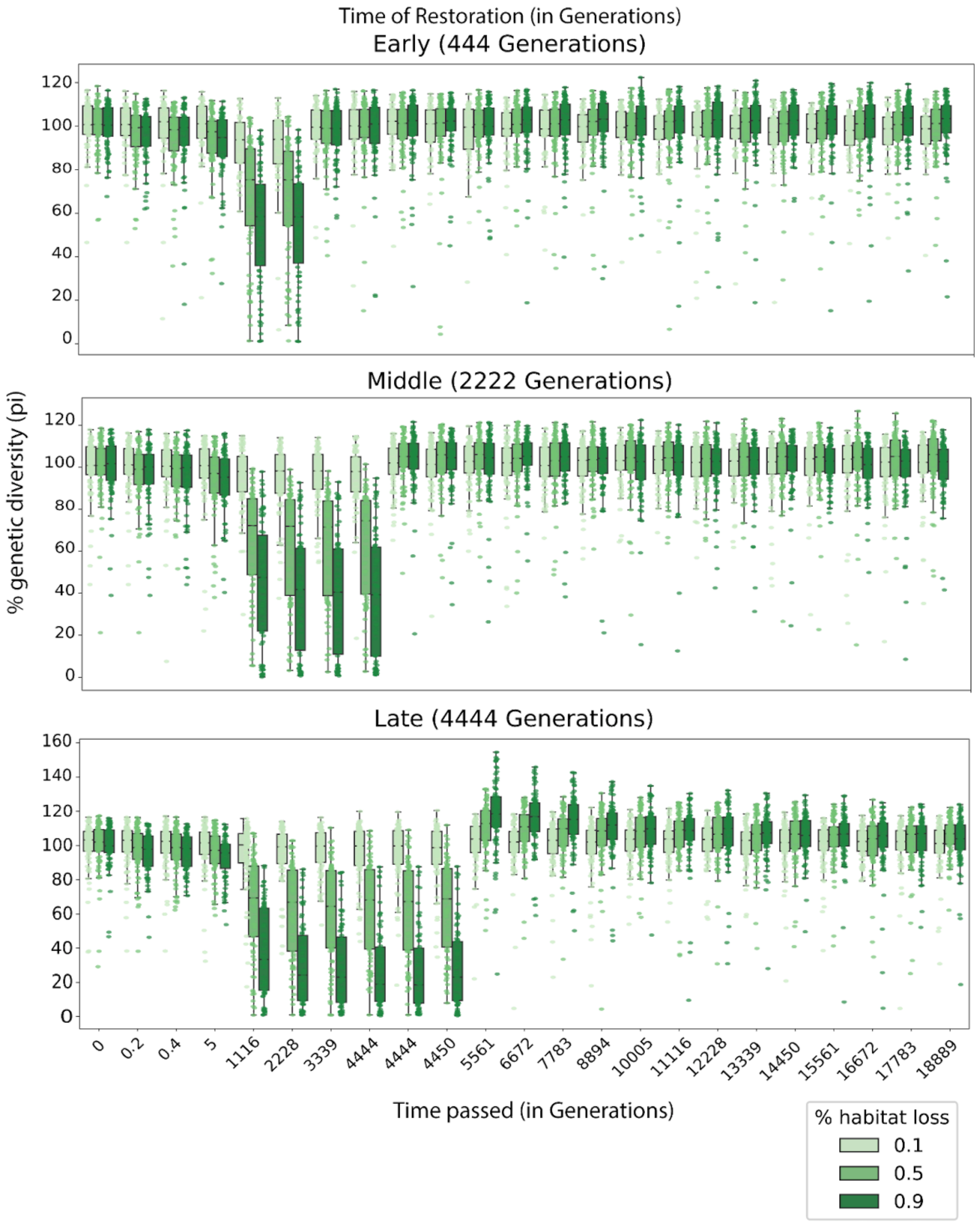

Fig. S28 | Within-population (π_{local}) genetic diversity during restoration of habitat loss with fragmentation
Genetic diversity metrics across habitat fragmentation. Shades of green show % of habitat loss under habitat fragmentation. Shown here are genetic diversity (π_{local}) metrics, % π across time in generations. We track genetic diversity until the next equilibrium is reached over ~20000 generations. Time of restoration is illustrated and categorized into 3 categories of early (400) , middle (2200) and late (4400). Each dot represents a measure of genetic diversity within a grid in a 10×10 simulation map. We performed these calculations across 3 replicates. Box and

bioRxiv preprint doi: https://doi.org/10.1101/2024.10.21.619096; this version posted October 22, 2024. The copyright holder for this preprint (which was not certified by peer review) is the author/funder, who has granted bioRxiv a license to display the preprint in perpetuity. It is made available under aCC-BY 4.0 International license.

whisker plots show the distribution of species-wide ($\pi_{species}$) over all grids in the habitat.

Supplemental Tables

Table S1 | SAR curve fit short-term genetic diversity (π) trajectories under edge contraction

We fit 20 different functions and calculated variance explained (R^2), Pearson r and Spearman ρ

Model	R^2	Pearson r	Spearman ρ
Asymptotic regression	0.949	0.974	0.976
Beta-P cumulative	0.993	0.996	0.976
Chapman Richards	0.991	0.996	0.976
Cumulative Weibull 3 par.	0.992	0.996	0.976
Cumulative Weibull 4 par.	0.993	0.996	0.976
Extended Power model 1	0.950	0.975	0.264
Extended Power model 2	0.962	0.981	0.976
Gompertz	0.920	0.959	0.976
Heleg(Logistic)	0.993	0.996	0.976
Kobayashi	0.967	0.984	0.976
Linear model	0.422	0.650	0.976
Logarithmic	0.933	0.966	0.976
Logistic(Standard)	0.900	0.949	0.976
Monod	0.993	0.996	0.976
Negative exponential	0.949	0.974	0.976
Persistence function 1	0.989	0.995	0.798
Persistence function 2	0.993	0.996	0.976
Power	0.944	0.972	0.976
PowerR	0.964	0.982	0.976
Rational function	0.993	0.996	0.976

bioRxiv preprint doi: https://doi.org/10.1101/2024.10.21.619096; this version posted October 22, 2024. The copyright holder for this preprint (which was not certified by peer review) is the author/funder, who has granted bioRxiv a license to display the preprint in perpetuity. It is made available under aCC-BY 4.0 International license.

Table S2 | SAR curve fit to long-term genetic diversity trajectories under edge contraction

We fit 20 different functions and calculated variance explained (R^2), Pearson r and Spearman ρ

Model	R^2	Pearson r	Spearman ρ
Asymptotic regression	0.942	0.971	1.000
Beta-P cumulative	0.981	0.990	1.000
Chapman Richards	0.968	0.984	1.000
Cumulative Weibull 3 par.	0.976	0.988	1.000
Cumulative Weibull 4 par.	0.983	0.992	1.000
Extended Power model 1	0.983	0.991	1.000
Extended Power model 2	0.953	0.976	1.000
Gompertz	0.932	0.965	1.000
Heleg(Logistic)	0.981	0.990	1.000
Kobayashi	0.951	0.975	1.000
Linear model	0.608	0.780	1.000
Logarithmic	0.952	0.976	1.000
Logistic(Standard)	0.923	0.961	1.000
Monod	0.967	0.983	1.000
Negative exponential	0.857	0.926	1.000
Persistence function 1	0.962	0.981	0.989
Persistence function 2	0.988	0.994	1.000
Power	0.911	0.954	1.000
PowerR	0.952	0.976	1.000
Rational function	0.765	0.875	1.000

bioRxiv preprint doi: https://doi.org/10.1101/2024.10.21.619096; this version posted October 22, 2024. The copyright holder for this preprint (which was not certified by peer review) is the author/funder, who has granted bioRxiv a license to display the preprint in perpetuity. It is made available under aCC-BY 4.0 International license.

Table S3 | SAR curve fit to short-term genetic diversity (π) theoretical trajectories under habitat fragmentation

We fit 20 different functions and calculated variance explained (R^2), Pearson r and Spearman ρ

**Model**	R^2	**Pearson r**	**Spearman ρ**
Asymptotic regression	8.61×10^{-4}	0.029	0.041
Beta-P cumulative	9.24×10^{-3}	0.096	0.105
Chapman Richards	2.58×10^{-3}	0.051	-0.041
Cumulative Weibull 3 par.	6.48×10^{-5}	-0.008	-0.041
Cumulative Weibull 4 par.	2.59×10^{-5}	-0.005	-0.041
Extended Power model 1	4.73×10^{-3}	0.069	0.055
Extended Power model 2	1.01×10^{-5}	0.003	-0.041
Gompertz	9.24×10^{-3}	0.096	0.105
Heleg(Logistic)	0.00	-0.008	-0.041
Kobayashi	7.18×10^{-5}	-0.008	-0.041
Linear model	8.58×10^{-4}	0.029	0.041
Logarithmic	7.18×10^{-5}	0.008	0.041
Logistic(Standard)	3.47×10^{-3}	0.059	0.041
Monod	1.40×10^{-4}	0.012	-0.041
Negative exponential	2.88×10^{-3}	0.054	-0.041
Persistence function 1	5.82×10^{-3}	0.076	0.072
Persistence function 2	1.40×10^{-4}	0.012	-0.041
Power	7.17×10^{-5}	0.008	0.041
PowerR	0.00	NA	NA
Rational function	1.06×10^{-4}	0.010	-0.041

bioRxiv preprint doi: https://doi.org/10.1101/2024.10.21.619096; this version posted October 22, 2024. The copyright holder for this preprint (which was not certified by peer review) is the author/funder, who has granted bioRxiv a license to display the preprint in perpetuity. It is made available under aCC-BY 4.0 International license.

Table S4 | SAR curve fit to long-term genetic diversity (π) theoretical trajectories under habitat fragmentation

We fit 20 different functions and calculated variance explained (R^2), Pearson r and Spearman ρ

Model	R^2	Pearson r	Spearman ρ
Asymptotic regression	0.493	0.702	0.620
Beta-P cumulative	0.516	0.719	0.620
Chapman Richards	0.000	NA	NA
Cumulative Weibull 3 par.	0.000	-0.229	-0.620
Cumulative Weibull 4 par.	0.000	-0.540	-0.620
Extended Power model 1	0.522	0.723	0.685
Extended Power model 2	0.263	0.513	0.620
Gompertz	0.496	0.704	0.620
Heleg(Logistic)	0.000	NA	NA
Kobayashi	0.376	-0.613	-0.620
Linear model	0.481	0.693	0.620
Logarithmic	0.376	0.613	0.620
Logistic(Standard)	0.000	NA	NA
Monod	0.000	-0.470	-0.620
Negative exponential	0.000	NA	NA
Persistence function 1	0.526	0.725	0.685
Persistence function 2	0.199	0.446	0.620
Power	0.345	0.587	0.620
PowerR	0.391	0.625	0.620
Rational function	0.444	0.666	0.620

bioRxiv preprint doi: https://doi.org/10.1101/2024.10.21.619096; this version posted October 22, 2024. The copyright holder for this preprint (which was not certified by peer review) is the author/funder, who has granted bioRxiv a license to display the preprint in perpetuity. It is made available under aCC-BY 4.0 International license.

Table S5 | SAR curve fit to within-population short-term genetic diversity (π) theory trajectories under habitat fragmentation

We fit 20 different functions and calculated variance explained (R^2), Pearson r and Spearman ρ

Model	R^2	Pearson r	Spearman ρ
Asymptotic regression	0.004	0.066	0.062
Beta-P cumulative	0.009	0.096	0.062
Chapman Richards	0.009	0.096	0.062
Cumulative Weibull 3 par.	0.009	0.096	0.062
Cumulative Weibull 4 par.	0.007	0.081	0.062
Extended Power model 1	0.009	0.096	0.062
Extended Power model 2	0.008	0.089	0.062
Gompertz	0.000	NA	NA
Heleg(Logistic)	0.009	0.096	0.062
Kobayashi	0.007	0.081	0.062
Linear model	0.004	0.066	0.062
Logarithmic	0.007	0.081	0.062
Logistic(Standard)	0.004	0.066	0.062
Monod	0.009	0.093	0.062
Negative exponential	0.007	0.082	0.062
Persistence function 1	0.009	0.096	0.068
Persistence function 2	0.009	0.094	0.062
Power	0.007	0.081	0.062
PowerR	0.009	0.096	0.062
Rational function	0.009	0.093	0.062

bioRxiv preprint doi: https://doi.org/10.1101/2024.10.21.619096; this version posted October 22, 2024. The copyright holder for this preprint (which was not certified by peer review) is the author/funder, who has granted bioRxiv a license to display the preprint in perpetuity. It is made available under aCC-BY 4.0 International license.

Table S6 | SAR curve fit to within-population long-term genetic diversity (π) trajectories under habitat fragmentation

We fit 20 different functions and calculated variance explained (R^2), Pearson r and Spearman ρ

Model	R^2	Pearson r	Spearman ρ
Asymptotic regression	0.398	0.631	0.629
Beta-P cumulative	0.419	0.647	0.629
Chapman Richards	0.418	0.647	0.629
Cumulative Weibull 3 par.	0.419	0.647	0.629
Cumulative Weibull 4 par.	0.414	0.644	0.629
Extended Power model 1	0.418	0.647	0.629
Extended Power model 2	0.389	0.623	0.629
Gompertz	0.334	0.578	0.587
Heleg(Logistic)	0.419	0.647	0.629
Kobayashi	0.386	0.621	0.629
Linear model	0.386	0.621	0.629
Logarithmic	0.393	0.627	0.629
Logistic(Standard)	0.419	0.647	0.629
Monod	0.386	0.621	0.629
Negative exponential	0.386	0.621	0.629
Persistence function 1	0.001	-0.029	-0.629
Persistence function 2	0.395	0.628	0.629
Power	0.373	0.611	0.629
PowerR	0.382	0.618	0.629
Rational function	0.397	0.630	0.629

bioRxiv preprint doi: https://doi.org/10.1101/2024.10.21.619096; this version posted October 22, 2024. The copyright holder for this preprint (which was not certified by peer review) is the author/funder, who has granted bioRxiv a license to display the preprint in perpetuity. It is made available under aCC-BY 4.0 International license.

Table S7 | SAR curve fit to within-population short-term allelic richness simulation predictions under habitat fragmentation on replicates with high connectivity

We fit 20 different functions and calculated variance explained (R^2), Pearson r and Spearman ρ

Model	R^2	Pearson r	Spearman ρ
Asymptotic regression	0.938	0.968	0.949
Beta-P cumulative	0.936	0.967	0.949
Chapman Richards	0.937	0.968	0.949
Cumulative Weibull 3 par.	0.936	0.967	0.949
Cumulative Weibull 4 par.	0.938	0.968	0.949
Extended Power model 1	0.936	0.968	0.949
Extended Power model 2	0.936	0.967	0.949
Gompertz	0.000	NA	NA
Heleg(Logistic)	0.936	0.967	0.949
Kobayashi	0.934	0.966	0.949
Linear model	0.884	0.940	0.949
Logarithmic	0.934	0.967	0.949
Logistic(Standard)	0.936	0.967	0.949
Monod	0.928	0.963	0.949
Negative exponential	0.883	0.940	0.949
Persistence function 1	0.937	0.968	0.949
Persistence function 2	0.934	0.967	0.949
Power	0.930	0.964	0.949
PowerR	0.000	NA	NA
Rational function	0.937	0.968	0.949

bioRxiv preprint doi: https://doi.org/10.1101/2024.10.21.619096; this version posted October 22, 2024. The copyright holder for this preprint (which was not certified by peer review) is the author/funder, who has granted bioRxiv a license to display the preprint in perpetuity. It is made available under aCC-BY 4.0 International license.

Table S8 | SAR curve fit to within-population long-term allelic richness simulation predictions under habitat fragmentation on replicates with high connectivity

We fit 20 different functions and calculated variance explained (R^2), Pearson r and Spearman ρ

Model	R^2	Pearson r	Spearman ρ
Asymptotic regression	0.269	0.519	0.569
Beta-P cumulative	0.264	0.514	0.569
Chapman Richards	0.269	0.519	0.569
Cumulative Weibull 3 par.	0.269	0.518	0.569
Cumulative Weibull 4 par.	0.269	0.519	0.569
Extended Power model 1	0.264	0.514	0.469
Extended Power model 2	0.232	0.481	0.569
Gompertz	0.000	NA	NA
Heleg(Logistic)	0.264	0.514	0.569
Kobayashi	0.214	0.462	0.569
Linear model	0.174	0.417	0.569
Logarithmic	0.214	0.462	0.569
Logistic(Standard)	0.174	0.417	0.569
Monod	0.245	0.495	0.569
Negative exponential	0.268	0.518	0.569
Persistence function 1	0.246	0.496	0.486
Persistence function 2	0.247	0.497	0.569
Power	0.211	0.460	0.569
PowerR	0.263	0.513	0.569
Rational function	0.247	0.497	0.569

bioRxiv preprint doi: https://doi.org/10.1101/2024.10.21.619096; this version posted October 22, 2024. The copyright holder for this preprint (which was not certified by peer review) is the author/funder, who has granted bioRxiv a license to display the preprint in perpetuity. It is made available under aCC-BY 4.0 International license.

Table S9 | SAR curve fit to within-population short-term genetic diversity π under habitat fragmentation on replicates with high connectivity

We fit 20 different functions and calculated variance explained (R^2), Pearson r and Spearman ρ

Model	R^2	Pearson r	Spearman ρ
Asymptotic regression	0.931	0.965	0.749
Beta-P cumulative	0.932	0.966	0.749
Chapman Richards	0.881	0.939	0.749
Cumulative Weibull 3 par.	0.932	0.966	0.749
Cumulative Weibull 4 par.	0.932	0.966	0.749
Extended Power model 1	0.932	0.966	0.749
Extended Power model 2	0.906	0.952	0.749
Gompertz	0.000	NA	NA
Heleg(Logistic)	0.932	0.966	0.749
Kobayashi	0.782	0.884	0.749
Linear model	0.458	0.677	0.749
Logarithmic	0.782	0.884	0.749
Logistic(Standard)	0.881	0.939	0.749
Monod	0.932	0.965	0.749
Negative exponential	0.881	0.939	0.749
Persistence function 1	0.848	0.921	0.749
Persistence function 2	0.932	0.965	0.749
Power	0.776	0.881	0.749
PowerR	0.000	NA	NA
Rational function	0.931	0.965	0.749

bioRxiv preprint doi: https://doi.org/10.1101/2024.10.21.619096; this version posted October 22, 2024. The copyright holder for this preprint (which was not certified by peer review) is the author/funder, who has granted bioRxiv a license to display the preprint in perpetuity. It is made available under aCC-BY 4.0 International license.

Table S10 | SAR curve fit to within-population long-term genetic diversity π simulation under habitat fragmentation on replicates with high connectivity

We fit 20 different functions and calculated variance explained (R^2), Pearson r and Spearman ρ

Model	R^2	Pearson r	Spearman ρ
Asymptotic regression	0.365	0.604	0.749
Beta-P cumulative	0.383	0.619	0.749
Chapman Richards	0.000	NA	NA
Cumulative Weibull 3 par.	0.000	-0.261	-0.749
Cumulative Weibull 4 par.	0.000	-0.202	-0.749
Extended Power model 1	0.018	0.132	0.749
Extended Power model 2	0.098	0.313	0.749
Gompertz	0.000	NA	NA
Heleg(Logistic)	0.000	NA	NA
Kobayashi	0.203	-0.451	-0.749
Linear model	0.326	0.571	0.749
Logarithmic	0.203	0.451	0.749
Logistic(Standard)	0.000	NA	NA
Monod	0.000	-0.266	-0.749
Negative exponential	0.000	NA	NA
Persistence function 1	0.367	0.606	0.745
Persistence function 2	0.063	0.251	0.749
Power	0.175	0.418	0.749
PowerR	0.348	0.590	0.749
Rational function	0.283	0.532	0.749

bioRxiv preprint doi: https://doi.org/10.1101/2024.10.21.619096; this version posted October 22, 2024. The copyright holder for this preprint (which was not certified by peer review) is the author/funder, who has granted bioRxiv a license to display the preprint in perpetuity. It is made available under aCC-BY 4.0 International license.

Table S11 | SAR curve fit to within-population short-term genetic diversity π in larger 20x20 habitat maps under habitat fragmentation

We fit 20 different functions and calculated variance explained (R^2), Pearson r and Spearman ρ

Model	R^2	Pearson r	Spearman ρ
Asymptotic regression	0.019	0.137	0.068
Beta-P cumulative	0.018	0.135	0.068
Chapman Richards	0.019	0.137	0.068
Cumulative Weibull 3 par.	0.018	0.136	0.068
Cumulative Weibull 4 par.	0.018	0.136	0.068
Extended Power model 1	0.019	0.137	0.080
Extended Power model 2	0.017	0.130	0.068
Gompertz	0.000	NA	NA
Heleg(Logistic)	0.018	0.135	0.068
Kobayashi	0.014	0.120	0.068
Linear model	0.009	0.093	0.068
Logarithmic	0.014	0.120	0.068
Logistic(Standard)	0.014	0.117	0.105
Monod	0.018	0.134	0.068
Negative exponential	0.015	0.123	0.068
Persistence function 1	0.020	0.141	0.092
Persistence function 2	0.018	0.134	0.068
Power	0.014	0.119	0.068
PowerR	0.000	NA	NA
Rational function	0.018	0.134	0.068

bioRxiv preprint doi: https://doi.org/10.1101/2024.10.21.619096; this version posted October 22, 2024. The copyright holder for this preprint (which was not certified by peer review) is the author/funder, who has granted bioRxiv a license to display the preprint in perpetuity. It is made available under aCC-BY 4.0 International license.

Table S12 | SAR curve fit to within-population long-term genetic diversity π in larger 20 x 20 habitat maps under habitat fragmentation

We fit 20 different functions and calculated variance explained (R^2), Pearson r and Spearman ρ

Model	R^2	Pearson r	Spearman ρ
Asymptotic regression	0.273	0.523	0.600
Beta-P cumulative	0.277	0.526	0.600
Chapman Richards	0.276	0.525	0.600
Cumulative Weibull 3 par.	0.277	0.526	0.600
Cumulative Weibull 4 par.	0.278	0.527	0.600
Extended Power model 1	0.274	0.523	0.600
Extended Power model 2	0.273	0.523	0.600
Gompertz	0.245	0.495	0.584
Heleg(Logistic)	0.275	0.524	0.600
Kobayashi	0.271	0.521	0.600
Linear model	0.265	0.514	0.600
Logarithmic	0.262	0.512	0.600
Logistic(Standard)	0.281	0.530	0.600
Monod	0.272	0.521	0.600
Negative exponential	0.272	0.522	0.600
Persistence function 1	0.278	0.527	0.600
Persistence function 2	0.272	0.521	0.600
Power	0.269	0.518	0.600
PowerR	0.271	0.520	0.600
Rational function	0.273	0.522	0.600

bioRxiv preprint doi: https://doi.org/10.1101/2024.10.21.619096; this version posted October 22, 2024. The copyright holder for this preprint (which was not certified by peer review) is the author/funder, who has granted bioRxiv a license to display the preprint in perpetuity. It is made available under aCC-BY 4.0 International license.

Table S13 | Genetic diversity and area relationship summaries for different landscapes and metrics
Fitted values in a log-log power law function between habitable area and genetic diversity (π)

Top model	R^2	Parameters	Time point	Landscape	Genetic metric	Table
$\log_{10}(\pi) = c \times \log_{10}(A)^z$	0.929	c=2.05, z=0.03	Short-term	Edge contraction	π species	Table S1
$\log_{10}(\pi) = c \times \log_{10}(A)^z$	0.873	c=2.00, z=0.15	Long-term	Edge contraction	π species	Table S2
$\pi = d * (1 - (1 + (A/c)^z)^{-f})$	0.009	d=102 c=80.5 z=-37.0 f=328	Short-term	Habitat fragmentation	π species	Table S3
$\pi = c \times A^z \times \exp(-d \times A)$	0.526	c=102 z=0.322 d=0.017	Long-term	Habitat fragmentation	π species	Table S4
$\pi = f + c \times A^z$	0.009	f=84.5 c=-210 z=-1.46	Short-term	Habitat fragmentation	π within population	Table S5
$\pi = d * (1 - (1 + (A/c)^z)^{-f})$	0.419	d=78.5 c=125 z=4.73 f=38	Long-term	Habitat fragmentation	π within population	Table S6
$M = d \times (1 - \exp(-c \times A^z))^f$	0.937	d=96 $c=9.27 \times 10^{-7}$ z=3.44 $f=6.78 \times 10^{-2}$	Short-term	Habitat fragmentation high connectivity	Allelic richness (M) within population	Table S7
$M = d \times (1 - \exp(-c \times A^z))^f$	0.269	d=86 c=0.12 z=1.12 f=0.65	Long-term	Habitat fragmentation high connectivity	Allelic richness (M) within population	Table S8
$\pi = c \times A^{(z \times A\text{-}d)}$	0.932	c=100 z=-0.73 d=1.46	Short-term	Habitat fragmentation high connectivity	π within population	Table S9
$\pi = d - c \times z^A$	0.365	d=146 c=4.40 z=1.03	Long-term	Habitat fragmentation high connectivity	π within population	Table S10
$\pi = c \times A^z \times \exp(-d \times A)$	0.0199	c=101 $z=9.99 \times 10^{-2}$ $d=1.73 \times 10^{-3}$	Short-term	Habitat fragmentation 20x20	π within population	Table S11
$\pi = d \times (1 - \exp(-c \times A^z))^f$	0.278	d=140 $c=2.11 \times 10^{-6}$ z=3.09 f=0.32	Long-term	Habitat fragmentation 20x20	π within population	Table S12

bioRxiv preprint doi: https://doi.org/10.1101/2024.10.21.619096; this version posted October 22, 2024. The copyright holder for this preprint (which was not certified by peer review) is the author/funder, who has granted bioRxiv a license to display the preprint in perpetuity. It is made available under aCC-BY 4.0 International license.

Table S14 | Genetic diversity and habitable area fit with 18 empirical species

Fitted values in a log-log power law function between area and genetic diversity (π) across short-term empirical simulations in 15 species. We fit the power law functions for short-term **(Table S7)** and calculate variance explained (R^2), Pearson r and Spearman rho.

species	Number of samples	R2 (mean)	R2 (95% CI)	pearson r (mean)	pearson r (95% CI)	spearman r (mean)	spearman r (95% CI)
Acropora millepora	253 (12)[*]	0.986	[0.985, 0.986]	0.993	[0.993, 0.994]	0.171	[0.134, 0.207]
Arabidopsis lyrata	108	0.972	[0.971, 0.973]	0.994	[0.994, 0.995]	0.982	[0.976, 0.987]
Amaranthus tuberculatus	162 (155)	0.987	[0.9871, 0.9873]	0.994	[0.994, 0.994]	0.355	[0.281, 0.429]
Arabidopsis thaliana	1,135 (1,001)[#]	0.864	[0.858, 0.870]	0.944	[0.942, 0.946]	-0.919	[-0.928, -0.911]
Drosophila melanogaster	271	0.169	[0.166, 0.173]	0.783	[0.783, 0.784]	0.916	[0.905, 0.928]
Eucalyptus melliodora	275 (36)[*]	0.966	[0.963, 0.969]	0.985	[0.984, 0.986]	0.634	[0.538, 0.729]
Yucca brevifolia	290	0.221	[0.218, 0.224]	0.759	[0.758, 0.760]	0.587	[0.514, 0.661]
Mimulus guttatus	521 (286)[#*]	0.925	[0.923, 0.927]	0.962	[0.961, 0.963]	0.735	[0.712, 0.758]
Anopheles gambiae	1142 (29)[*]	0.109	[0.109, 0.110]	0.74	[0.739, 0.740]	0.516	[0.432, 0.601]
Panicum hallii	591	0.914	[0.911, 0.916]	0.961	[0.960, 0.962]	-0.735	[-0.763, -0.706]
Panicum virgatum	732 (576)[†]	0.258	[0.239, 0.276]	0.831	[0.829, 0.833]	-0.077	[-0.097, -0.057]
Peromyscus maniculatus	80 (78)[&]	0.134	[0.134, 0.135]	0.746	[0.746 0.747]	0.61	[0.558, 0.662]
Populus trichocarpa	882	0.941	[0.940, 0.942]	0.974	[0.974, 0.975]	-0.448	[-0.493 -0.403]
Dicerorhinus sumatrensis	16	0.076	[0.075, 0.076]	0.73	[0.729, 0.730]	0.19	[0.143, 0.237]
Empidonax traillii	219 (199)[&]	0.94	[0.932, 0.948]	0.975	[0.972, 0.978]	-0.524	[-0.596, -0.453]
Pinus torreyana	242	0.997	[0.996, 0.997]	0.999	[0.999 0.999]	1	[nan nan]
Setophaga petechia	199	0.975	[0.974, 0.975]	0.988	[0.988, 0.988]	0.892	[0.874, 0.909]
Canis lupus	349 (230)[‡]	0.994	[0.993, 0.995]	1	[0.999, 1.0]	1	[nan nan]

[#]*Only individuals in the native range were used for the analyses.*
[&]*Only individuals with available coordinates or matching IDs were used for analyses.*
[*]*Number of geographically separated populations, as multiple individuals were collected per population.*
[†]*Only natural populations were used, excluding breeds, landraces, and cultivars.*

bioRxiv preprint doi: https://doi.org/10.1101/2024.10.21.619096; this version posted October 22, 2024. The copyright holder for this preprint (which was not certified by peer review) is the author/funder, who has granted bioRxiv a license to display the preprint in perpetuity. It is made available under aCC-BY 4.0 International license.

Table S15 | F_{ST} values across diverse species

F_{ST} values calculated using admixture R package [48] to estimate the most likely number of populations (K) tracked. z_{GDAR} values using SAR R package [61] following the calculation of z_{MAR} values in [8].

Species	Publication	F_{ST} avg	F_{ST} max	z_{GDAR}	z_{MAR}
Arabidopsis thaliana	10.1016/j.cell.2016.05.063	0.3032	0.552	0.002	0.324
Arabidopsis lyrata	10.1371/journal.pgen.1009477	0.137	0.137	0.018	0.236
Setophaga petechia	10.1126/science.aan4380	0.054	0.054	0.011	0.178
Pinus torreyana	10.1093/aobpla/plab058	0.014	0.014	0.027	0.142
Populus trichocarpa	10.13139/OLCF/1411410	0.101	0.179	0.006	0.275
Drosophila melanogaster	10.1093/molbev/msab259	0.316	0.505	0.034	0.437
Peromyscus maniculatus	10.1111/evo.13150	0.087	0.087	0.037	0.488
Panicum hallii	10.1038/s41586-020-03127-1	0.238	0.369	0.455	0.824
Panicum virgatum	10.1111/mec.13467	0.530	0.820	0.009	0.232
Empidonax traillii	10.1111/gcb.15639	0.616	0.974	0.034	0.214
Canis lupus	10.1111/mec.13364	0.092	0.092	0.064	0.256
Yucca brevifolia	10.3732/ajb.1600069	0.395	0.716	0.018	×0.128
Mimulus guttatus	10.1038/s42003-021-01795-x	0.372	0.613	0.018	0.274
Dicerorhinus sumatrensis	10.1038/s41467-021-22386-8	0.690	0.870	-0.005	0.412
Amaranthus tuberculatus	10.1073/pnas.190087011	0.273	0.497	-0.002	0.109
Anopheles gambiae	10.1038/nature24995	0.321	0.484	0.044	0.214
Acropora millepora	10.1126/science.aba4674	0.105	0.105	0.006	0.246
Eucalyptus melliodora	10.7554/eLife.31835	0.010	0.010	0.009	0.466

Abbreviations. ✕Values excluded from global averages used for conservation applications due to uncertain estimates, suboptimal genomic data type.

bioRxiv preprint doi: https://doi.org/10.1101/2024.10.21.619096; this version posted October 22, 2024. The copyright holder for this preprint (which was not certified by peer review) is the author/funder, who has granted bioRxiv a license to display the preprint in perpetuity. It is made available under aCC-BY 4.0 International license.

Table S16 | IUCN Red List area and population criteria for 80 thousand species

Each species was parsed for the indicator used to be classified in a given category. Summary of Red List database (www.iucnredlist.org). Counts of each category as well as criteria used in their classification are summarized (for details see extended guidelines). Area loss is obtained by using the Red List criteria for each category. Estimates of short and long-term genetic diversity loss were calculated using our theoretical and simulation-based framework.

	**Population size loss**		**Small geographic range**		**Small pop. + decline**		**Last adults**	**Quant. model**	**In decline (% # spp)**	**# species**		**Area lost (%) [A1-A2_4]**	**short-term theory (%)**	**long-term theory (%)**
Category:	A1 (past)	A2-4 (now+fut)	B1 (Extent occurrence)	B2 (Area of occupancy)	C1	C2	D	E						
**Extinct**	-	-	-	-	-		-	-	-	**452**	0.5 %	98 %	99.94 %	98.02 %
**Likely extinct**	0	51	385	472	5	40	236	0	47.70%	**782**	0.9 %	98 %	99.94 %	98.02 %
	≥90% loss	*≥80% loss*	*<100 km2*	*<10 km2*	*≥25% loss*	*<50 ind.*	*<50 ind.*	*P(E)≥50%*						
Critically endangered	136	916	3096	2765	147	798	897	0	61.50%	5339	6.4 %	87.5 %	99.6 %	87.62 %
	≥70% loss	*≥50% loss*	*<5,000 km2*	*<500 km2*	*≥20% loss*	*<250 ind.*	*<250 ind.*	*P(E)≥20%*						
Endangered	142	1621	56895	7193	173	577	411	0	63.10%	11475	13.9 %	64.5 %	98.69 %	64.78 %
	≥50% loss	*≥30% loss*	*<20,000 km2*	*<2,000 km2*	*≥10% loss*	*<1,000 ind.*	*<1,000 ind.*	*P(E)≥10%*						
Vulnerable	492	2240	3976	4138	167	425	0	0	44.80%	11001	13.3 %	39.5 %	97.25 %	39.87 %
Near Threatened	28	1688	1201	1397	154	326	0	0	49.8%	5292	6.4 %	22.5 %	95.62 %	22.84 %
Least Concern	0	0	0	0	0		0	0	15.00%	48460	58.5 %	9.5 %	93.18 %	9.73 %
**Total eval.**										**82798**	**100 %**			

# Note one species can be categorized based on multiple impacts or criteria, so the total is not the sum of cell values.

bioRxiv preprint doi: https://doi.org/10.1101/2024.10.21.619096; this version posted October 22, 2024. The copyright holder for this preprint (which was not certified by peer review) is the author/funder, who has granted bioRxiv a license to display the preprint in perpetuity. It is made available under aCC-BY 4.0 International license.

Mathematical Appendix: Expected genetic diversity dynamics under the Wright-Fisher Diffusion

Derivation of expected genetic diversity dynamics

In this section we describe our theoretical model and the computational machinery we use to describe the dynamics of genetic diversity loss. Our starting point is the multi-deme, single locus, biallelic Wright-Fisher diffusion [1], which models the evolution of a pair of alleles at a single locus. Throughout, we focus on the dynamics of alleles that are not under selection, but this model captures the effects of genetic drift, population substructure, and migration.

In particular, we consider D demes with a population scaled mutation rate $\theta/2$, relative population size of deme k, η_k, and migration matrix $\mathbf{M}$, with $\mathbf{M}_{ij}$ being the population-scaled migration rate from deme i to deme j, and $\mathbf{M}_{jj} = -\sum_{i\neq j}\mathbf{M}_{ij}$. Note that in this parameterization, the *columns* pf $\mathbf{M}$ sum to zero, and entry $\mathbf{M}_{ij}$ represents the rate at which individuals in deme j are replaced by individuals from deme i. This is sometimes called the "backward migration rate" as it represents the rate at which lineages in deme j migrate to deme i backward in time in a coalescent framework. This is in contrast to a "forward migration rate" which could be interpreted as a number individuals per generation that move from one deme to another. In any case, we model the dynamics of the allele frequency in deme k at time t, $X_t^{(k)}$, via the following Stochastic Differential Equation (SDE):

$$\underbrace{\mathrm{d}X_t^{(k)}}_{\text{infinitesimal allele frequency change}} = \left\{\underbrace{\frac{\theta}{2}\left(1-2X_t^{(k)}\right)}_{\text{Mutation}} + \underbrace{\sum_{i=1}^{D}\mathbf{M}_{ik}X_t^{(i)}}_{\text{Migration}}\right\}\mathrm{d}t + \underbrace{\sqrt{\frac{X_t^{(k)}(1-X_t^{(k)})}{\eta_k}}\mathrm{d}W_t^{(k)}}_{\text{Genetic drift}} \tag{1}$$

where $W^{(1)},\ldots,W^{(D)}$ are independent Wiener Processes (Brownian Motions).

Our goal is to understand how π, the nucleotide diversity, evolves over time. We will write $\pi(t)$ throughout to emphasize that we are thinking of π as a dynamical object that can change over time. Mathematically, $\pi(t)$ is the probability that two individuals chosen at random at time t have different alleles, and $\pi(t)$ is always defined with respect to a given population. For example, within a single deme, say deme k, $\pi(t)$ is

$$\pi(t) = 2X_t^{(k)}(1-X_t^{(k)}).$$

bioRxiv preprint doi: https://doi.org/10.1101/2024.10.21.619096; this version posted October 22, 2024. The copyright holder for this preprint (which was not certified by peer review) is the author/funder, who has granted bioRxiv a license to display the preprint in perpetuity. It is made available under aCC-BY 4.0 International license.

If, instead we draw individuals uniformly at random across all demes, we instead obtain

$$\pi(t) = \frac{2}{D^2} \sum_{i=1}^{D} \sum_{j=1}^{D} X_t^{(i)} \left(1 - X_t^{(j)}\right).$$

We are interested in π because it can be estimated by sequencing at least two haploids, and hence can be tied to observable data and tracked over time via sequencing. Furthermore, it is common to estimate an average π, by averaging the nucleotide diversity at each site across the entire genome. Since we are averaging over many sites, we can estimate the average π quite well, and hence we may want to compare this average to the theoretical average value of π,

$$\mathbb{E}[\pi(t)] = 2\mathbb{E}\left[X_t^{(k)}(1 - X_t^{(k)})\right] \tag{2}$$

in the single deme case, or

$$\mathbb{E}[\pi(t)] = \frac{2}{D^2} \sum_{i=1}^{D} \sum_{j=1}^{D} \mathbb{E}\left[X_t^{(i)} \left(1 - X_t^{(j)}\right)\right] \tag{3}$$

in the case of multiple demes.

As can be seen in eqs. (2) and (3), the expected value of $\pi(t)$ is a function of only first and second moments of the allele frequencies in each deme at time t. That is, only terms like $\mathbb{E}\left[X_t^{(k)}\right]$ and $\mathbb{E}\left[X_t^{(i)} X_t^{(j)}\right]$ appear in eqs. (2) and (3). If we could compute these moments of the allele frequency through time, then we would be able to track the dynamics of $\mathbb{E}[\pi(t)]$. The remainder of this section is dedicated to computing these moments.

To begin, we can consider taking the expectation of the left and right sides of eq. (1) and interchange expectation with the stochastic differentials:

$$\begin{aligned}\frac{\mathrm{d}}{\mathrm{d}t}\mathbb{E}\left[X_t^{(k)}\right] &= \frac{\theta}{2}\left(1 - 2\mathbb{E}\left[X_t^{(k)}\right]\right) + \sum_{i=1}^{D} \mathbf{M}_{ik}\mathbb{E}\left[X_t^{(i)}\right] + \sqrt{\frac{\mathbb{E}\left[X_t^{(k)}(1 - X_t^{(k)})\right]}{\eta_k}}\frac{\mathrm{d}}{\mathrm{d}t}\mathbb{E}\left[W_t^{(k)}\right] \\ &= \frac{\theta}{2}\left(1 - 2\mathbb{E}\left[X_t^{(k)}\right]\right) + \sum_{i=1}^{D} \mathbf{M}_{ik}\mathbb{E}\left[X_t^{(i)}\right] \end{aligned} \tag{4}$$

where the second line follows from the fact $W_t^{(k)}$ is a martingale so its expected value is constant over time. Note that while this argument is far from rigorous, it can be made fully rigorous using Dynkin's Formula (see e.g., [2, Theorem 7.4.1]).

Note that the right hand side of eq. (4) only depends on the first moments of the allele frequencies in the demes. This means, that we can track these first moments via this system of ordinary differential equations (ODEs). Furthermore, this system is linear, making it particularly easy to solve in general. We note however, that setting $\mathbf{E}[X_t^{(1)}] = \cdots = \mathbf{E}[X_t^{(D)}] = 1/2$ is an equilibrium solution as in that case, which can easily be seen by plugging $1/2$ in for all first moments in eq. (4) and recalling that the columns of $\mathbf{M}$ sum to zero by construction. Then, once this equilibrium state has been reached, the right hand side of eq. (4) remains zero even if the patterns of migration

bioRxiv preprint doi: https://doi.org/10.1101/2024.10.21.619096; this version posted October 22, 2024. The copyright holder for this preprint (which was not certified by peer review) is the author/funder, who has granted bioRxiv a license to display the preprint in perpetuity. It is made available under aCC-BY 4.0 International license.

change or the mutation rate changes. As a result, for the remainder of this section we will assume that $\mathbb{E}[X_t^{(1)}] = \cdots = \mathbf{E}[X_t^{(D)}] = 1/2$ for all time.

Having tackled the first moments, we turn to the second moments of the form $\mathbb{E}[X_t^{(i)} X_t^{(j)}]$. Here we rely on a special case of the multivariate form of Itô's Lemma (see e.g., [2, Theore 4.2.1]), which considers a multivariate SDE for $Y_t = (Y_t^{(1)}, \ldots, Y_t^{(D)})$ with drift term $\mu(Y_t) = (\mu^{(1)}(Y_t), \ldots, \mu^{(D)}(Y_t))$ and stochastic term $\sigma(Y_t) = (\sigma^{(1)}(Y_t), \ldots, \sigma^{(D)}(Y_t))$. That is,

$$\mathrm{d}Y_t^{(k)} = \mu^{(k)}(Y_t)\mathrm{d}t + \sigma^{(k)}(Y_t)\mathrm{d}W_t^{(k)}.$$

For such an SDE, Itô's Lemma allows us to derive the SDE for any scalar function of Y_t via

$$\mathrm{d}f(Y_t) = \sum_{k=1}^{D} \left[\mu^{(k)}(Y_t)\frac{\partial f}{\partial y_k}(Y_t) + \frac{\sigma^{(k)}(Y_t)^2}{2}\frac{\partial^2 f}{\partial y_k^2}(Y_t)\right] \mathrm{d}t + \sigma^{(k)}(Y_t)\frac{\partial f}{\partial y_k}(Y_t)\mathrm{d}W_t^{(k)}. \tag{5}$$

Returning to the case of second moments, we may consider the function

$$f(x_1, x_2, \ldots, x_d) = x_i x_j$$

and apply eq. (5) with the SDE in eq. (1) to obtain (after much simplification)

$$\begin{aligned} \mathrm{d}\left(X_t^{(i)} X_t^{(j)}\right) = \Bigg[&\frac{\theta}{2}\left(X_t^{(i)} + X_t^{(j)} - 4X_t^{(i)}X_t^{(j)}\right) \\ &+ \delta_{i,j}\frac{X_t^{(i)}(1 - X_t^{(i)})}{\eta_i} \\ &+ \sum_{k=1}^{D} \mathbf{M}_{ki} X_t^{(k)} X_t^{(j)} + \mathbf{M}_{kj} X_t^{(k)} X_t^{(i)}\Bigg]\mathrm{d}t \\ &+ X_t^{(j)}\sqrt{\frac{X_t^{(i)}(1 - X_t^{(i)})}{\eta_i}}\mathrm{d}W_t^{(i)} + X_t^{(i)}\sqrt{\frac{X_t^{(j)}(1 - X_t^{(j)})}{\eta_j}}\mathrm{d}W_t^{(j)} \end{aligned} \tag{6}$$

where $\delta_{i,j}$ is the Kronecker delta with $\delta_{i,j} = 1$ if $i = j$ and $\delta_{i,j} = 0$ otherwise. Taking expectations of both sides of eq. (6) (i.e., applying Dynkin's Formula), and recalling that we take $\mathbb{E}[X_t^{(k)}] = 1/2$ for all t and all k we obtain the following ODE:

$$\begin{aligned} \frac{\mathrm{d}}{\mathrm{d}t}\mathbb{E}\left[X_t^{(i)} X_t^{(j)}\right] = &\frac{\theta}{2} + \frac{\delta_{i,j}}{2\eta_i} - \left(2\theta + \frac{\delta_{i,j}}{\eta_i}\right)\mathbb{E}\left[X_t^{(i)} X_t^{(j)}\right] \\ &+ \sum_{k=1}^{D} \mathbf{M}_{ki}\mathbb{E}\left[X_t^{(k)} X_t^{(j)}\right] + \mathbf{M}_{kj}\mathbb{E}\left[X_t^{(k)} X_t^{(i)}\right]. \end{aligned} \tag{7}$$

As in the first moment case, if we consider all of the second moments, then the above constitutes a linear system of ODEs that only depends on the second moments.

Given some initial condition, we can then use 7 to determine the dynamics of the second moments through time. Since these second moments determine $\mathbb{E}[\pi(t)]$ according to eqs. (2) and (3), the dynamics of the second moments fully describe the dynamics of the expected genetic diversity.

bioRxiv preprint doi: https://doi.org/10.1101/2024.10.21.619096; this version posted October 22, 2024. The copyright holder for this preprint (which was not certified by peer review) is the author/funder, who has granted bioRxiv a license to display the preprint in perpetuity. It is made available under aCC-BY 4.0 International license.

In the next subsection, we will discuss the initial condition, along with computational considerations in more detail.

Finally, we note that since we have compute all of the second moments of the allele frequencies across all demes, we can compute any parameter that depends only on the first two moments, not just π. For example, the fixation index, F_{st}, between two populations, i and j, can either be defined as

$$F_{st}^{\text{(Nei)}} = 1 - \frac{\mathbb{E}\left[X_t^{(i)}\left(1 - X_t^{(i)}\right) + X_t^{(j)}\left(1 - X_t^{(j)}\right)\right]}{\mathbb{E}\left[\left(X_t^{(i)} + X_t^{(j)}\right)\left(1 - \frac{1}{2}\left(X_t^{(i)} + X_t^{(j)}\right)\right)\right]}$$

or

$$F_{st}^{\text{(Hudson)}} = 1 - \frac{\mathbb{E}\left[X_t^{(i)}\left(1 - X_t^{(i)}\right) + X_t^{(j)}\left(1 - X_t^{(j)}\right)\right]}{\mathbb{E}\left[X_t^{(i)}\left(1 - X_t^{(j)}\right) + X_t^{(j)}\left(1 - X_t^{(i)}\right)\right]}$$

and since our machinery can compute all of the involved moments, we can easily compute these values under arbitrary neutral multi-deme models. See [3] or [4] for more details about the various non-equivalent definitions of F_{st}.

Computational Considerations

While eqs. (2), (3) and (7) together describe the dynamics of $\mathbb{E}[\pi(t)]$ in theory, to obtain numerical results we must first determine an initial value of of the second moments, and then must solve the system of ODEs in eq. (7) to track the dynamics. In this subsection, we discuss these computational considerations. Our approach is implemented as a python API in the package `wfmoments` available at `https://github.com/jeffspence/wfmoments`.

The second moments are naturally indexed by the corresponding demes, for example, it would be natural to describe $\mathbb{E}\left[X_t^{(i)}X_t^{(j)}\right]$ as the i, j^{th} second moment. And indeed, it is natural to think of the second moments as forming a $D \times D$ matrix. Yet, eq. (7) is merely linear in these moments, and so the matrix structure implied by having two indices for each second moment is unnecessary. Furthermore, the moments are invariant to the ordering of the pairs of indices as $\mathbb{E}\left[X_t^{(i)}X_t^{(j)}\right] = \mathbb{E}\left[X_t^{(j)}X_t^{(i)}\right]$. Instead, we can consider flattening this matrix of second moments, and keeping only one copy of either the $(i, j)^{\text{th}}$ or $(j, i)^{\text{th}}$ moment. This results in a vector of length $D(D + 1)/2$. We will write $\mathbf{m}_t$ for this moment vector. The precise way in which the matrix is flattened into this vector is unimportant, so long as the way that pairs of indices in $\{1, \ldots, D\} \times \{1, \ldots, D\}$ get mapped to indices in $\{1, \ldots, D(D + 1)/2\}$ is consistent. For examples we could consider a row-first mapping to define

$$\mathbf{m}_t := \left(\mathbb{E}\left[X_t^{(1)}X_t^{(1)}\right], \mathbb{E}\left[X_t^{(1)}X_t^{(2)}\right], \ldots, \mathbb{E}\left[X_t^{(1)}X_t^{(D)}\right], \mathbb{E}\left[X_t^{(2)}X_t^{(2)}\right], \mathbb{E}\left[X_t^{(2)}X_t^{(3)}\right], \ldots\right).$$

Throughout, we will write $\iota(\cdot, \cdot)$ as this index mapping. In particular, $\iota(i, j) = \iota(j, i)$ and entry $\iota(i, j)$ of $\mathbf{m}_t$ is the moment $\mathbf{E}\left[X_t^{(i)}X_t^{(j)}\right]$.

We can then write the system of ODEs in eq. (7) as

$$\frac{\mathrm{d}}{\mathrm{d}t}\mathbf{m}_t = \mathbf{v} + \mathbf{G}\mathbf{m}_t \tag{8}$$

bioRxiv preprint doi: https://doi.org/10.1101/2024.10.21.619096; this version posted October 22, 2024. The copyright holder for this preprint (which was not certified by peer review) is the author/funder, who has granted bioRxiv a license to display the preprint in perpetuity. It is made available under aCC-BY 4.0 International license.

for a matrix $\mathbf{G} \in \mathbb{R}^{D(D+1)/2 \times D(D+1)/2}$ that has entries

$$\begin{aligned}\mathbf{G}_{\iota(i,j),\iota(k,\ell)} = &- \delta_{\iota(i,j),\iota(k,\ell)}\theta \\ &- \delta_{i,j}\delta_{\iota(i,j),\iota(k,\ell)}\frac{1}{\eta_i} \\ &+ \delta_{i,k}\mathbf{M}_{\ell,j} \\ &+ \delta_{j,k}\mathbf{M}_{\ell,i} \\ &+ \delta_{i,\ell}(1-\delta_{k,\ell})\mathbf{M}_{k,j} \\ &+ \delta_{j,\ell}(1-\delta_{k,\ell})\mathbf{M}_{k,i}\end{aligned}$$

and a vector $\mathbf{v} \in \mathbb{R}^{D(D+1)/2}$ that has entries

$$\mathbf{v}_{\iota(i,j)} = \frac{\theta}{2} + \frac{\delta_{i,j}}{2\eta_i}.$$

With this notation in hand we can then easily compute an initial state for the system. In particular, we always assume that the population is at equilibrium for some migration matrix, mutation rate, and population sizes, and use that equilibrium as the initial condition. Subsequent dynamics are due to a change in the migration matrix, mutation rate, and/or population sizes. We can compute the equilibrium by solving the matrix equation

$$-\mathbf{v} = \mathbf{G}\mathbf{m}_0. \tag{9}$$

In practice, $\mathbf{G}$ is quite sparse, and so we use sparse solvers implemented in `scipy` [5] to solve this equation.

One we have our initial condition, we then change the migration matrix, mutation rate, population sizes, or deme structure (which changes $\mathbf{G}$ and $\mathbf{v}$) and then evolve the system forward according to eq. (8). If we want to evolve the system forward by an amount of time s from t to $t+s$ and $\mathbf{G}$ and $\mathbf{v}$ do not change during the course of that time, then we can explicitly solve eq. (8):

$$\mathbf{m}_{t+s} = \exp\{s\mathbf{G}\}\left(\mathbf{m}_t + \mathbf{G}^{-1}\mathbf{v}\right) - \mathbf{G}^{-1}\mathbf{v}, \tag{10}$$

and in practice, we take advantage of the sparsity of $\mathbf{G}$ to compute the matrix exponential using the `expm_multiply` [6] function in `scipy.sparse`, and a sparse solver to compute $\mathbf{G}^{-1}\mathbf{v}$.

In all of our simulations and analyses we always consider piece-wise constant dynamics, and obtain second moments (and $\pi(t)$) via repeatedly applying eq. (10).

The exponential behavior of equilibration

Our mathematical framework also provides mathematical intuition into the dynamics of the π. In this section we will proceed non-rigorously to highlight the key features of the dynamics implied by out model. In particular, the expected value of π depends only the second moments, which we showed were governed by the linear system of ODEs in Equation 7. This system is linear and can be analytically solved resulting in Equation 10. The key feature of Equation 10 is that the only term that depends on the length of time that has passed is of the form $\exp\{s\mathbf{G}\}$.

In particular, if we suppose that $\mathbf{G}$ can be diagonalized, then there exists an invertible matrix $\mathbf{P}$ and a diagonal matrix $\mathbf{D}$ such that $\mathbf{G} = \mathbf{P}^{-1}\mathbf{D}\mathbf{P}$. In this case, $\exp\{s\mathbf{G}\} = \mathbf{P}^{-1}\exp\{s\mathbf{D}\}\mathbf{P}$.

bioRxiv preprint doi: https://doi.org/10.1101/2024.10.21.619096; this version posted October 22, 2024. The copyright holder for this preprint (which was not certified by peer review) is the author/funder, who has granted bioRxiv a license to display the preprint in perpetuity. It is made available under aCC-BY 4.0 International license.

Furthermore, since $\mathbf{D}$ is diagonal, its matrix exponential is simply the diagonal matrix formed by exponentiating its values along the diagonal:

$$[\exp\{s\mathbf{D}\}]_{ii} = \exp\{s\,[D]_{ii}\}\,. \tag{11}$$

Then, we know that second moments of the allele frequency should equilibrate to $-\mathbf{G}^{-1}\mathbf{v}$ from Equation 9. But this is precisely the part of Equation 10 that does not depend on time, and so we expect that as $s \to \infty$, the time-dependent component of Equation 10 should converge to zero. In particular, this would imply that all of the diagonal entries of $\mathbf{D}$ are real and strictly negative so that $\exp\{s\mathbf{D}\}$ goes to the zero matrix as $s \to \infty$. The rate at which $\exp\{s\mathbf{D}\}$ goes to zero will be determined by the least negative element of $\mathbf{D}$, which we will call $\mathbf{D}_{\text{max}}$. In particular, we expect the second moments to equilibrate at least as fast as exponential with rate $-\mathbf{D}_{\text{max}}$. Since the expected value of π is completely determined these second moments, it will equilibrate at least as fast as the second moments do.

Comparison to Coalescent Approach

Our approach computes $\pi(t)$ under the Wright-Fisher Diffusion. The Wright-Fisher Diffusion is equivalent in a particular sense to Kingman's Coalescent [7], and hence we could also have developed a method based on the coalescent. In particular, $\pi(t)$ can be defined as the probability that two sampled haploids have different alleles. The probability of this occurring is a function of the distribution of the time to coalescence for a pair of lineages. Analogous to how we needed to compute moments for each pair of demes, we would need to compute this distribution of pairwise coalescent times for pairs of lineages sampled from each pair of demes. For the case of neutral, biallelic loci that we consider here, the coalescent for two lineages is described by a backward in time Markov chain with an absorbing state, and hence the distribution of pairwise coalescent times is Phase-type distributed [8, 9, 10, 11]. Phase-type distributions are well-understood and it would be possible to compute the probability that a pair of individuals have the same allele at present. Yet, this Markov chain must be described by a $(D(D+1)/2+1) \times (D(D+1)/2+1)$ matrix, essentially tracking which pair of demes the lineages are in, or whether they have coalesced. As a result, to compute $\pi(t)$ one would need to solve matrix equations for *every* pair of demes from which the lineages could be sampled. Solving one of these matrix equations would require a comparable amount of time to solving eq. (7), and hence our approach is much more tractable for general migration matrices. If the migration matrix is highly structured, for example, if all of the demes are related to each other in the same way and are hence exchangeable (sometimes called the island model [12]), then the coalescent approach can take advantage of this structure and would be substantially faster than our diffusion approach.

The diffusion approach has one other advantage over the coalescent approach, in that it is far more natural to track the evolution of $\pi(t)$ through time under the diffusion approach. Indeed, under the coalescent approach, one would need recompute the pairwise coalescent time distributions using the method described in the previous paragraph for each time point at which one wanted to evaluate $\pi(t)$. Intuitively, this is because the coalescent considers lineages sampled at a fixed time and traces their ancestry backward. To understand a different time point, one would need to consider lineages sampled at that time. In contrast, we can simply evolve our second moments forward in time according to eq. (10).

bioRxiv preprint doi: https://doi.org/10.1101/2024.10.21.619096; this version posted October 22, 2024. The copyright holder for this preprint (which was not certified by peer review) is the author/funder, who has granted bioRxiv a license to display the preprint in perpetuity. It is made available under aCC-BY 4.0 International license.

References

[1] Ewens WJ. Mathematical population genetics: theoretical introduction. vol. 27. Springer; 2004.

[2] Øksendal B. Stochastic Differential Equations. 5th ed. Springer Berlin, Heidelberg; 2003.

[3] Bhatia G, Patterson N, Sankararaman S, Price AL. Estimating and interpreting FST: the impact of rare variants. Genome research. 2013;23(9):1514-21.

[4] Czech L, Spence JP, Expósito-Alonso M. grenedalf: population genetic statistics for the next generation of pool sequencing. arXiv preprint arXiv:230611622. 2023.

[5] Virtanen P, Gommers R, Oliphant TE, Haberland M, Reddy T, Cournapeau D, et al. SciPy 1.0: fundamental algorithms for scientific computing in Python. Nature methods. 2020;17(3):261-72.

[6] Al-Mohy AH, Higham NJ. Computing the action of the matrix exponential, with an application to exponential integrators. SIAM journal on scientific computing. 2011;33(2):488-511.

[7] Griffiths RC, Jenkins PA, Lessard S. A coalescent dual process for a Wright–Fisher diffusion with recombination and its application to haplotype partitioning. Theoretical population biology. 2016;112:126-38.

[8] Hobolth A, Siri-Jegousse A, Bladt M. Phase-type distributions in population genetics. Theoretical population biology. 2019;127:16-32.

[9] Hobolth A, Bladt M, Andersen LN. Multivariate phase-type theory for the site frequency spectrum. Journal of Mathematical Biology. 2021;83(6-7):63.

[10] Røikjer T, Hobolth A, Munch K. Graph-based algorithms for phase-type distributions. Statistics and Computing. 2022;32(6):103.

[11] Rivas-González I, Andersen LN, Hobolth A. PhaseTypeR: an R package for phase-type distributions in population genetics. Journal of Open Source Software. 2023;8(82):5054.

[12] Latter B. The island model of population differentiation: a general solution. Genetics. 1973;73(1):147-57.
